# Climate sensitivity of the complex dynamics of the green spruce aphid—Spruce plantation interactions: Insight from a new mechanistic model

**DOI:** 10.1371/journal.pone.0252911

**Published:** 2022-02-17

**Authors:** John H. M. Thornley, Jonathan A. Newman

**Affiliations:** 1 Centre for Nutrition Modelling, Department of Animal Biosciences, University of Guelph, Guelph, Ontario, Canada; 2 Department of Biology, Wilfrid Laurier University, Waterloo, Ontario, Canada; INRA, FRANCE

## Abstract

Aphids can have a significant impact on the growth and commercial yield of spruce plantations. Here we develop a mechanistic deterministic mathematical model for the dynamics of the green spruce aphid (*Elatobium abietum* Walker) growing on Sitka spruce (*Picea sitchensis* (Bong.) Carr.). These grow in a northern British climate in managed plantations, with planting, thinning and a 60-year rotation. Aphid infestation rarely kills the tree but can reduce growth by up to 55%. We used the Edinburgh Forest Model (efm) to simulate spruce tree growth. The aphid sub-model is described in detail in an appendix. The only environmental variable which impacts immediately on aphid dynamics is air temperature which varies diurnally and seasonally. The efm variables that are directly significant for the aphid are leaf area and phloem nitrogen and carbon. Aphid population predictions include dying out, annual, biennual and other complex patterns, including chaos. Predicted impacts on plantation yield of managed forests can be large and variable, as has been observed; they are also much affected by temperature, CO_2_ concentration and other climate variables. However, in this system, increased CO_2_ concentration appears to ameliorate the severity of the effects of increasing temperatures coupled to worsening aphid infestations on plantation yield.

## 1 Introduction

The impact of climatic change on forests, pest populations, and their interactions, has been the focus of much work over the last several decades. The biology is complex, and involves both direct and indirect effects of multiple climate variables. Elevated CO_2_, changing temperature, and their interactions have direct impacts on plant growth and quality (from the herbivores’ perspectives [1]). Many invertebrate pest species’ population dynamics are highly sensitive to ambient temperature means and variances [2]. While a great deal of empirical experimental work has been done on species of economic consequence, their long-term responses to climatic change can only really be assessed with models. In this paper, we construct a mechanistic ecosystem simulation model of the interaction between Sitka spruce trees (*Picea sitchenis*) and their herbivores the green spruce aphid (*Elatobium abietinum*).

### 1.1 The biological system

The green spruce aphid, *E. abietinum* (syn. *Aphis abietina*), is a significant pest of some species of spruce (*Picea*) in parts of Europe. Its ecology and impacts have been comprehensively reviewed by Day et al. [3]. Dixon [4] gives an excellent introduction to the science of aphids including much material relevant to the green spruce aphid. Sitka spruce (*P. sitchenis*) is an exotic species in northwest Europe; it is now the predominant plantation species in maritime areas, where it produces a yield of 9–15 m^3^ ha^−1^ y^−1^ of stem wood over a rotation—in Scotland with Sitka spruce this is typically 60 years. It is one of the most productive trees in this situation and this is being enhanced by progressive genetic gains (see preface of [3]).

The aphid feeds on phloem sap [5]. It partially defoliates but rarely kills its host; it can depress annual growth by 10–50% [6]. The impact of such infestations is generally summarized by its effect on observed yield of stem wood during and over a rotation. A rotation length of 60 years does not allow studies which directly address the problem to be easily executed. Therefore research remains mostly empirical and short term. Randle and Ludlow ([7] p. 33) state that “The ideal model for defoliation studies remains to be developed” and this seems to remain largely true (but, for studies of aphids in other systems, see Table A.1 in S1 Appendix for summary).

Interest in this particular tree–aphid system’s response to climatic change dates back to at least the mid-1990s. Straw [8] summarized the assessment at that time as follows (p. 134):

“Defoliation of Sitka and Norway spruce by the green spruce aphid (*Elatobium abietinum*) is limited in the UK primarily by periods of cold weather which reduce the number of aphids overwintering. If winters become milder, as current models of climate change predict, then the aphid is likely to become more abundant and years with severe defoliation more frequent. In such circumstances the productivity of spruce will decline.”

### 1.2 Models of aphids and climatic change

Probably due to their economic importance, aphids have been the focus of numerous modelling studies. These studies fall into three main types: statistical, agent-based, and mechanistic.

#### 1.2.1 Statistical models

In the context of climatic change, statistical models of insect responses are largely so-called species distribution models (SDMs). Popular tools include Maxent (a form of presence only logistic regression modelling), Genetic Algorithm for Rule-set Production (GARP; [9]) and several others. For a comparsion of these techniques, see [10]. While these methods have been popular for modelling the potential impact of climatic change on non-aphid insects, they have rarely been used for aphids. There are plenty of examples of SDMs for aphids (see e.g. [11–13) but it is more rare to see these coupled with projections of climatic change (but see e.g. [14, 15]).

Statistical models contain no information beyond the original data used to construct them. They say nothing about the mechanisms that give rise to the response. For example, SDMs struggle to handle species interactions, due to their lack of mechanisms. This can be particularly problematic for herbivore–plant interactions since the host plant responds dynamically to climatic change and ignoring changes in the host plant’s distribution or quality can result in very different views of the future (see e.g. [16, 17]). Also, while it seems reasonable to estimate thermal tolerances from current species distributions, SDMs are incapable of considering changes in CO_2_ concentrations, another key component of climatic change for plant-herbivore interactions [18, 19]. Nevertheless, statistical models are useful for summarizing data and interpolating between data. They are often ‘user friendly’ and can be readily fashioned into tools valuable to farmers or farm advisors.

#### 1.2.2 Agent based models

Another common modelling approach is to use spacially explicit models (SEMs), particularly agent based models. DeAngelis et al. [20] provides a useful review of the approach and its relationship to other modelling approaches. They point out that SEMs can reveal aspects of local and regional level processes that are often absent in spatially implicit models (SIMs). Their review suggests that “spatial models in ecology have largely gone in different directions: towards SEMs for applied or pragmatic problems and towards SIMs for theoretical problems” ([20] pg. 294). This is possibly due to the fact that SEMs require (or at least can make use of) detailed landscape information, the gathering of which can be a long, laborious, and expensive process [20]. Thierry et al. [21] develop a general agricultural landscape modelling framework that can be used to explore the effects of agricultural landscape dynamics on organisms. While agent-based modelling is used widely in ecology, its use for modelling aphid dynamics has been more limited.

Parry et al. [22] constructed an individual-based aphid population model. They modelled a 5 km × 5 km region of Hertfordshire in southeastern England but did not consider climatic change. Agent based models are computationally intensive. As the authors point out, a challenge for this approach is to expand it so that it can cover realistic aphid densities across larger regions, which will increase the run-time and computational power required. Wiest et al. [23] simulated the population growth of the *Rhopalosiphum padi* in wheat plants exposed to environments with different thermal regimes. Population size varied according to the thermal regime. The effects of constant, daily variation, and outside mean minimum/maximum air temperature thermal regimes on the development and fecundity rates were not uniform. Although this model could be used to explicitly study the impacts of climate change, the authors did not do so. Picaza et al. [24] used an agent based model to study aphids as disease vectors, but did not model temperature dependent population growth and so it is not suitable for the study of climate change impacts.

#### 1.2.3 Mechanistic models

There is a long tradition of using mechanistic models to study aphid population dynamics. Table A.1 in S1 Appendix summarizes a sampling of these models. Commonalities found in these models are readily apparent. First, with the exception of Newman et al. [25–29] these previous models have not been designed for, or used for, studying the impacts of climatic change (but see [30]). Second, since ‘climate change’ encompasses, *at a minimum*, changes in air temperature and CO_2_, none of the previous models are even suited to the task because they do not consider the effects of rising CO_2_ (again, with the exception of the Newman et al. studies). Third, with the exception of Barlow et al. [31–33] and a very basic model by Day et al. [3], none of previous models have considered tree aphids and even these models lack a mechanistic treatment of tree growth and physiology. And fourth, even for models that do include a consideration of the host plant, many of these models do not dynamically link the aphid and the host plant. That is, the models of the plants tend to be very simplified and unresponsive to aphid pressure. On the other hand, Newman et al. [25] constructed a model of cereal aphid population dynamics and coupled it to the Hurley Pasture Model [34, 35], a long-established mechanistic ecosystem simulation model of temperate grass pastures. They [25] used this model to gain insight into the generality of aphid population dynamic responses to climatic change, to understand the magnitude and direction of each of the climate variables’ impacts, and to explore the role of predation in controlling aphid populations under climate change [25–29].

Our objective here is to construct a transparent mechanistic model of the green spruce aphid and interface this with a long-established mechanistic forest ecosystem simulation model, the Edinburgh Forest Model (efm, [36]). We then examine the climate sensitivity of the model’s predictions. We believe that an understanding of green spruce aphid dynamics can only be obtained by combining a mechanistic aphid model with a mechanistic plant growth model. No tuning (or less charitably—‘parameter twiddling’) has been applied. At this stage, it is arguably more valuable to examine the range of behaviour the model can predict, than to look for an understanding of the discrepancies which may exist between observation and theory.

## 2 Tree sub-model

**Fig 1:**
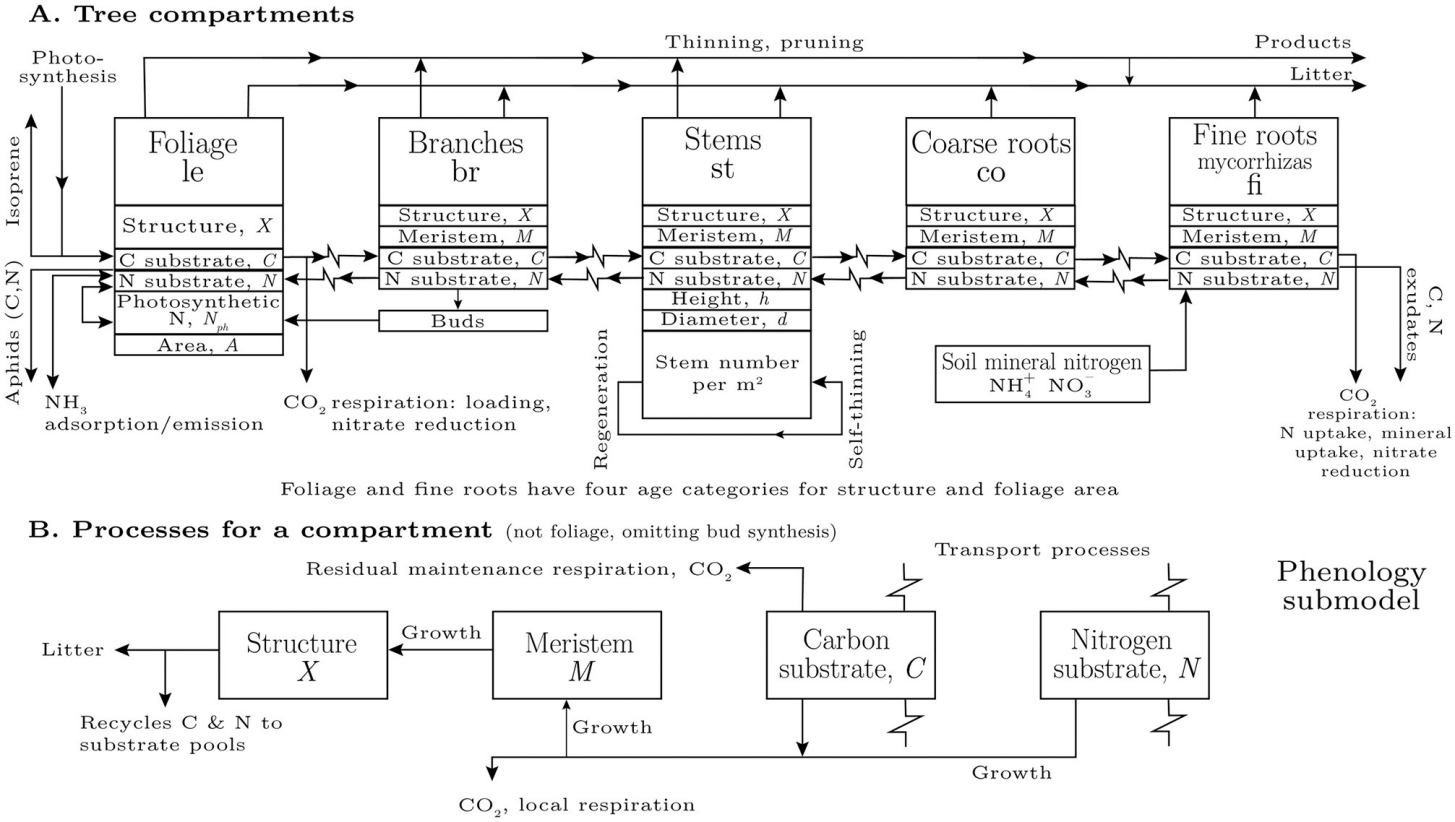
Tree sub-model of the Edinburgh Forest Model (efm). Aphids are connected to the tree sub-model on the left side in A, where aphids extract C and N from the foliage (leaf, le) substrate pools, denoted *C*_le_ and *N*_le_, via the phloem [Eq (2)]. The aphid sub-model can be ‘switched off’ entirely (the default position).

The aphid sub-model is interfaced with the Edinburgh Forest Model (efm). The efm is a mature and well-validated mechanistic simulator applicable to evergreen or deciduous forest ecosystems ([36]; see Appendix B in S1 Appendix below for the numerical methods employed). These can be grown as plantations, managed forests, or unmanaged forests. The model is based on simplified physiology and biochemistry with soil and water sub-models. The efm couples carbon (C), nitrogen (N) and water, fluxes and pools and provides stoichiometric balancing of the items represented. The efm is shown schematically in Fig 1 and sketched in some mathematical detail in Appendix C in S1 Appendix.

## 3 Overview of aphid sub-model

**Fig 2:**
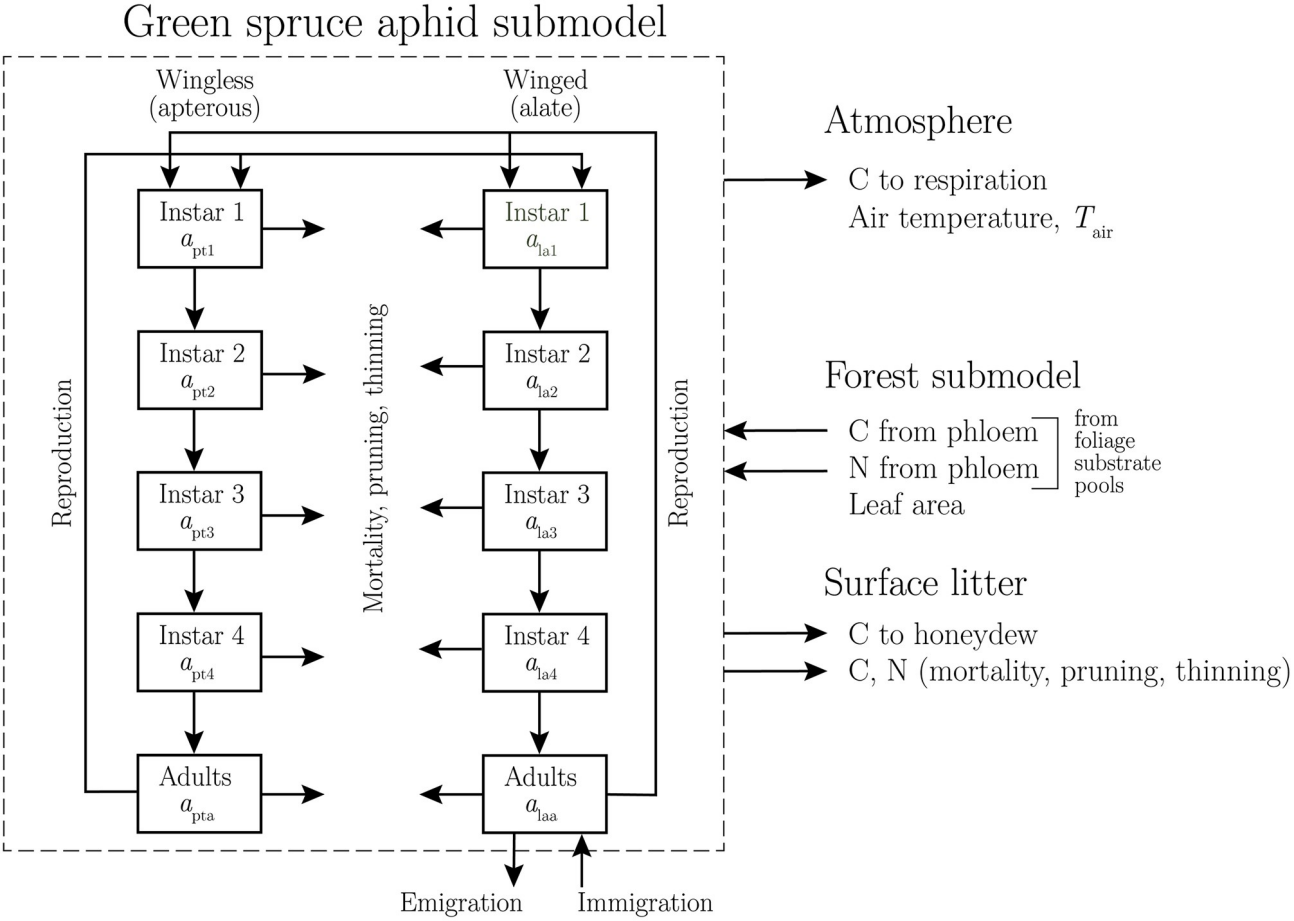
The green spruce aphid sub-model. The ten state variables of the sub-model are shown in the boxes. There are four instars and an adult form for the wingless (apterous) and the winged (alate) aphids. Units for the state variables are number of aphids per stem (Appendix D in S1 Appendix). The differential equations for the state variables are Eqs (16), (47), (64) and (87).

In this section we give an overview of the aphid sub-model, which has ten state variables as shown in Fig 2. Details of the ordinary differential equations (ODE’s) which determine their dynamics, the fluxes which contribute to the ODE’s and their behaviour are given in detail in Appendix D in S1 Appendix. In brief, aphids extract C and N from the foliage substrate pools via the phloem. This reduction in plant C and N reduces tree growth and is expressed as reduced leaf area. Aphids return C to the atmosphere via respiration, and to the surface litter via honeydew production. Pruning (which kills aphids feeding on the pruned foliage), thinning (which kills aphids feeding on the thinned trees), and aphid mortality all return C and N to the soil which can then be respired or absorbed by the trees and soil microorganisms. As far as we can tell, no previous aphid population model is able to track the fate and transport of these nutrients between plant, aphid, and soil.

## 4 Simulation scenarios

### 4.1 The environment

**Fig 3:**
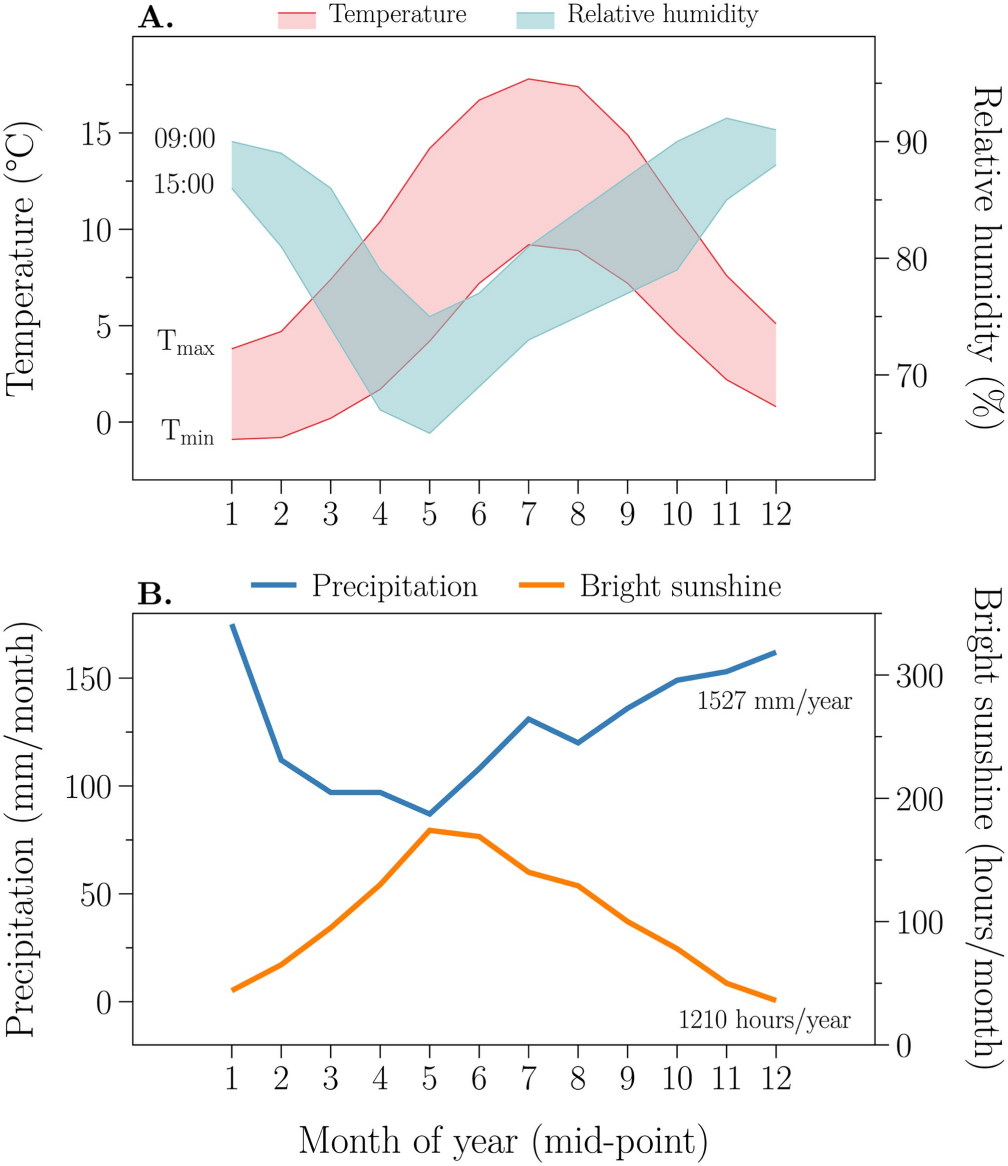
Climate at Eskdalemuir. 30-year monthly means taken from meteorological tables are drawn. See text for details.

To illustrate the model’s dynamics, we used the average climate for Eskdalemuir, in northern Britain, latitude 55 19′ N, longitude 3 12′ W, altitude 242 m above sea level (Fig 3). The monthly data [37] were supplemented by monthly data from Clino [38]. Daily values were obtained by linear interpolation of monthly data (see chapter 7 and Section 7.5.3 of Thornley [34]). Daily data were applied to give diurnally changing data for air temperature (Fig 3A), relative humidity (Fig 3A), radiation (which is calculated from sunshine hours; Fig 3B), and wind (not shown). Soil temperature and rainfall were assumed constant over each day. The source program, efm.csl, gives details (see Appendix C in S1 Appendix).

Wind speed at 50 m reference height (*h*_ref_) varies seasonally with the maxima of the daily maxima and daily minima occurring on 26 April. The mean and the variation of the daily maximum wind speeds are 6 and 1.5 m s^−1^. The mean and the variation of the daily minimum wind speeds are 2 and 0.5 m s^−1^. The mean annual wind speed is 1/2×(6+ 2) = 4 m s^−1^. We assumed that the diurnal variation of wind speed is similar to that of air temperature (*T*_air_) and opposite to that of relative humidity (RH): the minimum wind speed (and minimum temperature and maximum RH) occurs at dawn and the maximum wind speed (and maximum temperature and minimum RH) at 15 h.

Photosynthetically active radiation (PAR) varies from a minimum of 0.55 on 20 December to a maximum of 7.9 MJ PAR m^−2^ d^−1^ on 15 June. It is calculated from bright sunshine hours (Fig 3B) by using a version of the Ångström formula ([34], pp. 145–146, equation 7.4i, but with *a*_Ang_ = 0.19 and *b*_Ang_ = 0.62; [39]) for daily PAR light receipt, *j*_PARdy_ (J m^−2^ d^−1^) from the fraction of bright sunshine hours which has been interpolated from monthly values (Fig 3B) to give daily values and refers to a given Julian day number.

Soil temperature was assumed to be diurnally constant and equal to mean daily air temperature; it varies from a minimum of 1.45°C on 16 January to a maximum of 13.5°C on 16 July. Diurnal air temperature variation is a maximum of 5°C on 16 May and a minimum of 2.15°C on 16 December.

Relative humidity (RH) was assumed to be at its daily maximum at dawn and at its daily minimum at 15 h. The RH daily maximum has a minimum of 0.75 on 16 May when the RH daily minimum is 0.65 and the RH daily minimum has a maximum of 0.88 on 16 December when the RH daily maximum is 0.91.

Daily rain fall (assumed to be diurnally constant) is substantial, varying from a minimum of 2.8 kg (2.8 mm) m^−2^ d^−1^ on 16 May, to a maximum of 5.65 kg (5.65 mm) m^−2^ d^−1^ on 16 January, with an annual rain fall of 1527 kg (1527 mm) m^−2^ y^−1^ (see Fig 3A).

### 4.2 Forest plantation

A regime of planting to an initial stem density of 0.25 stems m^−2^ and an initial leaf area index of 0.003 [Eq (1)], followed by the removal of 0.45, 0.4, 0.35, 0.3, 0.25, 0.2, 0.15 and 0.1 of the existing stems at times of 20, 25, 30, 35, 40, 45, 50 and 55 years and terminated by clear felling at 60 years, was employed. Stem removal takes place at a constant rate over a period of 1 d (1 January normally) and this determines the thinning function *O*_nstems,*th*_ of Eq (1), which depends on the integration step, maxt (Appendix B in S1 Appendix).

### 4.3 Climate change simulations: Temperature and CO_2_

We examined the model output for temperatures ranging from −3 to + 4°C below and above the ambient Eskdalemuir temperatures. In some places we present results for a restricted subset of temperatures in the interest of brevity. In those cases we leave out the two extreme changes (−3 and + 4°C). We examine further the interaction of temperature change with rising atmospheric CO_2_ concentrations. Two CO_2_ concentrations were applied: 350, and 700 μmol mol^−1^. Although current ambient CO_2_ is now above 400 μmol mol^−1^, we chose 350 μmol mol^−1^ as ‘current ambient’ to aide comparison with previous experimental and theoretical research. Initial values for all efm state variables were equilibrium with no aphids for the temperature and CO_2_ level applied. The equilibrium is a 60-year repeating equilibrium; the same 60-year rotation period is applied to all the temperature × CO_2_ scenarios considered, although this would not usually give the optimum timber yield (yield class, *Y*_C_) for all cases.

Another widely studied impact of climatic change is altered precipitation patterns. We note that the present model can be used, without alteration, to study such effects, and indeed to study the three-way interaction between precipitation, temperature and CO_2_. The model could also be driven by the outputs of a general or regional circulation model for added ‘realism’. However, such uses are beyond the scope of the present paper. Here, as we stated earlier, our goal is to examine the range of behaviour the model can predict, not to look for an understanding of the discrepancies which may exist between observation and theory.

## 5 Results

### 5.1 Aphid population dynamics

**Fig 4:**
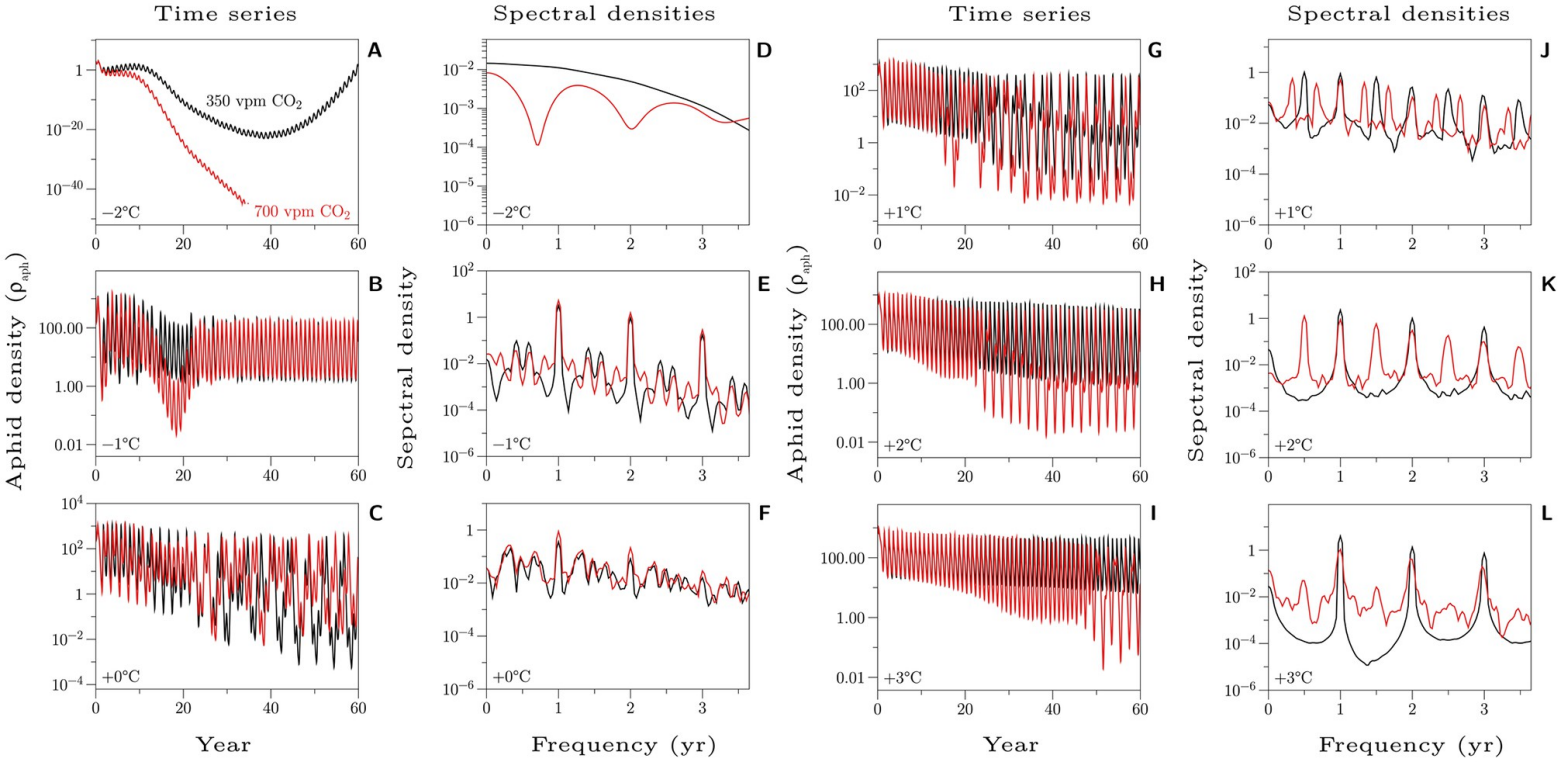
Aphid density dynamics. Shown are the time series of aphid densities (ρ_aph_) for incremental increases in temperature from −2°C to + 3°C. 350 vpm CO_2_ is shown in black, and 700 vpm CO_2_ is shown in red. Notice the qualitative differences in population dynamics that emerge as temperature and CO_2_ change. Next to each time series is the respective spectral density (arbitrary units) calculated from the last 32 years of each time series; the frequency (*x*-axis) has been re-scaled to display in years. Spectral analysis is a set of techniques that use Fourier transforms to detect frequency patterns in time series data. It is used to probe the underlying structure in dynamics systems. See, e.g., McBurnett [40] for an introduction. For mathematical details see https://www.jmp.com/support/help/en/16.0/index.shtml#page/jmp/statistical-details-for-spectral-density.shtml.

In Fig 4 the aphid population densities (ρ_aph_) are plotted together, 700 vpm CO_2_ (red) overlaid on 350 vpm CO_2_ (black), for each incremental temperature change (Δ*T*) from −2 to + 3°C for the full 60 year rotation. Also shown are the corresponding spectral densities calculated for the final 32 years of the rotation. Peaks in the spectral density plots indicate periodicity [40]. For example, Fig 4K shows that both time series have annual cycles, but that they out of phase with each other by about 6 months.

Fig 4 demonstrates quantitative differences in population dynamics. The largest peak abundances occur between current ambient temperature and + 1°C in either CO_2_ condition. Perhaps not surprisingly, the smallest peak abundances occur at −2°C, particularly under elevated CO_2_. Nevertheless, our focus in this paper is on the range of behaviour exhibited by this dynamic system rather than on the magnitudes of the response. To this end, Fig 4 clearly demonstrates *qualitative differences* in the population dynamics. At −2°C, aphids appear to die out well before the 60 year rotation is complete (they are still present, but at vanishingly low densities), and the die out happens faster under 700 vpm CO_2_. At −1°C, both CO_2_ conditions transition to the same, reasonably stable, annual cycles of relatively low aphid densities (ρ_aph_), although they do so on different trajectories and the spectral density shows that there is additional underlying within year structure. At ambient temperature (Δ*T* = 0), both CO_2_ conditions begin with annual cycles but diverge and transition to chaotic dynamics. The corresponding spectral densities have no obvious structure, indicative of (though not determinative of) chaos. At + 1°C, aphids transition from annual cycles to more complex periodicities. The spectral densities show that the periodicities are different and out of phase with each other. At + 2°C we see a similar pattern, but with stronger signals of periodicity. Finally, at + 3°C and 350 vpm CO_2_, aphids again show sustained 2-point cycles, while at 700 vpm CO_2_ the annual cycles are supplemented with complex within year dynamics.

**Fig 5:**
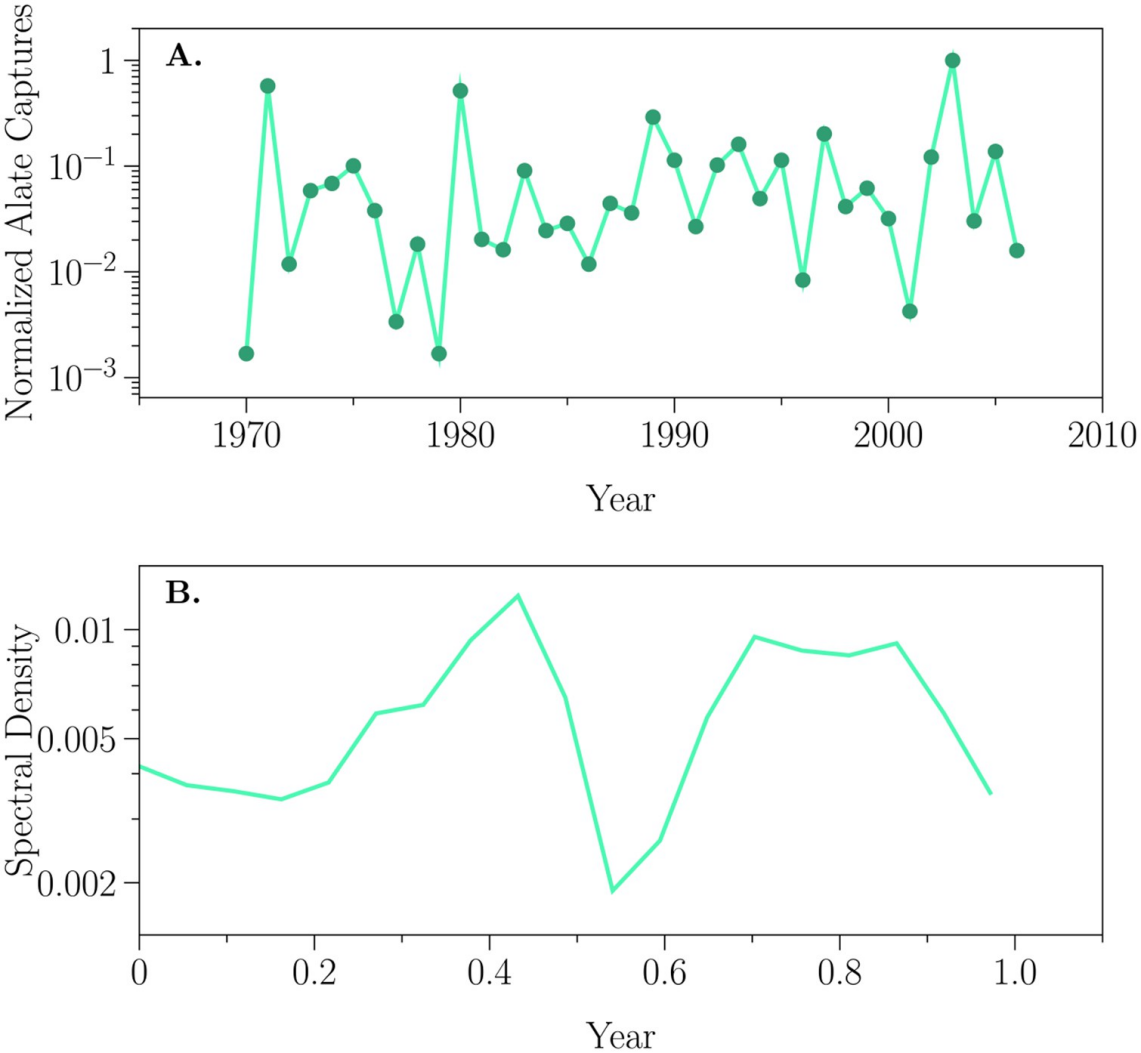
Alate density dynamics. Shown are (A) the time series of alate aphid captures (dots; normalized to the maximum number of captures, see Day et al. [41] for more details) and (B) the associated spectral density function.

We can compare the dynamics depicted in Fig 4 to those seen in Fig 5. Here we plot the normalized trap captures of alate *E. abietinum* using data extracted from Day et al. [41]. We can see that this time series too contains periodic structure verging on chaos. The spectral decomposition has less structure than we see in our simulations. While we show the spectral decomposition for the same length of time (32 years) our ‘sampling’ of that period is 73 times more dense. Nevertheless, Fig 5 shows one feature that is also seen in our simulations, within year cycles (see e.g., Fig 4 + 1°C, either vpm CO_2_ concentration).

### 5.2 Leaf area index (*L*_AI_)

**Fig 6:**
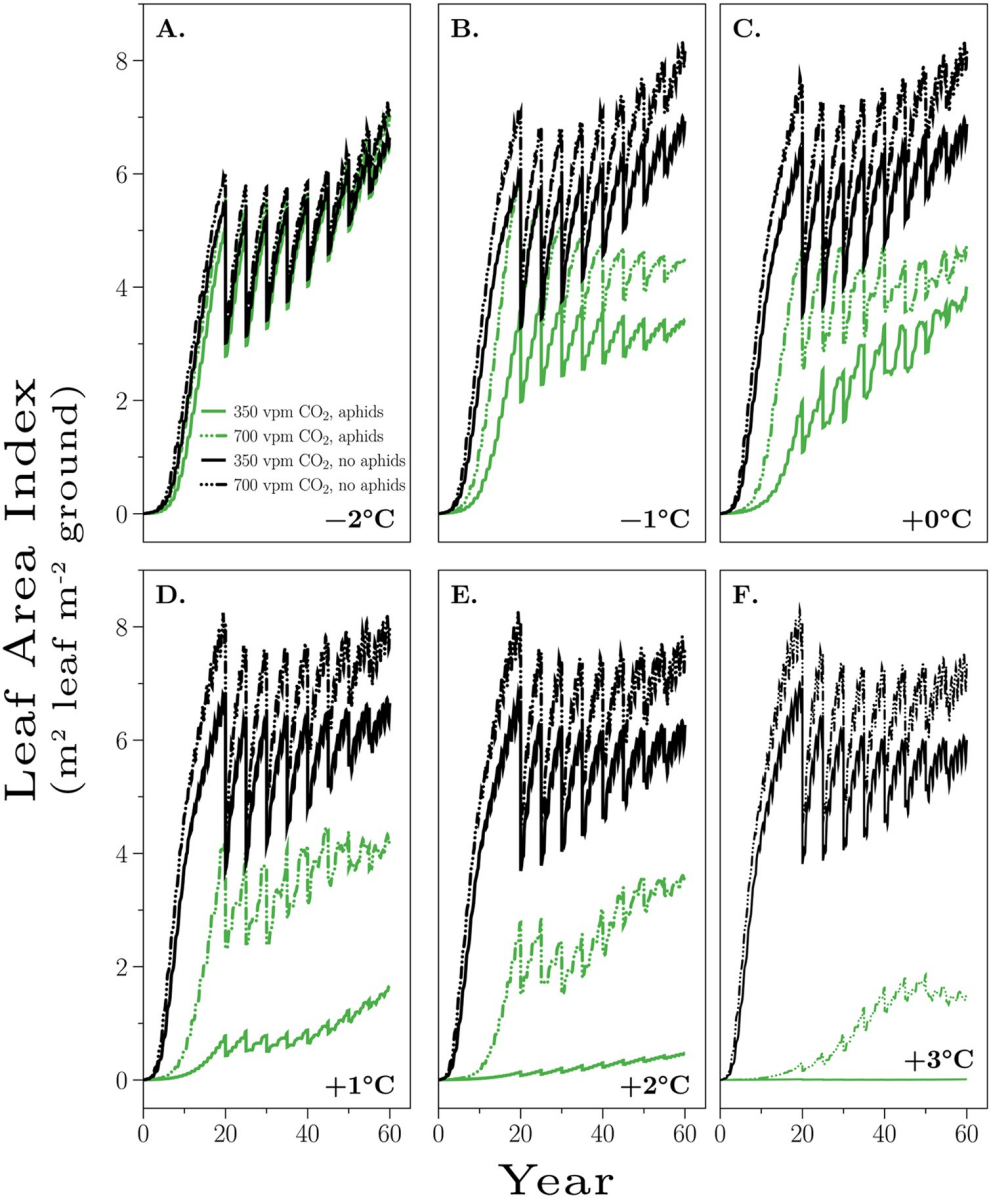
Leaf area indices. Shown are the Leaf Area Indices (m^2^ leaf m^−2^ of ground), Eq (1), for incremental temperature changes at 350 vpm and 700 vpm CO_2_, with and without aphids. The LAI values result from changes in aphid density dynamics, see Fig 4.

Fig 6 shows the Leaf Area Indices that result from the aphid density dynamics. *Without* aphids at 350 vpm CO_2_, increasing temperature generally increases the resulting LAIs, although toward the end of the 60 year rotation the ambient temperature ends up resulting in the highest LAI. Doubling the CO_2_ results in greater values of LAI for all temperature increments, although by the end of the rotation the different temperature trajectorys largely converge. *With* aphids, we see striking differences. At 350 vpm CO_2_, we see a complete reversal in the ordering of the LAIs. Now, −2°C results in the greatest LAI because aphid population densities are the lowest in these conditions (Fig 4A). A doubling of CO_2_ while generally resulting in higher LAIs does not fundamentally change the conclusion. −2°C still results in the greatest LAI, because aphid densities are still lowest at this temperature (Fig 4A).

### 5.3 C-sequestration (*C*_sys_) and plantation yield (*Y*_C_)

**Fig 7:**
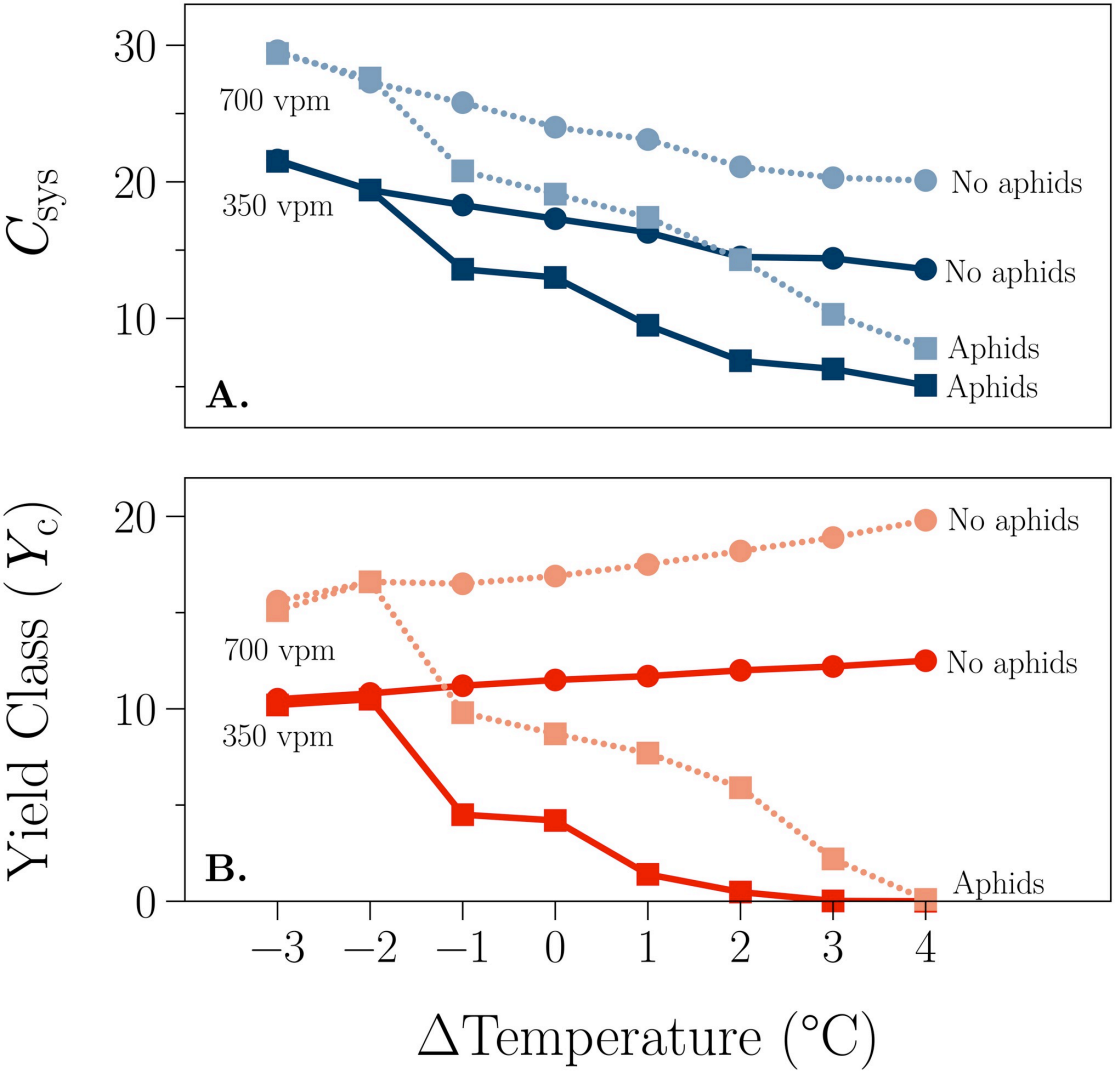
Yield and C-sequestration. ΔTemperature is the increment in air and soil temperatures applied to the Eskdalemuir environment (Section 4.1, Fig 3). The CO_2_ concentration is denoted by the shading (dark color: 350 vpm, light color: 700 vpm). The aphid condition is denoted by the symbol (●: without aphids, ∎: with aphids). *Y*_C_ is the yield class (m^3^ ha^−1^ y^−1^), defined as the volume (m^3^) of timber harvested at the end of a rotation per hectare averaged over the duration of a rotation of 60 years; *C*_sys_ (kg C m^−2^) is the total C in the system at the end of the 60 y rotation. Results are shown for the no-aphid infestation situation (a steady state as applied in Fig 4) and for that when aphids are applied at time zero [Eq (64)].

The carbon-sequestration (*C*_sys_) and plantation yields (*Y*_C_) follow from the effects of aphids density dynamics, and temperature and CO_2_ concentration changes. Fig 7 shows the four combinations of aphid presence and CO_2_ for each temperature increment. It is clear from the figure that doubling CO_2_ results in greater C-sequestration and greater plantation yields at temperatures of −1°C to + 4°C. The presence of aphids results in lower C-sequestration and lower plantation yields. In general these two effects seem to be approximately additive.

## 6 Discussion and conclusions

### 6.1 Climate sensitivity of aphid population dynamics

The dynamics of the aphid populations is interesting due to its variety, ranging from aphids dying out (Fig 4A, −2°C) to various degrees of chaos with annual, biennial and triennial cycles (Fig 4). It is not surprising that chaos occurs rather robustly in the aphid-plant context. There is much non-linearity prevalent; there are far more than the minimum two state variables required for chaos; the equations are non-autonomous; and the intrinsic time scales of the dynamics of the aphid sub-model and the tree sub-model are very different and incommensurate [42, 43]. This chaotic variety indicates that focusing on management/control strategies in order to minimize adverse consequences may be difficult, even with the assistance of a process-based model (or further consideration of chaotic aphid population dynamics see [44–48]). Rutherford Aris [49] succinctly summed up this problem as follows (pp. 25–26):

“[Chaos] differs from a random process in the following sense. In a random process the attempt to predict future states is limited by the range of the correlation of the random process, whereas in a chaotic process it is limited by the accuracy with which the initial conditions can be determined. This is the case because arbitrarily near to the initial point of any solution there are infinitely many initial points that will give solutions that ultimately diverge completely from the first solution. But this raises the question whether the matching of results of a computation with experience can ever be trusted. Such questions as: does a mismatch constitute an adverse reflection on the model or is it only result of a failure to find the initial conditions with sufficient accuracy? could a chaotic solution be distinguished from one with a very long period? even if such a distinction could be made, would it matter? how can two models ever be compared if their solutions are both chaotic? These and other questions are as yet unanswered but are relevant to the question of evaluating a model.”

### 6.2 Comparisons with modeling studies

The model described here is far from the first model to describe the population dynamics of aphids using simulation modelling. Appendix A in S1 Appendix summarizes 40 aphid simulation modelling studies. Where the present model differs from all but Newman et al. [25, 29] is in (*a*) the treatment (mechanistic or otherwise) of the impacts of rising CO_2_ on plant growth and (*b*) the use of the model to study impacts of climatic change. Both the present study and Newman et al. found that the mechanistic consequences of rising temperature tends to benefit aphid population growth while rising CO_2_ tends to be detrimental due to the changes in the plant caused by the rising CO_2_ (see also [26]).

Although there are similarities between the present model and that of Newman et al. [25], they differ in several important respects. For example, Newman et al.’s model is not stochiometrically complete as is the current model. We believe that this is the first model to provide a complete accounting of the cabon and nitrogen in a full ecosystem simulation of plant-aphid interactions. While many of the aphid models described in Appendix A in S1 Appendix could be coupled with either the efm or the Hurley Pasture Model, none would provide such a complete accounting. Newman et al. also used their model to answer different questions, focused almost exclusively on the question of changes in aphid abundance. However, perhaps the most significant difference between the two studies is that Newman et al. use their model to study *within* year dynamics, while we used the present model to study an entire 60-year plantation rotation. By studying the dynamics over such a long period of time, we were able to predict the responses illustrated in Fig 4, which range from aphids dying out at low temperatures, to aphids having a severe impact on the productivity of managed spruce plantations (Fig 7B). Our work suggests that this may be a promising approach to investigating the impact of aphids on plant ecosystems in a changing climate. Our simulations indicate that, while temperature is the most important environmental variable, higher CO_2_ levels ameliorate the impact of aphid infestation on yield and carbon sequestration (Fig 7) and on leaf area index (Fig 6) and aphid dynamics (Fig 4). None of the previous modelling exercises have been able to provide such an integrated picture of the possible impacts of climatic change, let alone for plantation ecosystems.

### 6.3 Model criticism, limitations and extensions

The aphid model is so far untuned for any specific purpose although where possible parameter values were estimated for the green spruce aphid. Our analysis leans heavily on Dixon [4] and on Day et al. [3] who focus specifically on the green spruce aphid. While we have tried to tie parameter values down to data and predictions to observations, this has been difficult, perhaps due to the traditions in this area of work. As mentioned earlier, we have not done any indiscriminate ‘parameter-twiddling’, which can be an endless process (but see Hjelkrem et al. [50] who provide a method of Bayesian calibration which could help to overcome some problems of indiscriminate ‘parameter-twiddling’). With any model, it is preferable to fix parameters by experiments targeted at the level of the parameters than by adjustment with reference to the outcomes predicted by the model which depend on everything within the model; we know that all models are wrong in some respect. We have found that the occurrence of chaos and our general results are qualitatively not greatly affected by the details of parameterization, so long as the values assumed are approximately correct. The main conclusion is, we suggest, that mechanistic aphid-plant models may be an essential but difficult approach to understanding how these systems work.

## Supporting information

S1 Appendix (PDF)
