# Supplementary material for: Climate sensitivity of the complex dynamics of the green spruce aphid—Spruce plantation interactions: Insight from a new mechanistic model
Source: PLoS One. 2022 Feb 17;17(2):e0252911. doi: 10.1371/journal.pone.0252911 (PMC8853561; doi:10.1371/journal.pone.0252911)
Supplement: S1 Appendix — (PDF) [file pone.0252911.s001.pdf]

Appendix A Aphid simulation models

Tab A.1. Summary of aphid simulation models. 'TD aphid' denotes that aphid growth is represented in the model as temperature-dependent; similarly 'TD plant' denotes that plant growth is represented as temperature-dependent. 'CO_2 plant' denotes whether the model considers the CO_2 concentration and its mechanistic effects on plant growth. 'Climate Δ' denotes whether climate change is explicitly considered or not. The 'Stated purpose' indicates the purpose of the model according to the authors. '??' denotes that the information was not presented in the noted reference.

Reference (Year)	TD aphid	Plant species	TD plant	CO_2 plant	Climate Δ	Stated purpose
**Aphids that feed on graminoid host plants**						
**Bird cherry-oat aphid (*Rhopalosiphum padi* L.)**						
Wiktelius & Pettersson [1] (1985)	Yes	None	N/A	N/A	No	"A model of the population dynamics of the bird cherry–oat aphid (*Rhopalosiphum padi* (L.)) has been used to study the likely effects of plant resistance on aphid population growth."
Ekbom et al. [2] (1992)	Yes	Spring barley	Yes	No	No	"The models were used to answer several questions of importance for evaluation of naturally occurring control. 1) Can polyphagous predators find aphids at very low prey densities? 2)Is the timing of predation in relation to the aphid's phenology important for resulting population development? 3) What are the necessary conditions for effective control by natural enemies?"
Morgan [3] (2000)	Yes	Winter barley	No	No	No	"The aims of this paper are to utilize simulation analysis techniques to develop a model describing the population development of *R. padi*, to use the model to examine the relative importance of various population processes in pest dynamics and to determine whether the model could be used as a basis for forecasting aphid outbreaks."
Newman et al. [4–8] (2003–2006)	Yes	Grass	Yes	Yes	Yes	"In this paper, we develop a mechanistic mathematical model of the general interaction between grasses, cereal aphids and their parasitoids. We used this model to investigate the interacting effects of rising CO_2 and temperature."
Parry et al. [9] (2006)	Yes	None	N/A	N/A	No	"[Used] to investigate the effects of different pesticide regimes."
Thackray et al. [10] (2009)	No	Wheat	No	No	No	"BYDV PREDICTOR, a simulation model, was developed to forecast aphid outbreaks and Barley yellow dwarf virus (BYDV) epidemics in wheat crops in the grainbelt region of southwest Australia."

continued on next page

continued from previous page

Reference (Year)	TD aphid	Plant species	TD plant	CO_2 plant	Climate Δ	Stated purpose
**Russian wheat aphid (*Diuraphis noxia* Kurdjumov)**						
Ma et al. [11] (2008)	Yes	Barley	Yes	No	No	None stated
**English Grain Aphid (*Sitobion avenae* Fabricius)**						
Carter et al. [12, 13] (1980)	Yes	Winter wheat	Yes	No	No	"[Used] to explain the population development of *S. avenae* on cereals and to indicate how cereal aphid outbreaks might be predicted."
Rabbinge et al. [14, 15] (1979)	Yes	Wheat	Yes	No	No	"[D]eveloped simulation models that explain the population movements of the most important aphid *S. avenae* and may be used for extrapolation and prognosis in the short run, approximately 3-5 weeks."
Skirvin et al. [16] (1997)	Yes	None	N/A	N/A	Yes	"This paper describes a mechanistic computer model of the interaction between the cereal aphid *Sitobion avenae*, . . . and its main predator in Britain, the coccinellid *Coccinella septempunctata*."
Rossing [17] (1991)	Yes	Winter wheat	Yes	No	No	"To evaluate the relative importance of various components of damage caused by grain aphid (*Sitobion avenae* E) populations in winter wheat, a simulation model of crop growth and development is combined with a model of aphid injury."
Freier et al. [18, 19] (1996, 2001)	Yes	Winter wheat	Yes	No	No	"[Used] to study and demonstrate tri-trophic interactions, particularly aphid population dynamics influenced by antagonists under different natural and man-made conditions, but not to predict future aphid infestation."
Duffy et al. [20] (2017)	Yes	Cereal crop	No	No	No	"[N]ew simulation model that quantifies the size and timing of grain aphid populations in response to temperature."
Rossing [17] (1991)	Yes	Winter wheat	Yes	No	No	"A dynamic, process-based simulation model is used to evaluate the effect of injury by *Sitobion avenae* on yield of winter wheat at a range of attainable yield levels."
van Roermund et al. [21] (1986)	Yes	Winter wheat	Yes	No	No	"To study the effect of the English grain aphid, *Sitobion avenae* (F.), on dry matter accumulation in winter wheat grains, the influence of aphids on plant physiological parameters was introduced into a simulation model of wheat growth and development from anthesis onwards."
**Aphids that feed on forb host plants**						
**cabbage aphid (*Brevicoryne brassicae* L.)**						
Hughes & Gilbert [22] (1968)	Yes	None	N/A	N/A	No	"[T]ool to understand the combined effects of species interactions and environmental factors on population growth
Gilbert & Hughes [23] (1971)	Yes	None	N/A	N/A	No	". . . refinement used to examine biocontrol strategies."

continued on next page

continued from previous page

Reference (Year)	TD aphid	Plant species	TD plant	CO_2 plant	Climate Δ	Stated purpose
Raworth [24] (1984)	Yes	kale	Yes	No	No	"This paper consolidates information from the previous studies in the form of a simulation model."
**pea aphid (*Acyrthosiphon pisum* Harris)**						
Gutierrez et al. [25] (1984)	Yes	Alfalfa	??	No	No	"[E]xpands a model for energy or biomass assimilation by individuals, that has been proposed in various forms in the literature, to model the growth, development, reproduction, ageing and survivorship of the pea aphid."
Snyder & Ives [26] (2003)	No	None	N/A	N/A	No	"We investigated how specialist and generalist natural enemies differ as biological control agents of pea aphids (Acyrthosiphon pisum), and how interactions among natural enemies affect successful control."
**black bean aphid (*Aphis fabae*)**						
Kindlmann [27] (1985)	No	None	N/A	N/A	No	not stated
**cotton aphid (*Aphis gossypii*)**						
Xia et al. [28] (2018)	Yes	None	N/A	N/A	No	"To better understand and characterize the predator–prey system dynamics under field conditions."
**cowpea aphid (*Aphis craccivora* Koch)**						
Gutierrez et al. [29, 30] (1974)		Clover	Yes	No	No	"... describing the interactions of various components of the biology of cowpea aphid (*Aphis craccivora* Koch), its host plant, Subterranean Clover Stunt Virus (SCSV)."
**green peach aphid (*Myzus persicae* Sulz.)**						
Whalon & Smilowitz [31] (1979)	Yes	Potato	No	No	No	"This is a report of predictive temperature-dependent density equations for green peach aphid field populations."
Tamaki & Long [32] (1978)	Yes	None	N/A	N/A	No	"A model to evaluate potential reductive impact of populations of green peach aphid, *Myzus persicae* (Sulzer), by predators was generalized to include effects of temperature on the rate of aphid reproduction and the functional response of the predator complex."
Ro & Long [33] (1999)	Yes	Potato	No	No	No	"[T]he model describes the interaction among the green peach aphid population, its predators, and abiotic factors (e.g. temperature). The model includes functions for population growth of the aphid and its predator complex."

continued on next page

continued from previous page

Reference (Year)	TD aphid	Plant species	TD plant	CO_2 plant	Climate Δ	Stated purpose
			**potato aphid (*Macrosiphum euphorbiae* Thomas)**			
Bertschinger et al. [34] (1995)	Yes	Potato	Yes	No	No	"A model (EPIVIT) was developed for the simulation of potato harvest infection (% infected tubers) with a contct- or aphid-transmitted virus."
			**soybean aphid (*Aphis glycines* Matsumura)**			
Miksanek & Heimpel [35] (2019)	Yes	None	N/A	N/A	No	"We apply this approach to investigate the effect of parasitism by the Asian parasitoid Aphelinus certus on its host, the soybean aphid (*Aphis glycines*)."
			**Aphids that feed on tree host plants**			
			**green spruce aphid (*Elatobium abietinum* Walker)**			
Day et al. [36] (2010)	Yes	None	N/A	N/A	No	"[T]he patterns of insect abundance on trees have never been satisfactorily described by a numerical model despite considerable knowledge of endogenous and exogenous factors in the population dynamics of the species."
			**lime aphid (*Eucallipterus tiliae* L.)**			
Barlow [37–39] (1979–1981)	Yes	Lime tree	No	No	No	"This paper considers two approaches to the modelling of aphid populations. Using the lime aphid (*Eucallipterus tiliae* L.) as an example, it discusses the well-established role of detailed simulation models in the study of aphid population dynamics then considers the possible application of a simple, general herbivore/plant model to aphids."
			**Aphids that feed on vine host plants**			
			**thimbleberry aphid (*Masonaphis maxima* Mason)**			
Gilbert & Gutierrez [40] (1973)	??	Thimbleberry	??	??	No	"[To] explore effects of parasitism and competition on aphid population growth."

Appendix B Numerical methods

The efm is programmed in ACSL, Advanced Continuous Simulation Language, a highly readable fortran-like language (Aegis Research Corporation, Huntsville, AL - email: acsl-sales@aegisrc.com) and is extensively commented. ACSL follows the long established CSSL (Continuous System Simulation Language) standard ([41])—designed for models which can be represented by ordinary differential equations using the 'rate-state' formalism (rate of change is a function of state variables + driving variables + parameters). A significant advantage of ACSL is that it is non-procedural—that is, the statements can be placed in any order; in the first step, translation, places the statements in an executable order, which can then be compiled and linked as usual. The non-procedural capability allows the programmer to put the statements in an order that makes biological sense. The version of ACSL used here is 11.8.4—now unfortunately legacy software, although to our knowledge there is no adequate replacement. We have used double precision (single precision fails), with translator and runtime table sizes of 200000. The compiler used is Compaq Visual Fortran 6.6-1877-47BAQ, compiling in 36 s. The linker is Microsoft (R) Incremental Linker Version 6.00.8447. Those wishing to use this excellent and much used software system, may contact Professor Parsons at Massey University, New Zealand: `a.j.parsons@massey.ac.nz`). The program code, efm.csl, which includes the aphid sub-model as an option, is freely available from GitHub (DOI: 10.5281/zendo.5910593). The time step (maxt) is about 11 minutes (1/128 d) and Euler's method of integration is applied ([42], pp. 27-40). It takes about eight seconds to simulate one year on a 1.6 GHz pc running under Windows 7 with a 32-bit operating system. We are currently translating the ACSL code to Python and will place it in GitHub when it is ready.

Appendix C Edinburgh Forest Model

We couple the aphid sub-model (see next section) with the Edinburgh Forest Model (efm). The efm is a mature and well-validated mechanistic simulator applicable to evergreen or deciduous forest ecosystems ([43]. See Appendix B for the numerical methods employed). These can be grown as plantations, managed forests, or unmanaged forests. The model is based on simplified physiology and biochemistry with soil and water sub-models. The efm couples carbon (C), nitrogen (N) and water, fluxes and pools and provides stoichiometric balancing of the items represented.

Here we used the efm in the evergreen forest mode, parameterized for Sitka spruce growing in a north British environment (Section 4.1, see main text). The elements of the efm relevant to the aphids are described here. In evergreen mode, foliage is always present and the aphids can live on the foliage year-round, with (in some situations) negligible immigration and emigration. Horizontal homogeneity is assumed. The efm comprises linked sub-models which are described elsewhere for the trees [44], soil and litter ([45], chapter 5) and water ([46]; [45], chapter 6). Synopses of processes represented are given by Thornley [43], Thornley and Cannell [47] and Cannell et al. [48]. Recent developments of the model are: acclimation of photosynthesis to light, nitrogen, carbon dioxide and temperature [49]; components of respiration are more explicitly itemised [50]. See also the appendix of Thornley and Cannell [51] for diagrams of the efm.

Symbols relevant to the present application are listed in Appendix E. Important tree variables vis-à-vis the aphid sub-model are the leaf area per stem, A_{leaf} ($\mathrm{m}^2 \times \mathrm{stem}^{-1}$); stem density, n_{stems} (number of stems per m^2, a state variable of the efm); leaf area index, LAI [m^2 leaf $\times$ (m^2 ground)$^{-1}$]; these are given by:

$$A_{\mathrm{leaf}} = \sum_{i=j}^{4} A_{\mathrm{leaf}_j} \;\; (A_{\mathrm{leaf}_j} \text{ are efm state variables}). \tag{1a}$$

$$A_{\mathrm{leaf}}(t = 0) = 0.012\,\mathrm{m}^2\ \mathrm{stem}^{-1}. \tag{1b}$$

$$\frac{\mathrm{d}n_{\mathrm{stem}}}{\mathrm{d}t} = -O_{\mathrm{nstems,th}},\;\; n_{\mathrm{stems}}(t = 0) = 0.25\ \mathrm{stems\ m}^{-2}. \tag{1c}$$

$$L_{\mathrm{AI}} = n_{\mathrm{stems}} A_{\mathrm{leaf}},\;\; L_{\mathrm{AI}}(t = 0) = 0.003\,\mathrm{m}^2\ \mathrm{leaf}\ (\mathrm{m}^2\ \mathrm{ground})^{-1}. \tag{1d}$$

In Eq (1a), leaf area per stem, A_{leaf}, is represented by four age categories, 1–4 (Fig 1). The time $t = 0$ values each of these variables is 0.003 $\mathrm{m}^2 \times \mathrm{stem}^{-1}$. In the forest plantation simulations (Section 4.2) aphid infestation is assumed to occur at $t = 0$ [Eqs (4), (5) and (64)].

In the second equation, there is a single flux out of the n_{stems} state variable, $O_{\mathrm{nstems,th}}$ (stems $\times$ m^{-2} $\times$ d^{-1}) due to the management thinning regime (Section 4.2, first paragraph). Pruning is not included in our present simulations of aphid infestation; it is included in the model but is set to zero [Eq (14)].

Other important variables for the aphid implementation of the efm are the carbon (C) substrate and nitrogen (N) substrate concentrations in the foliage (leaf, le), C_{le} and N_{le}. Although the phloem C and N concentrations are not directly represented in the forest model, it is assumed that they can be obtained from the foliage (leaf) substrate concentrations, C_{le} and N_{le} [kg substrate C, substrate N (kg structural dry matter)$^{-1}$]

by simple multipliers:

$$C_{\rm phloem} = m_{\rm CS} C_{\rm le} \quad N_{\rm phloem} = m_{\rm NS} N_{\rm le} \tag{2a}$$

$$m_{\rm CS} = 5000, \quad m_{\rm NS} = 1000 \,\text{structural dry matter m}^{-2}. \tag{2b}$$

$$\text{At time}\, t = 0, \quad C_{\rm le} = 0.02\,\text{kg substrate C (kg structural dry matter)}^{-1}, \tag{2c}$$

$$N_{\rm le} = 0.01\,\text{kg substrate N (kg structural dry matter)}^{-1}, \tag{2d}$$

$$C_{\rm phloem} = 100 \;\text{kg substrate C (m}^3\text{ phloem sap)}^{-1}, \tag{2e}$$

$$N_{\rm phloem} = 10 \;\text{kg substrate C (m}^3\text{ phloem sap)}^{-1}. \tag{2f}$$

In Eq (2) the units of the phloem C and N concentrations, $C_{\rm phloem}$ and $N_{\rm phloem}$, are kg substrate C, substrate N (m^3 phloem sap)$^{-1}$ in the phloem. The values for the multipliers $m_{\rm CS}$ and $m_{\rm NS}$ are chosen so that reasonable values of phloem concentrations are obtained [52, 53]. For example, at time $t = 0$, $C_{\rm le} = 0.02$ kg substrate C (kg structural dry matter)$^{-1}$ converts to $C_{\rm phloem} = 100$ ($= 5000 \times 0.02$) kg substrate C (m^3 phloem)$^{-1}$ = 100 g substrate C (litre phloem)$^{-1}$ = 100/144 mol sucrose litre^{-1} = 0.7 molar sucrose in phloem (assuming that sucrose is $C_{12}H_{22}O_{11}$); $N_{\rm le} = 0.01$ kg substrate N (kg structural dry matter)$^{-1}$ converts to 10 ($= 1000 \times 0.01$) kg N $\times$ m^{-3} = 10 g N litre^{-1} = 10/14 = 0.7 molar glutamate (assuming that glutamic acid is $C_5H_9NO_4$).

The phloem C:N substrate ratio [kg C $\times$ (kg N)$^{-1}$] is [with Eq (2)]

$$r_{\rm CNphloem} = \frac{C_{\rm phloem}}{N_{\rm phloem}}, \quad r_{\rm CNphloem}(t = 0) = \frac{m_{\rm CS} C_{\rm le}(t = 0)}{m_{\rm NS} N_{\rm le}(t = 0)} \tag{3a}$$

$$= \frac{5000}{1000}\frac{0.02}{0.01} = 10 \;\;[\text{kg substrate C (kg substrate N)}^{-1}] \tag{3b}$$

Appendix D Aphid submodel

Contents

D.1 Apterous (wingless) adult aphids, $\boldsymbol{a_{\mathbf{pta}}}$ . . . 20
D.1.1 Inputs: apterous (wingless) adult aphids . . . 20
D.1.2 Outputs: apterous (wingless) adult aphids . . . 20
D.1.3 Differential equation: apterous (wingless) adult aphids . . . 23
D.2 Apterous aphid fecundity . . . 23
D.2.1 Apterous aphid fecundity as a function of temperature . . . 23
D.2.2 Apterous aphid fecundity as a function of phloem N concentration 24
D.3 Alate:apterous ratio in offspring from apterous adults . . . 25
D.3.1 Alate:apterous ratio as a function of temperature . . . 25
D.3.2 Alate:apterous ratio as a function of aphid total density . . . 26
D.3.3 Alate:apterous ratio as a function of nutritional status . . . 26
D.3.4 Total fecundity and the fractional fecundity of apterous adults . 27
D.3.5 Associated N and C fluxes . . . 27
D.4 Development rates of apterous aphids . . . 28
D.4.1 Survival . . . 29
D.5 Juvenile apterous (wingless) aphids . . . 29
D.5.1 Inputs: juvenile apterous (wingless) aphids . . . 29
D.5.2 Outputs: juvenile apterous (wingless) aphids . . . 30
D.5.3 Differential equations: juvenile apterous (wingless) aphids . . . 31
D.5.4 Nitrogen and carbon fluxes for the four apterous instar pools . . 31
D.6 Alate (winged) adult aphids, $\boldsymbol{a_{\mathbf{laa}}}$ . . . 32
D.6.1 Inputs: alate (winged) adult aphids . . . 32
D.6.2 Outputs: alate (winged) adult aphids . . . 32
D.6.3 Differential equation: alate (winged) adult aphids . . . 33
D.7 Alate aphid fecundity . . . 34
D.8 Alate:apterous ratio in offspring from alate adults . . . 35
D.8.1 Total fecundity and fractional fecundity of alate adults . . . 35
D.8.2 Associated N and C fluxes . . . 35
D.9 Development rates of alate aphids . . . 36
D.10 Juvenile alate (winged) aphids . . . 36
D.10.1 Inputs: juvenile alate (winged) aphids . . . 36
D.10.2 Outputs: juvenile alate (winged) aphids . . . 37
D.10.3 Differential equations: juvenile alate (winged) aphids . . . 37
D.10.4 Nitrogen and carbon fluxes for the four alate instar pools . . . 38
D.11 Aphid mortality, emigration and fecundity . . . 39
D.11.1 Mortality . . . 39
D.11.2 Emigration . . . 39
D.11.3 Fecundity . . . 40
D.11.4 Total aphid number per stem, $\boldsymbol{a_{\mathbf{ph}}}$ . . . 40
D.12 Aphid sub-model – C and N balances . . . 40
D.12.1 Carbon inputs: phloem, immigration . . . 41
D.12.2 Carbon outputs: respiration, honeydew, mortality, emigration, pruning, thinning . . . 41
D.12.3 Carbon balance . . . 42
D.12.4 Nitrogen inputs: phloem, immigration . . . 42
D.12.5 Nitrogen outputs: mortality, emigration, pruning, thinning . . . 43
D.12.6 N balance . . . 43
D.13 Soil sub-model inputs from: mortality, pruning and thinning . . . 44

D.14 Aphid C and N at the end of a rotation 44

The aphid submodel is shown schematically in Fig 2. The model has 10 state variables. There are two morphs of the aphid: alate (winged; ala) and aperterous (wingless, apt). Each morph has four juvenile stages called instars, and an adult stage. All aphid state variables have units of numbers of aphids per stem. All notation is summarized in Appendix E.

We define the total number of alate instars (a_{lai}), alate adults (a_{laa}), the total alates (a_{la}), the same for apterous aphids (a_{pti}, a_{pta}, a_{pt}) and the total number of aphids (a_{ph}) as:

$$a_{\mathrm{lai}} = a_{\mathrm{la1}} + a_{\mathrm{la2}} + a_{\mathrm{la3}} + a_{\mathrm{la4}}, \tag{4a}$$

$$a_{\mathrm{la}} = a_{\mathrm{lai}} + a_{\mathrm{laa}}, \tag{4b}$$

$$a_{\mathrm{pti}} = a_{\mathrm{pt1}} + a_{\mathrm{pt2}} + a_{\mathrm{pt3}} + a_{\mathrm{pt4}}, \tag{4c}$$

$$a_{\mathrm{pt}} = a_{\mathrm{pti}} + a_{\mathrm{pta}}, \tag{4d}$$

$$a_{\mathrm{ph}} = a_{\mathrm{al}} + a_{\mathrm{pt}}. \tag{4e}$$

At $t = 0$, $a_{\mathrm{ph}} = 10$ aphids per stem.

The spruce aphid does not colonize and feed on the current year's foliage [54]. From the four foliage categories in the efm, only the last three are used here for the calculation of aphid densities on foliage ($A_{\mathrm{leaf,aph}}$). Dividing by the colonized leaf area per stem, $A_{\mathrm{leaf,aph}}$ (m^2 stem^{-1}), aphid density (ρ) per unit area of foliage (aphids m^{-2}) are:

$$A_{\mathrm{leaf,aph}} = A_{\mathrm{leaf,2}} + A_{\mathrm{leaf,3}} + A_{\mathrm{leaf,4}}, \tag{5a}$$

$$\rho_{\mathrm{aph}} = \frac{a_{\mathrm{ph}}}{A_{\mathrm{leaf,aph}}}. \tag{5b}$$

At $t = 0$, $A_{\mathrm{leaf,aph}} = 0.009$ (m^2 stem^{-1}), and $\rho = 10/0.009 = 1111.1$ aphids (m^2 leaf)$^{-1}$. The $t = 0$ values are for young seedlings [Eq (1)] and the 'standard' aphid infestation, Eq (64) and for the plantation defined in Section 4.2 above. Note that an aphid density of 1000 aphids (m^2 leaf)$^{-1}$ corresponds to a distance between aphids of about 3 cm.

Using the aphid state variables, the C and N contents of the aphids per stem are (units: kg aphid C, N stem^{-1}):

$$C_{\mathrm{ala}} = a_{\mathrm{la1}} M_{\mathrm{Cala1}} + a_{\mathrm{la2}} M_{\mathrm{Cala2}} + a_{\mathrm{la3}} M_{\mathrm{Cala3}} + a_{\mathrm{la4}} M_{\mathrm{Cala4}} + a_{\mathrm{laa}} M_{\mathrm{Calaa}}, \tag{6a}$$

$$N_{\mathrm{ala}} = a_{\mathrm{la1}} M_{\mathrm{Nala1}} + a_{\mathrm{la2}} M_{\mathrm{Nala2}} + a_{\mathrm{la3}} M_{\mathrm{Nala3}} + a_{\mathrm{la4}} M_{\mathrm{Nala4}} + a_{\mathrm{laa}} M_{\mathrm{Nalaa}}, \tag{6b}$$

$$C_{\mathrm{apt}} = a_{\mathrm{pt1}} M_{\mathrm{Cala1}} + a_{\mathrm{pt2}} M_{\mathrm{Cala2}} + a_{\mathrm{pt3}} M_{\mathrm{Cala3}} + a_{\mathrm{pt4}} M_{\mathrm{Cala4}} + a_{\mathrm{pta}} M_{\mathrm{Calaa}}, \tag{6c}$$

$$N_{\mathrm{apt}} = a_{\mathrm{pt1}} M_{\mathrm{Nala1}} + a_{\mathrm{pt2}} M_{\mathrm{Nala2}} + a_{\mathrm{pt3}} M_{\mathrm{Nala3}} + a_{\mathrm{pt4}} M_{\mathrm{Nala4}} + a_{\mathrm{pta}} M_{\mathrm{Nalaa}}, \tag{6d}$$

$$C_{\mathrm{aph}} = C_{\mathrm{ala}} + C_{\mathrm{apt}}, \tag{6e}$$

$$N_{\mathrm{aph}} = N_{\mathrm{ala}} + N_{\mathrm{apt}}. \tag{6f}$$

The C and N contents of each morphological aphid form (Fig 2) are constants, unaffected by growth temperature or nutritional status and are (units: kg C, N

aphid^{-1}):

$$M_{\text{Cala1}} = M_{\text{Nala1}} = 1 \times 10^{-6}, \tag{7a}$$
$$M_{\text{Cala2}} = M_{\text{Nala2}} = 2 \times 10^{-6}, \tag{7b}$$
$$M_{\text{Cala3}} = M_{\text{Nala3}} = 3 \times 10^{-6}, \tag{7c}$$
$$M_{\text{Cala4}} = M_{\text{Nala4}} = 4 \times 10^{-6}, \tag{7d}$$
$$M_{\text{Calaa}} = M_{\text{Nalaa}} = 5 \times 10^{-6}, \tag{7e}$$
$$M_{\text{Capt1}} = M_{\text{Napt1}} = 1 \times 10^{-6}, \tag{7f}$$
$$M_{\text{Capt2}} = M_{\text{Napt2}} = 2 \times 10^{-6}, \tag{7g}$$
$$M_{\text{Capt3}} = M_{\text{Napt3}} = 3 \times 10^{-6}, \tag{7h}$$
$$M_{\text{Capt4}} = M_{\text{Napt4}} = 4 \times 10^{-6}, \tag{7i}$$
$$M_{\text{Capta}} = M_{\text{Napta}} = 5 \times 10^{-6}. \tag{7j}$$

These estimates were made to enable the model to be completed, although we have been unable to find measurements of these quantities. The values are not critical for the range of behaviour exhibited by the model (See chapter 4 of Dixon [55] and particularly figure 4.1 on p. 59; note that 1×10^{-6} kg = 1 mg = 1000 μg).

D.1 Apterous (wingless) adult aphids, a_{pta}

D.1.1 Inputs: apterous (wingless) adult aphids

There is a single input, from the developmental output flux of the fourth apterous instar (a_{pt4}, Fig 2), $O_{\text{apt4}\to\text{a}}$, calculated in Eq (42) (the output is assumed to become an input without loss). The input, I_{apta}, requires C and N fluxes, of $I_{\text{Capt4}\to\text{a}}$ and $I_{\text{Napt4}\to\text{a}}$:

$$I_{\text{apta}} = O_{\text{apt4}\to\text{a}}, \quad \text{and} \tag{8a}$$
$$I_{\text{Capt4}\to\text{a}} = I_{\text{apta}} M_{\text{Capta}}, \quad I_{\text{Napt4}\to\text{a}} = I_{\text{apta}} M_{\text{Napta}}. \tag{8b}$$

Units of the first of Eq (8) are aphids stem^{-1} d^{-1} and of the other two are kg aphid C, N stem^{-1} d^{-1}. See Eq (7) for the C, N contents of apterous adults.

D.1.2 Outputs: apterous (wingless) adult aphids

It is assumed that there is no flux of aphids to surface litter accompanying foliage litter flux. Thinning, as applied in 'plantation' mode [Section 4.2, Eq (1)] — i.e. removal of whole trees (stems), does not affect aphid state variables which are aphids per stem (although aphid number per unit ground area is affected). This leaves two outputs: mortality (dependent on temperature and nutrition) and pruning (removing branches including foliage).

Mortality. Our approach to aphid mortality is mostly guess work, as there is little guidance on the topic which is relevant to a mechanistic modelling exercise where ordinary differential equations are the basic representational tool. Some authors prefer to talk of 'survival' (e.g. Duffy et al., 2017 [20]; see their figure 3), but survival then depends on a development rate (Section D.4) as well as a mortality rate. Dixon ([55], p. 165, see their figure 7.25) presents mortality rate as related to relative growth rate, without specifying units, rather than relating mortality rate to forest sub-model variables (such as phloem N) and driving variables (such as air temperature). We ignore

any possible influence of predators on mortality. There is some evidence from other mechanistic aphid modelling that considering predation does not fundamentally effect the conclusions regarding the impacts of climatic change [8].

It is assumed that aphid mortality, $k_{\mathrm{aph,mort}}$, depends on air temperature, T_{air} and phloem N, N_{phloem}; the specific rates (d^{-1}) are calculated independently [Eqs (9) and (10)] and then combined in Eq (11). Adult apterous mortality rate is then $O_{\mathrm{apta\to mort}}$ (aphids stem^{-1} d^{-1}), given by Eq (12).

The specific air-temperature-dependent mortality rate, $k_{\mathrm{aph,Tmort}}$ (d^{-1}), is given by a skewed inverted parabola (Fig D.1):

$$k_{\mathrm{aph,Tmort}} = \left[k_{\mathrm{aph,Tmort,opt}} + \left(k_{\mathrm{aph,Tmort,0}} - k_{\mathrm{aph,Tmort,opt}}\right) \times \frac{\left(T_{\mathrm{air}} - T_{\mathrm{aph,mort,opt}}\right)^2}{\left(T_{\mathrm{aph,mort,0}} - T_{\mathrm{aph,mort,opt}}\right)^2} \right] \times \left[1 + c_{\mathrm{aph,mort,skew}} \left(\frac{T_{\mathrm{air}} - T_{\mathrm{aph,mort,opt}}}{T_{\mathrm{aph,mort,opt}} - T_{\mathrm{aph,mort,0}}} \right) \right]. \tag{9a}$$

$$T_{\mathrm{aph,mort,0}} = 0, \ \ T_{\mathrm{aph,mort,opt}} = 15^{\circ}\mathrm{C}; \tag{9b}$$

$$k_{\mathrm{aph,Tmort,0}} = 0.04, \ \ k_{\mathrm{aph,Tmort,opt}} = 0\,\mathrm{d}^{-1}, \ \ c_{\mathrm{aph,mort,skew}} = 0.2 \tag{9c}$$

If air temperature T_{air} equals the optimum temperature for minimum aphid mortality, $T_{\mathrm{aph,mort,opt}}$, then mortality rate $k_{\mathrm{aph,Tmort}} = k_{\mathrm{aph,Tmort,opt}}$ is zero. If temperature $T_{\mathrm{air}} = T_{\mathrm{aph,mort,0}}$ (the second reference point, taken here to be 0 °C), then $k_{\mathrm{aph,Tmort}} = k_{\mathrm{aph,Tmort,0}} = 4\%\ \mathrm{d}^{-1}$. The second term in square brackets skews the response about the optimum temperature (here 15 °C), according to the value of the skewness parameter $c_{\mathrm{aph,mort,skew}}$. With the value given, if $T_{\mathrm{air}} = T_{\mathrm{aph,mort,0}}$, then the mortality rate is decreased by 20%. The temperature response of mortality rate is drawn in Fig D.1 for several values of the skewness parameter.

The most significant part of the response drawn in Fig D.1 is the increase in mortality as the temperature is lowered. This causes the aphid infestation to become less severe or even zero as the temperature is lowered (Fig 4).

Nutrition-dependent mortality rate, $k_{\mathrm{aph,Nmort}}$ (d^{-1}), is described by a 'switch-off' sigmoidal response (e.g. [43], equation 4.61, figure 4.9, pp. 109-111), to phloem N concentration, N_{phloem} [Eq (2), Fig D.2], given by

$$k_{\mathrm{aph,Nmort}} = k_{\mathrm{aph,Nmort,max}} \frac{\left(K_{\mathrm{Naph,mort}}\right)^{q_{\mathrm{aph,Nmort}}}}{\left(K_{\mathrm{Naph,mort}}\right)^{q_{\mathrm{aph,Nmort}}} + \left(N_{\mathrm{phloem}}\right)^{q_{\mathrm{aph,Nmort}}}}. \tag{10a}$$

$$k_{\mathrm{aph,Nmort,max}} = 0.1\,\mathrm{d}^{-1}, \ \ K_{\mathrm{Naph,mort}} = 1\mathrm{kg\,N\,m}^{-3}, \ \ q_{\mathrm{aph,Nmort}} = 2. \tag{10b}$$

The maximum mortality rate, $k_{\mathrm{aph,Nmort,max}}$, if there is no food ($N_{\mathrm{phloem}} = 0$) is 10% per day. The half-maximal mortality rate (5% per day) occurs when $N_{\mathrm{phloem}} = K_{\mathrm{Naph,mort}} = 1$ kg N m^{-3} = 1/14 mol N. Note that, from Eq (2), this corresponds to a foliage substrate N concentration of $N_{\mathrm{le}} = 0.01$ kg substrate N (kg structural dry matter)$^{-1}$. The nutritionally-dependent mortality rate approaches zero at high values of N_{phloem}. The steepness of the response depends on $q_{\mathrm{aph,Nmort}}$, here assigned the value 2. The response is drawn in Fig D.2 for four values of $q_{\mathrm{aph,Nmort}}$.

Fig D.1. Aphid mortality rate as affected by temperature. Eq (9) is plotted for three values of the skewness parameter $c_{\mathrm{aph,mort,skew}}$ as given. The default value used in the simulations is 0.2, shown by the dark line. Other parameters are as in Eq (9).

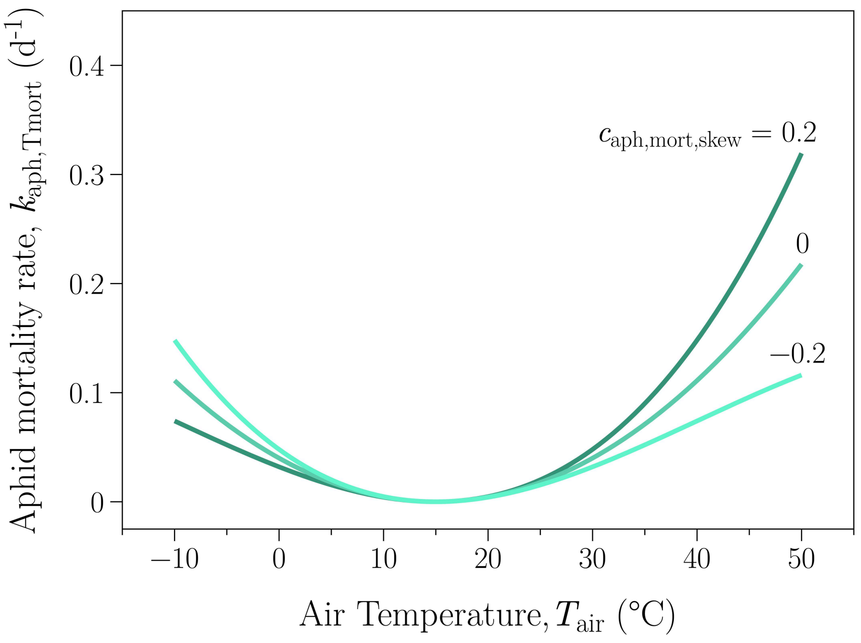

Fig D.2. Nutritional response of aphid mortality. Aphid mortality rate as affected by N nutrition, in particular, N concentration in the phloem, N_{phloem} [Eq (2)]. Eq (10) is plotted for four values of the steepness parameter, $q_{\text{aph,Nmort}}$, as given. The default value used in the simulations is $q_{\text{aph,Nmort}} = 2$.

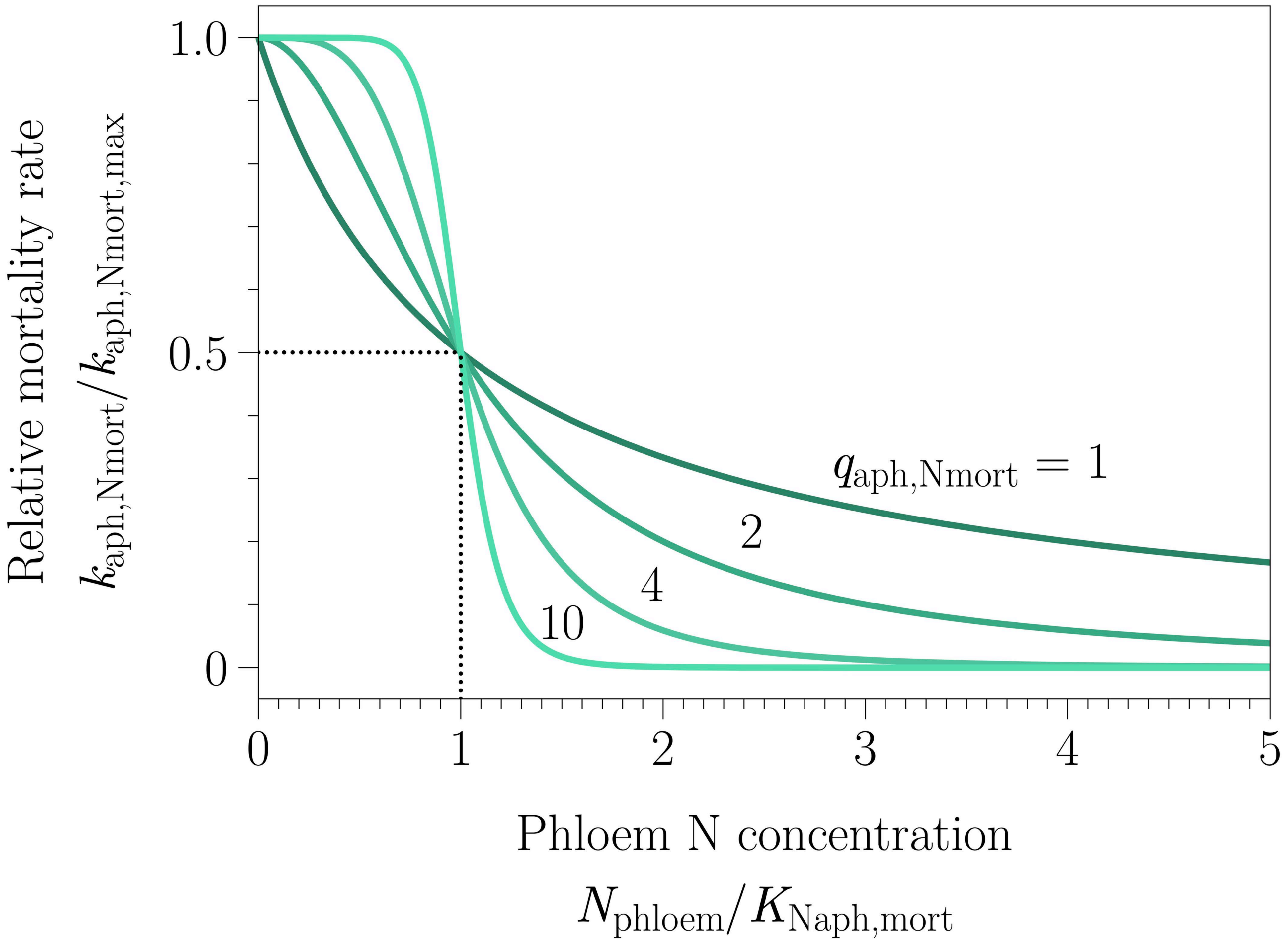

It is assumed that the overall mortality rate (d^{-1}), $k_{aph,mort}$, is given by summing the temperature-dependent and the nutritionally-dependent mortality rates [Eqs (9) and (10); assuming aphid temperature = air temperature, T_{air} (°C)]. Therefore:

$$k_{aph,mort} = k_{aph,Tmort} + k_{aph,Nmort} \tag{11}$$

The mortality rate varies between 2% and 6% per day, depending on temperature and N nutrition (Fig D.3B).

We have mentioned above (Section D.1.2), our difficulties with mortality rate. We use the same mortality rate, $k_{aph,mort}$ [Eq (11)], for all the aphid state variables (Fig 2), although this could be easily relaxed. In Fig D.3, the components of mortality are examined. Mortality rate can be calculated in the absence of aphids, although the presence and activity of aphids depresses phloem N (Fig D.3D) and this increases N phloem-dependent mortality [$k_{aph,Nmort}$, Fig D.3C, Fig D.2, Eq (10)]. Temperature-dependent mortality, $k_{aph,Tmort}$, is the same whether aphids are present or not [Fig D.3C, Eq (9)]. Fig D.3A shows the effect of aphids on leaf area index [Eq (1)] at a constant stem density, nstems, [Eq (1)]. Later, after considering developmental rates (Section D.4), we examine survival in Section D.4.1 and Fig D.8.

With Eq (11) for specific aphid mortality rate, $k_{aph,mort}$ (d^{-1}), output from the apterous adult pool, a_{pta}, to mortality is (aphids stem^{-1} d^{-1})

$$O_{apta\rightarrow mort} = k_{aph,mort} a_{pta}. \tag{12}$$

There are output (O) C and N fluxes associated with this aphid flux (using an obvious notation):

$$O_{Capta\rightarrow mort} = O_{apta\rightarrow mort} M_{Capta}, \quad O_{Napta\rightarrow mort} = O_{apta\rightarrow mort} M_{Napta}. \tag{13}$$

Units are kg C, N stem^{-1} d^{-1}. Aphid C and N contents are given in Eq (7). These fluxes are input to the soil surface litter pools [Eqs (108) and (118)].

Pruning. The pruning flux of apterous adults is $O_{apta\rightarrow prune}$ (aphids stem^{-1} d^{-1}). Foliage pruning, if applied, occurs at a rate of $k_{le\rightarrow prune}$ (d^{-1}). This gives rise to outputs

Fig D.3. Aphid mortality.. Contributions to aphid mortality are shown over 12 months without aphids being present (dash-dot lines) and with aphid infection at t = 0 (solid lines) of 10 adult alates per stem [Eq (64)]. At t = 0 d the seedling plants have an LAI of 0.003 and a stem density 0.25 stems m^{-2} [See also Eqs (1), (4) and (5)]. **A**, leaf area index, L_{AI} [Eq (1)]. **B**, total aphids, a_{ph} [Eq (4)]; aphid mortality rate, $k_{aph,mort}$ [Eq (11)]. **C**, temperature-dependent mortality, $k_{aph,Tmort}$ [Eq (9)]; nitrogen-dependent mortality, $k_{aph,Nmort}$ [Eq(10)]. **D**, Air temperature, T_{air} (Fig 3), the determinant of temperature-dependent mortality in **C**; phloem N [Eq (2)], the determinant of nutrition-dependent mortality in **C**, phloem N is decreased by aphid infestation (solid line).

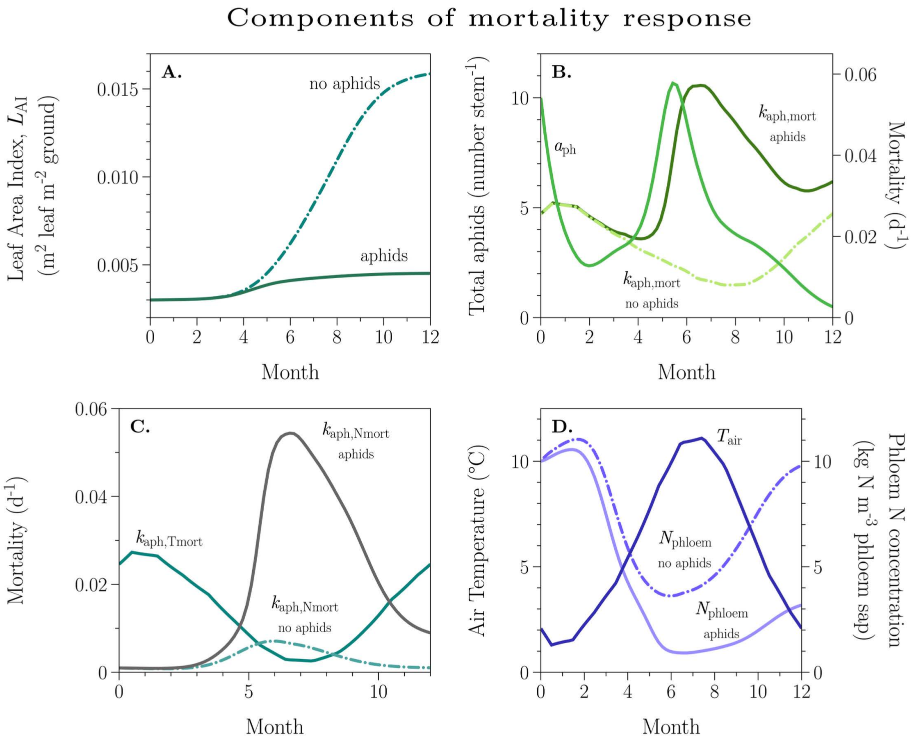

(losses) of aphids and associated fluxes of C and N (kg aphid C, N stem^{-1} d^{-1}), of

$$O_{\text{apta}\to\text{prune}} = k_{\text{le}\to\text{prune}} a_{\text{apta}} \tag{14a}$$

$$O_{\text{Capta}\to\text{prune}} = O_{\text{apta}\to\text{prune}} M_{\text{Capta}}, \quad O_{\text{Nata}\to\text{prune}} = O_{\text{apta}\to\text{prune}} M_{\text{Napta}}, \tag{14b}$$

$$k_{\text{le}\to\text{prune}} = 0\,\text{d}^{-1}. \tag{14c}$$

The foliage pruning constant, $k_{\text{le}\to\text{prune}}$, is not used in the simulations presented here and it is set to zero. This aphid output flux is added to the mortality flux in Eq (15).

The total output of adult apterous aphids, O_{apta}, is obtained by adding the contributions from mortality [Eq (12)] and pruning [Eq (14)]:

$$O_{\text{apta}} = O_{\text{apta}\to\text{mort}} + O_{\text{apta}\to\text{prune}}. \tag{15}$$

D.1.3 Differential equation: apterous (wingless) adult aphids

The differential equation (apterous aphids stem^{-1} d^{-1}) and initial value for the state variable, a_{pta}, for the apterous adult aphids is:

$$\frac{da_{\text{pta}}}{dt} = I_{\text{apta}} - O_{\text{apta}}, \tag{16a}$$

$$a_{\text{pta}}(t = 0) = 0 \text{ apterous adults stem}^{-1}. \tag{16b}$$

The input and output terms are given by Eqs (43) and (15).

D.2 Apterous aphid fecundity

Adult aphids give birth to offspring or nymphs. The rate at which this occurs is influenced by temperature and phloem N concentration. The N ingested is entirely converted to wingless (apt) and winged (ala) 1st instar nymphs, a_{pt1} and a_{la1} (Fig 2). These can have different N contents [Eq (7)]. Account must be taken of this before total and fractional fecundities can be calculated [Section D.3.4; Eq (29)].

D.2.1 Apterous aphid fecundity as a function of temperature

A general and easily adjustable temperature response function, $f(T)$, used for many plant and soil biological processes is ([43], pp. 105–106)

$$f(T) = \frac{(T - T_0)^{q_1}(T_0' - T)^{q_2}}{(T_{\text{ref}} - T_0)(T_0' - T_{\text{ref}})^{q_2}} \text{ for } T_0 < T < T_0', \text{ else } 0. \tag{17a}$$

$$q_1 = 2,\ q_2 = 1,\ T_0 = 0\,^\circ\text{C},\ T_{\text{ref}} = 20\,^\circ\text{C}, T_0' = 45\,^\circ\text{C}, \tag{17b}$$

$$T_{max} = \frac{q_1 T_0' + q_2 T_0}{q_1 + q_2} = 30\,^\circ\text{C}. \tag{17c}$$

Here default values of the five parameters are given; the shape of the default response is illustrated by the continuous lines in Fig D.4A–D. The default is a cubic. $f(T)$ is only non zero between temperatures T_0 and T_0'. Fig D.4A shows the effect of varying the parameter q_1. The response is initially sigmoidal if $q_1 > 1$. In Fig D.4B, q_2 is varied. It can be seen that the steepness of the high-temperature decrease is highly dependent on q_2 for $q_2 < 1$, although the early part of the curve is much less affected by q_2. In Fig D.4C, T_0 is varied and in Fig D.4D, T_0'. T_{ref} is a reference temperature: $f(T) = 1$ at $T = T_{\text{ref}} = 20$ °C. The five parameters define: the zero points of $f(T)$ (T_0 and T_0'); the shape of the curve in the neighbourhood of the zero points (q_1 and q_2); and the

Fig D.4. General biological temperature function. General biological temperature function, Eq (17), with five parameters: q_1, q_2, T_0, T_0' and T_{ref}, of which four are varied in the figure. This is applied in the efm and the aphid sub-model for various processes. It is drawn, in A for three values of parameter q_1, in B for three values of q_2, in C for three values of T_0 and in D for three values of T_0'. Otherwise, the parameters have the values given in Eq (17). Commonly used default values are shown by the continuous heavy lines.

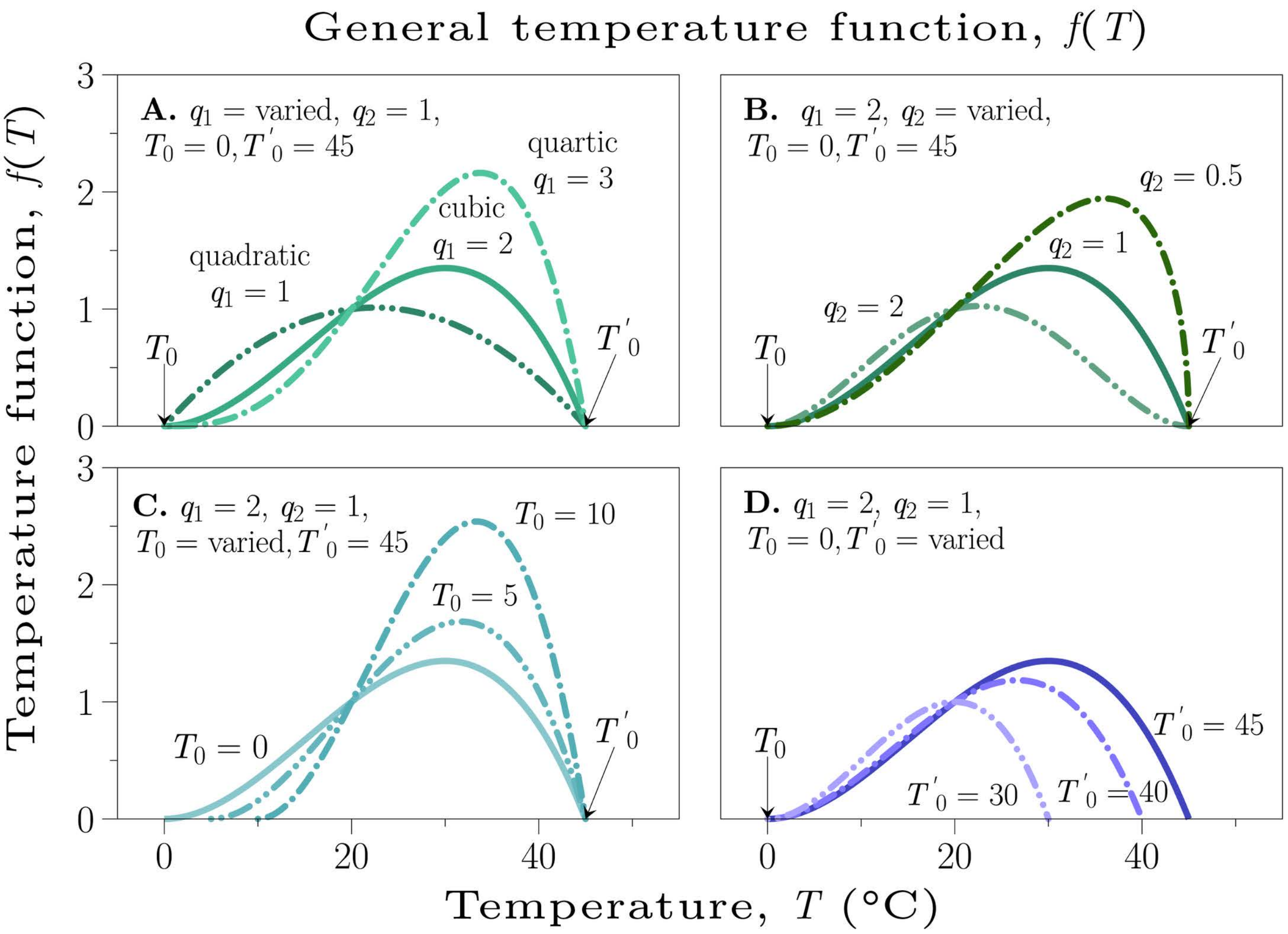

reference temperature, T_{ref}. An equation for the temperature where $f(T)$ is maximum is given, T_{max}; the temperature of any point of inflexion can be derived.

Taking $T = T_{\mathrm{air}}$ and with the default parameters given, Eq (17) is used to modify many above-ground (shoot) plant processes (where it is reasonable to take shoot temperature equal to air temperature, T_{air}), as well as the temperature dependence assumed for some aphid processes, e.g. emigration [Eq (61)].

The effect of temperature on aphid fecundity, $f_{\mathrm{Taph,fec}}$, is obtained by using air temperature, T_{air} and the standard temperature function in Eq (17) and Fig D.4, but with two different temperature parameters (T_0, T_0'):

$$f_{\mathrm{Taph,fec}}(T = T_{\mathrm{air}}) = f(T_{\mathrm{air}}, T_0 = T_{\mathrm{0aph,fec}} = 3, T_0' = T'_{\mathrm{0,aph,fec}} = 30\ {}^\circ\mathrm{C}). \tag{18}$$

This is drawn in Fig D.5A. It can be compared with Duffy et al.'s [20] figure 2, who make use of data from Dean [56], although our calculations of total and fractional fecundities are completed below [Section D.3.4, Eq (29)].

D.2.2 Apterous aphid fecundity as a function of phloem N concentration

Aphids are assumed to have a maximum volume intake of phloem sap at a given temperature, $v_{\mathrm{apta,max}}$ (m^3 aphid^{-1} d^{-1}), obtained by $f_{\mathrm{Taph,fec}}(T_{\mathrm{air}})$ of Eq (18) (Fig D.5A) multiplied by the maximum volume intake at the reference temperature of 20 °C:

$$v_{\mathrm{apta,max}} = (f_{\mathrm{Taph,fec}})(v_{\mathrm{apta,max20}}), \quad v_{\mathrm{apta,max20}} = 0.2 \times 10^{-6}\ \mathrm{m}^3\ \mathrm{aphid}^{-1}\ \mathrm{d}^{-1}. \tag{19}$$

The 20 °C value, $v_{\mathrm{apta,max20}}$, corresponds to 0.2 ml aphid^{-1} d^{-1}. The maximum phloem-volume limited N intake per aphid is (units are kg N aphid^{-1} d^{-1}) (Fig D.5C):

$$I_{\mathrm{Nphloem,vapta,max}} = (v_{\mathrm{apta,max}})(N_{\mathrm{phloem}}). \tag{20}$$

N_{phloem} is given by Eq (2) [kg N substrate in phloem (m^3 phloem sap)$^{-1}$]. This may exceed the N requirement of the aphid, defined as the maximum amount of N which can be processed (p) at the ambient temperature (T_{air}), calculated by

$$I_{\mathrm{Npapta,max}} = f_{\mathrm{Taph,fec}}(T_{\mathrm{air}}) I_{\mathrm{Npapta,max20}}; \quad I_{\mathrm{Npapta,max20}} = 2 \times 10^{-6}\ \mathrm{kg\ N\ aphid}^{-1}\ \mathrm{d}^{-1}. \tag{21}$$

$f_{\mathrm{Taph,fec}}(T_{\mathrm{air}})$ is given in Eq (18). $I_{\mathrm{Npapta,max20}}$ is the maximum amount of N that can be processed (p) at the reference temperature of 20 °C. $I_{\mathrm{Npapta,max}}$ is illustrated in Fig D.5C. Note that here we have used the same temperature dependence, $f_{\mathrm{Taph,fec}}$, for the process-dependence [Eq (21)] as for the volume limitation [Eq (19)].

The actual amount of N ingested per aphid per d is the least of the volume-intake-limited N-intake [Eq (20)] or the processing-limited N-intake [Eq (21)] (kg N aphid^{-1} d^{-1}):

$$I_{\mathrm{phloem \to apta,aphid}} = \min\{I_{\mathrm{Nphloem,vapta,max}}, I_{\mathrm{Npapta,max}}\}. \tag{22}$$

Neither of the two arguments is affected by the presence of aphids—e.g. which will depress N_{phloem} (Fig D.5B). In fact the processing-limited maximum is about eight-times as large as the volume-limited maximum (Fig D.5C), so the volume-limitation wins the argument in Eq (22).

Dividing by N_{phloem} [Eq (2)], Fig D.5B] (kg N m^{-3}), which is affected by the aphid infestation, the actual volume of phloem sap ingested per apterous adult aphid is (m^3 aphid^{-1} d^{-1}):

$$v_{\text{phloem}\to\text{apta}} = \frac{I_{\text{Nphloem}\to\text{apta,aphid}}}{N_{\text{phloem}}}. \tag{23}$$

This is illustrated in Fig D.5B and Fig D.5D.

The C intake per aphid (kg C aphid^{-1} d^{-1}) and the N and C intakes per stem (kg N, C stem^{-1} d^{-1}) are respectively:

$$I_{\text{Cphloem}\to\text{apta,aphid}} = v_{\text{phloem}\to\text{apta}} C_{\text{phloem}}, \tag{24a}$$
$$I_{\text{Nphloem}\to\text{apta}} = a_{\text{pta}} I_{\text{Nphloem}\to\text{apta,aphid}}, \tag{24b}$$
$$I_{\text{Cphloem}\to\text{apta}} = a_{\text{pta}} I_{\text{Cphloem}\to\text{apta,aphid}} \tag{24c}$$

The right side quantities are given in Eqs (23), (2), (16), (22) and the first of (24).

D.3 Alate:apterous ratio in offspring from apterous adults

The N ingested is assumed to be completely converted to wingless (apt) and winged (ala) first instar nymphs, in pools a_{pt1} and a_{la1} (Fig 2). Because these two forms can have different masses (N contents) [Eq (7)], the fractions of each type must first be calculated before total fecundity can be determined (Section D.3.4). It is assumed that the ratio is affected by three factors: temperature, total aphid density and nutritional status [57]. It is assumed that the three factors operate multiplicatively and for each factor x, the fraction of the offspring of apterous female adults which are alate (winged), $f_{\text{x,apt}\to\text{ala}}$, is calculated. The possible effects of day length are ignored (but see figure 6.8, p. 109 in [55]). Duffy et al. ([20], equation 4) assume the percentage of nymphs which are alates increases with aphid density and also growth stage of the cereal crop.

D.3.1 Alate:apterous ratio as a function of temperature

Higher air temperatures, T_{air} (°C), generally give more alates ([55], page 109). A positive (switch-on) sigmoidal dependence on air temperature (T_{air}) is assumed ([43], pp. 109 - 110), with:

$$f_{\text{Tapta}\to\text{ala1}} = \frac{T_{\text{air}}^{q_{\text{Tapt}}}}{T_{\text{air}}^{q_{\text{Tapt}}} + K_{\text{Tapt}}}, \quad q_{\text{Tapt}} = 2, K_{\text{Tapt}} = 10\ ^\circ\text{C}. \tag{25}$$

Fig D.5. Elements determining fecundity. Factors contributing to fecundity are shown as they occur in an Eskdalemuir environment (Section 4.1), for the first year of growth of typical spruce plantation (Section 4.2) infected with ten alate adults at time zero [Eq (64)]. A, air temperature, T_{air}, and its effect on fecundity, $f_{\mathrm{Taph,fec}}$ [Eq (18)]. B, phloem N concentration, N_{phloem} [Eq (2)] is illustrated without and with aphid infection which lowers N_{phloem} levels and thereby increases the actual volume of phloem sap ingested, $v_{\mathrm{phloem}\rightarrow\mathrm{apta}}$ [Eq (23)]. C, alternatives for the N ingested per aphid per day: (i) process-limited (p) N intake, $I_{\mathrm{Npapta,max}}$ [Eq (21)]; (ii) phloem-volume-limited (v) N intake, $I_{\mathrm{Nphloem,vapta,max}}$ [Eq (20)]. D, actual phloem volume ingested per aphid per day, $v_{\mathrm{phloem}\rightarrow\mathrm{apta}}$ [Eq (23)].

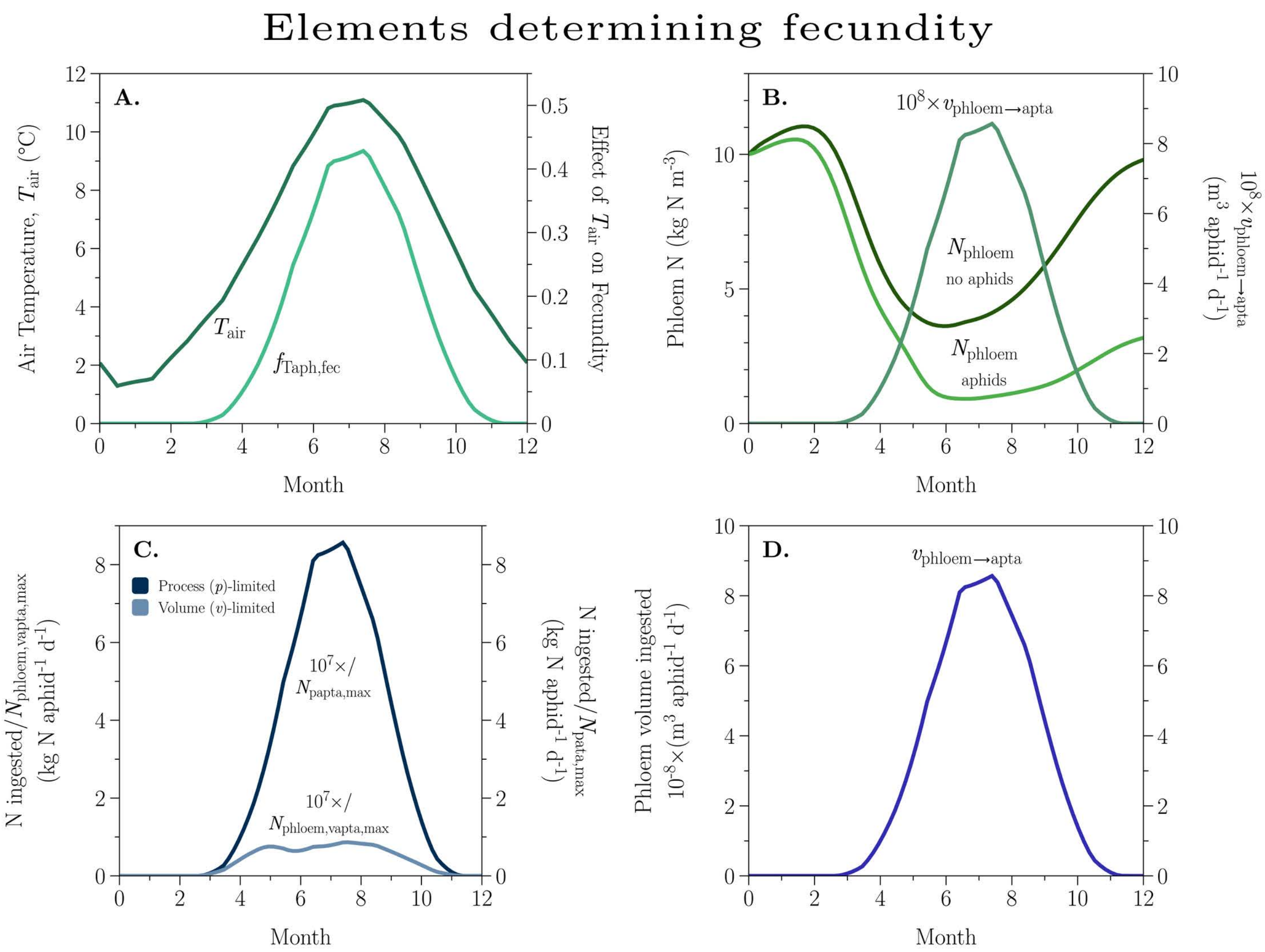

This is drawn in Fig D.6. K_{Tapt} is the half-maximal response temperature. q_{Tapt} determines the steepness of the response; $q_{\text{Tapt}} = 1$ gives the familiar Michaelis-Menten response.

D.3.2 Alate:apterous ratio as a function of aphid total density

This is ρ_{aph} [aphids (m^2 leaf area)$^{-1}$], Eq (5). Higher aphid densities cause more of the apterous offspring to be alates (winged). As in Eq (25) and Fig D.6 above for relative air temperature, a positive sigmoidal dependence on aphid density, ρ_{aph}, is assumed:

$$f_{\rho\text{apta}\to\text{ala1}} = \frac{(\rho_{\text{aph}})^{q_{\rho\text{apt}}}}{(\rho_{\text{aph}})^{q_{\rho\text{apt}}} + K_{\rho\text{aph,apt}}}, q_{\rho\text{apt}} = 2, K_{\rho\text{aph,apt}} = 1000 \text{ aphids (m}^2\text{ leaf area)}^{-1} \tag{26}$$

The half-maximum density point is where $\rho_{\text{aph}} = K_{\rho\text{aph,apt}}$; the value given is equivalent to aphids being on a square grid 3 cm apart and is of the same order as the annual peak in ρ_{aph}.

D.3.3 Alate:apterous ratio as a function of nutritional status

In this case, good nutritional status [a high value of N_{phloem}, Eq (2)] gives few alates and the alate fraction approaches zero for high values of N_{phloem}. Low nutritional status and a low N_{phloem} causes the alate fraction $f_{\text{Napta}\to\text{ala1}}$ to approach unity. An expression similar to that used in Fig D.2 for aphid mortality is employed [a 'switch-off' sigmoid rather than the 'switch-on' sigmoid used in Eq (26) and Eq (25); Fig D.6] ([43], figure 4.9, p. 111):

$$f_{\text{Napta}\to\text{ala1}} = \frac{(K_{\text{Napt}})^{q_{\text{Napt}}}}{(K_{\text{Napt}})^{q_{\text{Napt}}} + N_{\text{phloem}}}, \quad q_{\text{Napt}} = 2, K_{\text{Napt}} \text{ kg N m}^{-3}. \tag{27}$$

The half-maximal phloem N concentration of $K_{\text{Napt}} = 4$ kg N m^{-3} is equivalent to a foliage N substrate concentration of $N_{\text{le}} = 0.04$ kg N substrate (kg structural dry matter)$^{-1}$ [Eq (2)].

Combining these three factors multiplicatively [Eqs (25), (26) and (27)], therefore the fraction of offspring from apterous adults which are alate (winged) and are apterous are given by:

$$f_{\text{apta}\to\text{ala1}} = f_{\text{Tapta}\to\text{ala1}} f_{\rho\text{apta}\to\text{ala1}} f_{\text{Napta}\to\text{ala1}} \tag{28a}$$

$$f_{\text{apta}\to\text{apt1}} = 1 - f_{\text{apta}\to\text{ala1}} \tag{28b}$$

These three components and the outcome for $f_{\text{apta}\to\text{ala1}}$ are illustrated in Fig D.7.

The relative contributions to the fraction of apterous offspring assigned to the alate pathway are shown in Fig D.7 [Fig 2, Eq (28)]. In Fig D.7A, with dependence on air temperature, T_{air}, this fraction, $f_{\text{Tapta}\to\text{ala1}}$ [Eq (25)] is not affected by the presence of aphids. In Fig D.7B, showing dependence on aphid density, ρ_{aph}, the fraction is zero in the absence of aphids [Eq (26), with $\rho_{\text{aph}} = 0$]. The dependence on phloem N, N_{phloem}, is illustrated in Fig D.7C [Eq (27)]. Because phloem N is consumed by aphids, their presence lowers N_{phloem}, giving the lower continuous line for N_{phloem}; this also raises the alate fraction of apterous offspring (upper dashed line). Last, in Fig D.7D, the three fractions shown in Fig D.7A–C are multiplied in Eq (28) to give the final allocation fraction, $f_{\text{apta}\to\text{ala1}}$.

Fig D.6. Alate fraction of apterous offspring as affected by temperature. Eq (25) is drawn for four values of the q parameter. The default value used in the simulations is $q = 2$.

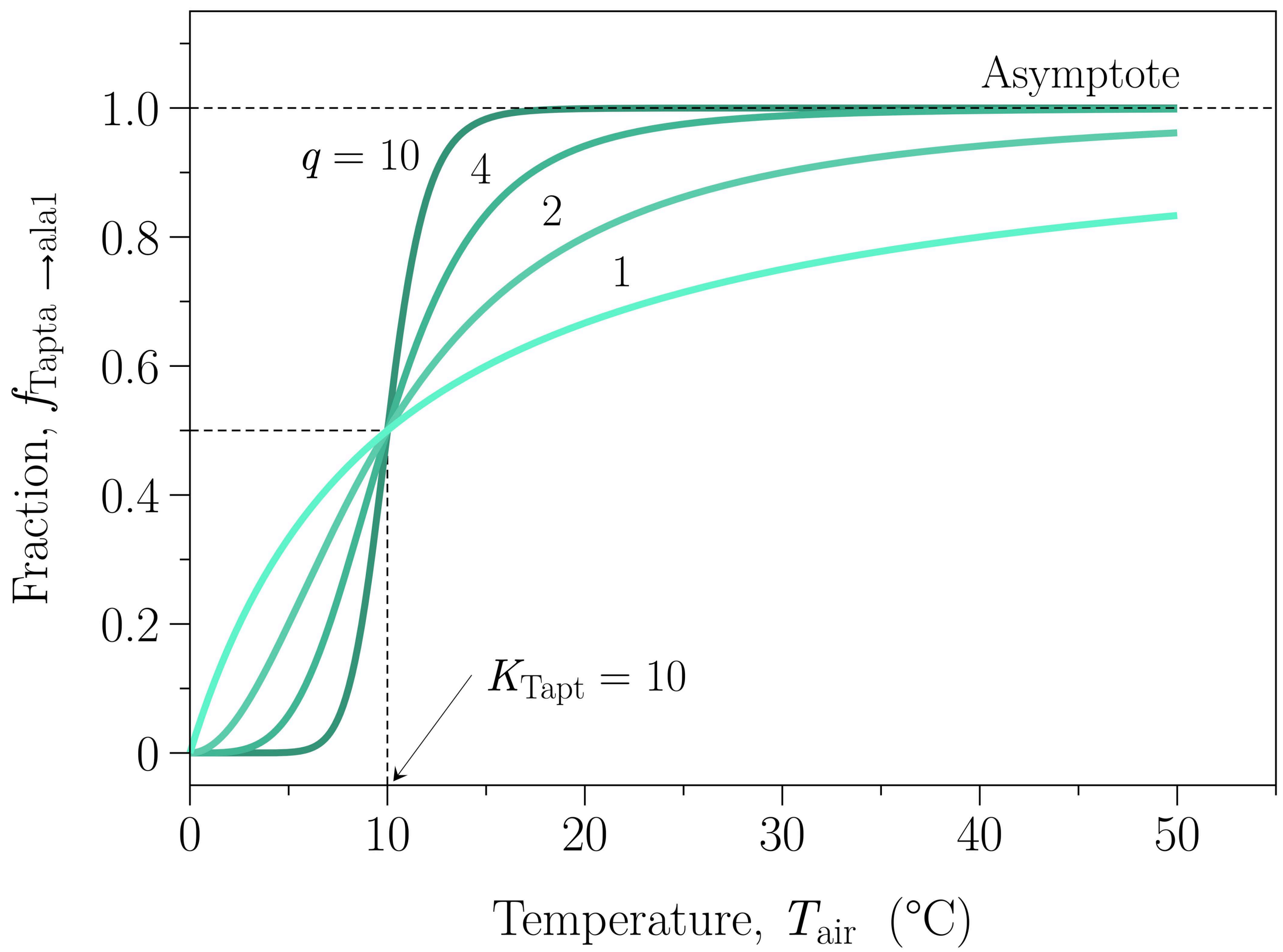

Fig D.7. Elements contributing to the fraction of apterous offspring which are alates. Illustration of how the three different components contribute to the fraction of apterous offspring which are alates, as in Eq (28). This is for an Eskdalemuir environment (Section 4.1) and the first year of growth of typical spruce plantation (Section 4.2) infected with ten alate adults at time zero [Eq (64)]. **A**, air temperature, T_{air} , via Eq (25). **B**, aphid density, ρ_{aph}, via Eq (26). **C**, phloem N, N_{phloem}, via Eq (27). Here, the introduction of aphids at time zero, depresses N_{phloem}, the lower continuous line, resulting in a higher fraction destined for alates (upper dashed line). **D**, the three factors shown in **A**, **B**, **C** are combined in Eq (28).

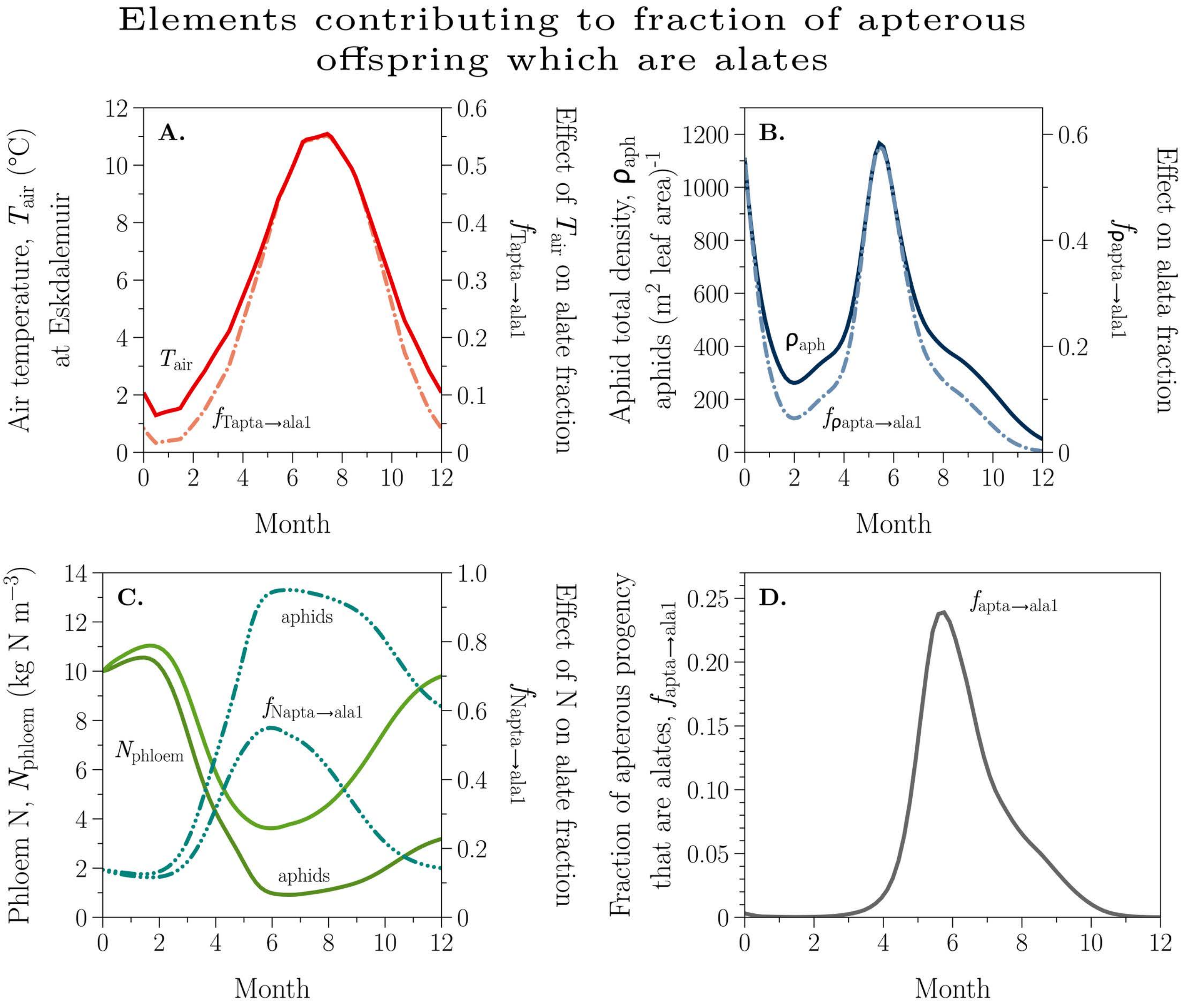

D.3.4 Total fecundity and the fractional fecundity of apterous adults

Now that we have calculated the fractions of offspring from apterous adults which are alate and which are apterous, $f_{\mathrm{apta\to ala1}}$ and $f_{\mathrm{apta\to apt1}}$ in Eq (28), we are able to calculate the total fecundity of apterous adults. It is assumed that N ingested by apterous adult females per stem per day, $I_{\mathrm{Nphloem\to apta}}$ [Eq (24)], is converted without loss into first-instar nymphs, giving a total rate of output to first instar offspring of $O_{\mathrm{apta\to ala1}} + O_{\mathrm{apta\to apt1}}$, denoted by $O_{\mathrm{apta\to axx1}}$ [with Eq (28)], of (aphids stem^{-1} d^{-1})

$$\begin{aligned} O_{\mathrm{apta\to axx1}} &= O_{\mathrm{apta\to ala1}} + O_{\mathrm{apta\to apt1}} \\ &= \frac{I_{\mathrm{Nphloem\to apta}}}{f_{\mathrm{apta\to apt1}} M_{\mathrm{Napta1}} + f_{\mathrm{apta\to ala1}} M_{\mathrm{Nala1}}}, \qquad (29a) \\ f_{\mathrm{fecapta}} &= \frac{O_{\mathrm{apta\to axx1}}}{a_{\mathrm{pta}}}. \qquad (29b) \end{aligned}$$

The aphid N contents are given in Eq (7). Equation (29b) defines the total fractional fecundity of apterous adults, f_{fecapta} (d^{-1}); this is the total output of nymphs from apterous adults (a_{pta}) towards the alate and apterous first-instar pools divided by the number of apterous adults (a_{pta}). The transfer of nymph outputs into inputs into the first-instar pools is assumed to occur without loss giving (aphids stem^{-1} d^{-1})

$$O_{\mathrm{apta\to apt1}} = f_{\mathrm{apta\to apt1}} O_{\mathrm{apta\to axx1}}, \quad O_{\mathrm{apta\to ala1}} = f_{\mathrm{apta\to ala1}} O_{\mathrm{apta\to axx1}}; \qquad (30a)$$

$$I_{\mathrm{apta\to apt1}} = O_{\mathrm{apta\to apt1}}; \quad I_{\mathrm{apta\to ala1}} = O_{\mathrm{apta\to ala1}}. \qquad (30b)$$

D.3.5 Associated N and C fluxes

There are output N fluxes from the a_{pta} pool associated with these aphid fluxes of (kg N stem^{-1} d^{-1}):

$$O_{\mathrm{Napta\to apt1}} = I_{\mathrm{apta\to apt1}} M_{\mathrm{Napt1}}, \quad O_{\mathrm{Napta\to ala1}} = I_{\mathrm{apta\to ala1}} M_{\mathrm{Nala1}}. \qquad (31a)$$

$$\text{Total: } O_{\mathrm{Napta\to axx1}} = O_{\mathrm{Napta\to apt1}} + O_{\mathrm{Napta\to ala1}}. \qquad (31b)$$

The right-side quantities for the first two equations are in Eq (30) and Eq (7).

There are carbon fluxes from apterous adults to respiration, offspring and honeydew [input to the surface litter metabolic pool, Eq (107)]. The carbon intake of the apterous adults is $I_{\mathrm{Cphloem\to apta}}$ [Eq (24), kg C stem^{-1} d^{-1}]. It is assumed that a fraction, $f_{\mathrm{Caph,resp}}$, of this is respired, giving a respiratory flux from apterous adults of (kg C stem^{-1} d^{-1})

$$R_{\mathrm{apta}} = f_{\mathrm{Caph,resp}} I_{\mathrm{Cphloem\to apta}}, \; f_{\mathrm{Caph,resp}} = 0.5. \qquad (32)$$

This value of 0.5 is typical of respiration losses [50].

Some carbon is used for first instar offspring (kg C stem^{-1} d^{-1}):

$$O_{\text{Capta}\to\text{apt1}} = I_{\text{apta}\to\text{apt1}} M_{\text{Capt1}}, \quad O_{\text{Capta}\to\text{ala1}} = I_{\text{apta}\to\text{ala1}} M_{\text{Cala1}}, \tag{33a}$$

$$\text{Total: } O_{\text{Capta}\to\text{axx1}} = O_{\text{Capta}\to\text{apt1}} + O_{\text{Capta}\to\text{ala1}}. \tag{33b}$$

Here we have used Eqs (7) and (30).

The rest is honeydew (kg C stem^{-1} d^{-1}) [with Eqs (24), (32) and (33)]

$$O_{\text{Capta}\to\text{hon}} = I_{\text{Cphloem}\to\text{apta}} - R_{\text{apta}} - O_{\text{Capta}\to\text{apt1}} - O_{\text{Capta}\to\text{ala1}}. \tag{34}$$

This output is input to the surface litter metabolic pool [Eq (107)].

D.4 Development rates of apterous aphids

These depend principally on temperature (T_{air}) and to a lesser extent on nutrition [N_{phloem}, Eq (2)]. Aphid density does not seem to be a factor. We assume that day length is not important. For the temperature modifier of aphid development, $f_{\text{Taph,dev}}$, the standard biological temperature response function is assumed as in Eq (17) but with one different parameter, T_0' (Fig D.4D):

$$f_{\text{Taph,dev}} = f(T_{\text{air}}, T_0' = T'_{0,\text{aph,dev}} = 30\,^\circ\text{C}). \tag{35}$$

The Fig D.4D $T_0{}'$ = 30 curve can be compared qualitatively with Duffy et al. ([20], their figure 3; see Section D.4.1 below).

The nutritional effect is small: a 10% decrease in development rate if N_{phloem} [Eq (2)] is zero so that $f_{\text{Naph,dev,min}} = 0.9$. The nutritional modifier of developmental rate, $f_{\text{Naph,dev}}$, is assumed to be given by a Michaelis-Menten term with:

$$f_{\text{Naph,dev}} = f_{\text{Naph,dev,min}} + (1 - f_{\text{Naph,dev,min}}) \frac{N_{\text{phloem}}}{N_{\text{phloem}} + K_{\text{Naph,dev}}}, \tag{36a}$$

$$f_{\text{Naph,dev,min}} = 0.9, \quad K_{\text{Naph,dev}} = 1 \text{ kg N m}^3. \tag{36b}$$

The K value, giving half-maximal nutritional effect, is equivalent to foliage N substrate concentration of $N_{\text{le}} = 0.01$ kg N substrate (kg structural dry matter)$^{-1}$ [Eq (2)]. If $N_{\text{phloem}} = 0$, $f_{\text{Naph,dev}} = 0.9$; if $N_{\text{phloem}} = K_{\text{Naph,dev}}$, $f_{\text{Naph,dev}} = 0.95$; N_{phloem} is large, $f_{\text{Naph,dev}} = 1$. Note that in the Fig 4 +0 °C run, $f_{\text{Naph,dev}}$ is mostly about 0.97 + 0.02 and N_{phloem} is in the range 2 to 7 [Eq (2)] so the effect of nutrition on developmental rate is small.

The combined effects of temperature [Eq (35), Fig D.4D, T_0' = 30 °C curve] and N nutrition on development [Eq (36)] are

$$f_{\text{TNaph,dev}} = f_{\text{Taph,dev}} f_{\text{Naph,dev}}. \tag{37}$$

All the 20 °C apterous rate constants (d^{-1}) are modified by this dimension-free factor:

$$k_{\text{apt1}\to 2} = k_{\text{apt1}\to 2,20} f_{\text{TNaph,dev}}, \quad k_{\text{apt2}\to 3} = k_{\text{apt2}\to 3,20} f_{\text{TNaph,dev}}, \tag{38a}$$

$$k_{\text{apt3}\to 4} = k_{\text{apt3}\to 4,20} f_{\text{TNaph,dev}}, \quad k_{\text{apt4}\to \text{a}} = k_{\text{apt4}\to \text{a},20} f_{\text{TNaph,dev}}. \tag{38b}$$

$$k_{\text{apt1}\to 2,20} = 0.2222^*, \quad k_{\text{apt2}\to 3,20} = 0.25, \tag{38c}$$

$$k_{\text{apt3}\to 4,20} = 0.2857143, \quad k_{\text{apt4}\to \text{a},20} = 0.3333^* \text{ d}^{-1}, \ * \text{ denotes recurring.} \tag{38d}$$

The lifetimes, τ(d) , associated with these 20 °C rate parameters are

$$\tau_{\text{apt1}\to 2,20} = 1/k_{\text{apt1}\to 2,20} = 4.5, \quad \tau_{\text{apt2}\to 3,20} = 1/k_{\text{apt2}\to 3,30} = 4.0; \tag{39a}$$

$$\tau_{\text{apt3}\to 4,20} = 1/k_{\text{apt3}\to 4,20} = 3.5, \quad \tau_{\text{apt4}\to \text{a},20} = 1/k_{\text{apt4}\to \text{a},30} = 3.0 \text{ d}. \tag{39b}$$

Summing, over all $\tau_{\mathrm{apt1\to a,20}} = 15$ d.

The overall value for the transition from 1st apterous instar to apterous adult agrees reasonably with Dixon ([55], p. 109, table 6.1). Our attempts to relate present assumptions to the data, equations and figures of Duffy et al. [20] were unsuccessful. It is assumed that alates develop 15% more slowly than apterous aphids [Section D.9, Eqs (9) and (80)].

D.4.1 Survival

As mentioned in the first paragraph of Section D.1.2, survival is a concept used by some authors (e.g. [20]). In this subsection we show how it is related to the present formulation.

Considering the first instar apterous aphids, a_{apt1}, the rate of transfer (units d^{-1}) to the 2nd instar compartment is $k_{\mathrm{apt1\to2}}$ [Eq (38), Fig 2]. This competes with aphid mortality, $k_{\mathrm{aph,mort}}$ [Eq (11), Fig D.3A], resulting in a survival probability which is a function of time, survival (t), whose asymptote is survival $(t \to \infty)$. Both quantities are dimensionless.

$$s_{\mathrm{urvival}}(t) = \frac{k_{\mathrm{apt1\to2}}}{k_{\mathrm{apt1\to2}} + k_{\mathrm{aph,mort}}}\left[1 - \mathrm{e}^{-(k_{\mathrm{apt1\to2}}+k_{\mathrm{aph,mort}})t}\right]; \tag{40a}$$

$$s_{\mathrm{urvival}}(t \to \infty) = \frac{k_{\mathrm{apt1\to2}}}{k_{\mathrm{apt1\to2}} + k_{\mathrm{aph,mort}}}. \tag{40b}$$

The latter is drawn in Fig D.8B and the quantities determining this in Fig D.8A.

Figure 3 of Duffy et al. [20] compares reasonably with the present formalism and Fig D.8B. The survival fraction is a consequence of two competing rate constants, mortality and development. It may therefore be more pertinent to consider the underlying rate constants shown in Fig D.8A.

D.5 Juvenile apterous (wingless) aphids

It is convenient to deal with these pools together (Fig 2). The four instars have state variables, $a_{\mathrm{pt1,\ldots,4}}$ (aphids per stem). First the pool inputs are defined, then the outputs and last the differential equations.

D.5.1 Inputs: juvenile apterous (wingless) aphids

These are: $I_{\mathrm{apt}\jmath}$, $\jmath = 1, \ldots, 4$ (aphids stem^{-1} d^{-1}), with (Fig 2)

$$I_{\mathrm{apt1}} = I_{\mathrm{apta\to apt1}} + I_{\mathrm{alaa\to apt1}}, \quad I_{\mathrm{apt2}} = O_{\mathrm{apt1\to2}} \tag{41a}$$

$$I_{\mathrm{apt3}} = O_{\mathrm{apt2\to3}}, \quad I_{\mathrm{apt4}} = O_{\mathrm{apt3\to4}}. \tag{41b}$$

The inputs to the first pool are from the reproduction of both apterous and alate adults [Fig 2; Eqs (30) and (74)]. The inputs to the other three pools are equal to the developmental outputs of the preceding pools [Eq (42)], assuming that transfer takes place without loss.

Fig D.8. Survival. The temperature dependence of the survival function, Eq (40). **A**, the terms making up the function are shown: $k_{\mathrm{apt1}\rightarrow 2}$ is the specific rate of transfer of aphids from the 1st instar apterous compartment to the second [Fig 2, Eq (38)]; $k_{\mathrm{aph,mort}}$ [Eq (11), **A**] is the specific aphid mortality rate. **B**, the asymptotic survival function $s_{urvival}(t \rightarrow \infty)$ [Eq (40)]. The four points shown are from Duffy et al. ([20], their figure 3), whose data are from Dean [56].

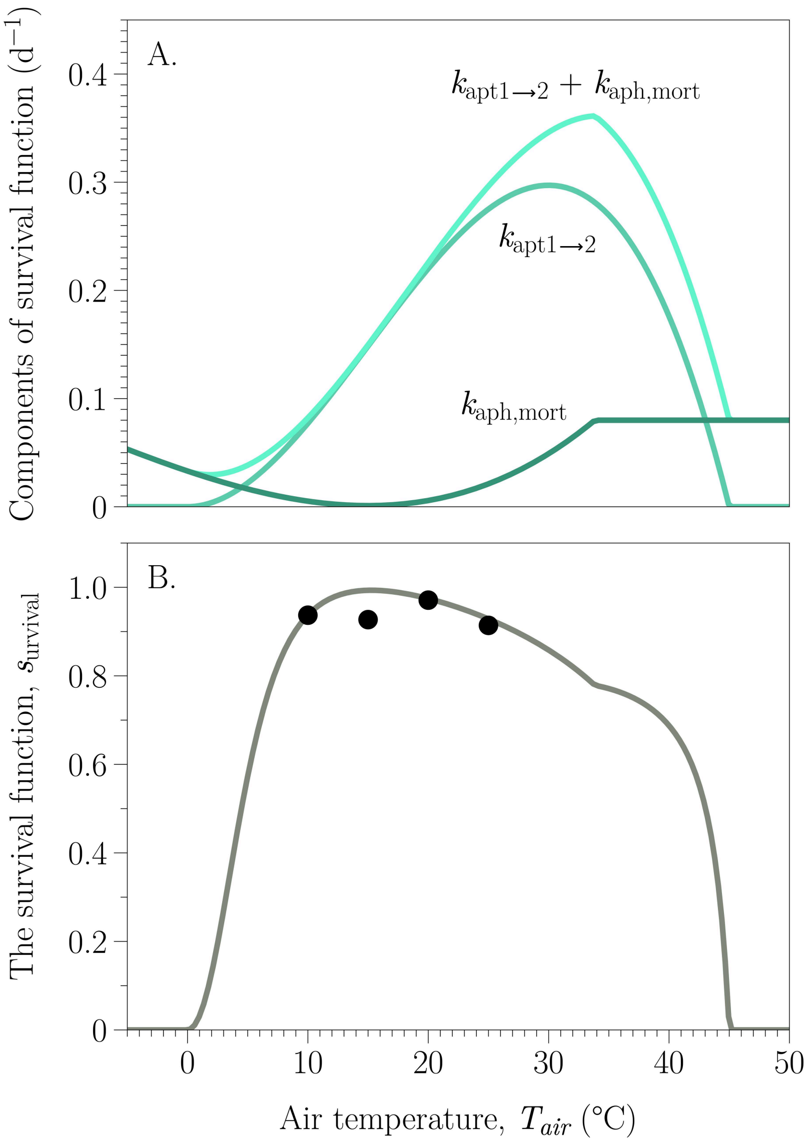

D.5.2 Outputs: juvenile apterous (wingless) aphids

The outputs, $O_{\mathrm{apt}\jmath}$, $\jmath = 1, \ldots 4$ (aphids stem^{-1} d^{-1}) from each pool arise from development, mortality, tree thinning and/or foliage pruning. There is no loss of aphid numbers per stem to thinning when whole stems are removed.

Development. Developmental outputs are (aphids stem^{-1} d^{-1})

$$O_{\mathrm{apt1\to2}} = k_{\mathrm{apt1\to2}} a_{\mathrm{pt1}}, \;\; O_{\mathrm{apt2\to3}} = k_{\mathrm{apt2\to3}} a_{\mathrm{pt2}} \tag{42a}$$
$$O_{\mathrm{apt3\to4}} = k_{\mathrm{apt3\to4}} a_{\mathrm{pt3}}, \;\; O_{\mathrm{apt4\to a}} = k_{\mathrm{apt4\to a}} a_{\mathrm{pt4}}. \tag{42b}$$

The developmental rate constants (d^{-1}) are given in Eq (38). The state variables, $a_{\mathrm{pt}\jmath}$, $\jmath = 1, \ldots, 4$ (aphids stem^{-1}) are defined by Eq (47) (Fig 2). The outputs are transferred without loss, providing the inputs (aphids stem^{-1} d^{-1}) of $I_{\mathrm{apt2-4}}$ in Eq (41) and also the input to apterous adults of

$$I_{\mathrm{apta}} = I_{\mathrm{apt4\to a}} = O_{\mathrm{apt4\to a}}. \tag{43}$$

Mortality. It is assumed that the same mortality rate constant, $k_{\mathrm{aph,mort}}$ [Eq (11)], applies to all four instars, leading to outputs of (aphids stem^{-1} d^{-1})

$$O_{\mathrm{apt1\to mort}} = k_{\mathrm{apt,mort}} a_{\mathrm{pt1}}, \;\; O_{\mathrm{apt2\to mort}} = k_{\mathrm{apt,mort}} a_{\mathrm{pt2}}, \tag{44a}$$
$$O_{\mathrm{apt3\to mort}} = k_{\mathrm{apt,mort}} a_{\mathrm{pt3}}, \;\; O_{\mathrm{apt4\to mort}} = k_{\mathrm{apt,mort}} a_{\mathrm{pt4}}, \tag{44b}$$
$$O_{\mathrm{apti}} = O_{\mathrm{apt1\to mort}} + O_{\mathrm{apt2\to mort}} + O_{\mathrm{apt3\to mort}} + O_{\mathrm{apt4\to mort}}, \tag{44c}$$
$$O_{\mathrm{apt\to mort}} = O_{\mathrm{apti\to mort}} + O_{\mathrm{apta\to mort}}. \tag{44d}$$

In the last two lines we calculate total instar mortality, $O_{\mathrm{apti\to mort}}$ and total apterous aphid mortality, $O_{\mathrm{apt\to mort}}$.

Pruning. Pruning is assumed to remove aphids from the aphid number per stem pools, as in Eq (14). The output fluxes are (aphids stem^{-1} d^{-1})

$$O_{\mathrm{apt1\to prune}} = k_{\mathrm{apt,prune}} a_{\mathrm{pt1}}, \;\; O_{\mathrm{apt2\to prune}} = k_{\mathrm{apt,prune}} a_{\mathrm{pt2}}, \tag{45a}$$
$$O_{\mathrm{apt3\to prune}} = k_{\mathrm{apt,prune}} a_{\mathrm{pt3}}, \;\; O_{\mathrm{apt4\to prune}} = k_{\mathrm{apt},prune} a_{pt4}, \tag{45b}$$
$$O_{\mathrm{apti}} = O_{\mathrm{apt1\to prune}} + O_{\mathrm{apt2\to prune}} + O_{\mathrm{apt3\to prune}} + O_{\mathrm{apt4\to prune}}, \tag{45c}$$
$$O_{\mathrm{apt\to prune}} = O_{\mathrm{apti\to prune}} + O_{\mathrm{apta\to prune}}. \tag{45d}$$

The state variables, $a_{\mathrm{pt}\jmath}$, $\jmath = 1, \ldots, 4$ (aphids stem^{-1}) are defined by Eq (47) (Fig 2). The last two of Eq (45) give the total apterous instar output to pruning and with the first of Eq (14), total outputs of apterous instars and total apterous aphids to pruning. See Section D.1.2 'mortality'. The pruning rate constant $k_{\mathrm{le\to prune}} = 0$ d^{-1} in these aphid studies [Eq (14)].

Combining Eqs (42), (44) and (45), the total outputs from the four pools are (aphids stem^{-1} d^{-1}):

$$O_{\mathrm{apt1}} = O_{\mathrm{apt1\to2}} + O_{\mathrm{apt1\to mort}} + O_{\mathrm{apt1\to prune}}, \tag{46a}$$
$$O_{\mathrm{apt2}} = O_{\mathrm{apt2\to3}} + O_{\mathrm{apt2\to mort}} + O_{\mathrm{apt2\to prune}}, \tag{46b}$$
$$O_{\mathrm{apt3}} = O_{\mathrm{apt3\to4}} + O_{\mathrm{apt3\to mort}} + O_{\mathrm{apt3\to prune}}, \tag{46c}$$
$$O_{\mathrm{apt4}} = O_{\mathrm{apt4\to a}} + O_{\mathrm{apt4\to mort}} + O_{\mathrm{apt4\to prune}}. \tag{46d}$$

D.5.3 Differential equations: juvenile apterous (wingless) aphids

With Eqs (41) and (46), the differential equations (aphids stem^{-1} d^{-1}) and initial values for the four apterous instar state variables are (aphids stem^{-1}):

$$\frac{da_{pt\jmath}}{dt} = I_{apt\jmath} - O_{apt\jmath}, \quad \text{for } \jmath = 1, \ldots 4. \tag{47}$$

At $t = 0$ d: $a_{pt\jmath} = 0$ for $\jmath = 1, \ldots, 4$ aphids stem^{-1}.

D.5.4 Nitrogen and carbon fluxes for the four apterous instar pools

It is assumed that the N taken up from the phloem is the amount required to increase the N component of mass of the aphids which enter the next pool. Therefore, using an obvious notation (kg N stem^{-1} d^{-1}):

$$I_{\text{Nphloem}\to\text{apt1}} = O_{\text{apt1}\to 2}(M_{\text{Napt2}} - M_{\text{apt1}}), \tag{48a}$$
$$I_{\text{Nphloem}\to\text{apt2}} = O_{\text{apt2}\to 3}(M_{\text{Napt3}} - M_{\text{apt2}}), \tag{48b}$$
$$I_{\text{Nphloem}\to\text{apt3}} = O_{\text{apt3}\to 4}(M_{\text{Napt4}} - M_{\text{apt3}}), \tag{48c}$$
$$I_{\text{Nphloem}\to\text{apt4}} = O_{\text{apt4}\to 2}(M_{\text{Napta}} - M_{\text{apt4}}). \tag{48d}$$

Aphid N contents are given in Eq (7); developmental outputs in Eq (42). Total N intake from the phloem by the four apterous instars (apti) is (kg N stem^{-1} d^{-1}):

$$I_{\text{Nphloem}\to\text{apti}} = I_{\text{Nphloem}\to\text{apt1}} + I_{\text{Nphloem}\to\text{apt2}} + I_{\text{Nphloem}\to\text{apt3}} + I_{\text{Nphloem}\to\text{apt4}} \tag{49}$$

The C flux accompanying this N flux is (kg C stem^{-1} d^{-1}):

$$I_{\text{Cphloem}\to\text{apti}} = r_{\text{CNphloem}} I_{\text{Nphloem}\to\text{apti}}. \tag{50}$$

The phloem C:N ratio is given in Eq (3).

The C taken from the phloem is used for respiration, increase in instar mass and honeydew. The earlier treatment is followed [Eqs (32)–(34)].

The amount respired is (kg C stem^{-1} d^{-1})

$$R_{\text{apti}} = f_{\text{Caph,resp}} I_{\text{Cphloem}\to\text{apti}}, \tag{51}$$

where fraction, $f_{\text{Caph,resp}}$, is defined by Eq (32).

The increase in instar mass from aphid development (dev) also requires C from the phloem. The four input (I) components of this and the total phloem C output to supply these four inputs are (kg C stem^{-1} d^{-1})

$$I_{\text{Cphloem}\to\text{apt1}} = O_{\text{apt1}\to 2}(M_{\text{Capt2}} - M_{\text{Capt1}}), \tag{52a}$$
$$I_{\text{Cphloem}\to\text{apt2}} = O_{\text{apt2}\to 3}(M_{\text{Capt3}} - M_{\text{Capt2}}), \tag{52b}$$
$$I_{\text{Cphloem}\to\text{apt3}} = O_{\text{apt3}\to 4}(M_{\text{Capt4}} - M_{\text{Capt3}}), \tag{52c}$$
$$I_{\text{Cphloem}\to\text{apt4}} = O_{\text{apt4}\to\text{a}}(M_{\text{Capta}} - M_{\text{Capt4}}), \tag{52d}$$
$$O_{\text{Cphloem}\to\text{apti}} = I_{\text{Cphloem}\to\text{apt1}} + I_{\text{Cphloem}\to\text{apt2}} + I_{\text{Cphloem}\to\text{apt3}} + I_{\text{Cphloem}\to\text{apt4}}. \tag{52e}$$

Equations (42) and (7) are used in the above.

Excess C is excreted as honeydew (hon), which is input to the surface litter metabolic pool [Eq (107)] ([45], chapter 5; kg C stem^{-1} d^{-1}):

$$O_{\text{Capti}\to\text{hon}} = I_{\text{Cphloem}\to\text{apti}} - R_{\text{apti}} - O_{\text{Cphloem}\to\text{apti}}. \tag{53}$$

Eqs (50), (51) and (52) are used here.

Mortality (mort) gives rise to C and N outputs of (kg C, N stem^{-1} d^{-1}):

$$O_{\text{Capti}\to\text{mort}} = O_{\text{apt1}\to\text{mort}} M_{\text{Capt1}} + O_{\text{apt2}\to\text{mort}} M_{\text{Capt2}} + O_{\text{apt3}\to\text{mort}} M_{\text{Capt3}} + O_{\text{apt4}\to\text{mort}} M_{\text{Capt4}}, \tag{54a}$$

$$O_{\text{Napti}\to\text{mort}} = O_{\text{apt1}\to\text{mort}} M_{\text{Napt1}} + O_{\text{apt2}\to\text{mort}} M_{\text{Napt2}} + O_{\text{apt3}\to\text{mort}} M_{\text{Napt3}} + O_{\text{apt4}\to\text{mort}} M_{\text{Napt4}}. \tag{54b}$$

Equations (44) and (7) have been applied. These fluxes are input to the soil surface litter pools [Eqs (108) and (118)].

Pruning similarly gives rise to C and N outputs of (kg C, N stem^{-1} d^{-1}):

$$O_{\text{Capti}\to\text{prune}} = O_{\text{apt1}\to\text{prune}} M_{\text{Capt1}} + O_{\text{apt2}\to\text{prune}} M_{\text{Capt2}} + O_{\text{apt3}\to\text{prune}} M_{\text{Capt3}} + O_{\text{apt4}\to\text{prune}} M_{\text{Capt4}}, \tag{55a}$$

$$O_{\text{Napti}\to\text{prune}} = O_{\text{apt1}\to\text{prune}} M_{\text{Napt1}} + O_{\text{apt2}\to\text{prune}} M_{\text{Napt2}} + O_{\text{apt3}\to\text{prune}} M_{\text{Napt3}} + O_{\text{apt4}\to\text{prune}} M_{\text{Napt4}}. \tag{55b}$$

Equations (45) and (7) have been used, but see Eq (14).

D.6 Alate (winged) adult aphids, a_{laa}

D.6.1 Inputs: alate (winged) adult aphids

In this case [*cf.* Eq (8) and above], there are potentially two inputs. The first is from development of the 4th alate instar, a_{la4}; this input, $I_{\text{ala4}\to\text{alaa}}$, is calculated in Eq (83). The second is from immigration, currently equated to zero [but see Eq (64) where a_{laa} is set to a non-zero value]:

$$I_{\text{imm}\to\text{alaa}} = 0 \text{ aphids stem}^{-1}\text{ d}^{-1}. \tag{56}$$

The total input to the alate adult pool is (aphids stem^{-1} d^{-1})

$$I_{\text{alaa}} = I_{\text{ala4}\to\text{alaa}} + I_{\text{imm}\to\text{alaa}}. \tag{57}$$

This requires C and N fluxes (kg C, N stem^{-1} d^{-1}) of [*cf.* Eq (8)]:

$$I_{\text{Cala}\to\text{a}} = I_{\text{alaa}} M_{\text{Cala}}, \quad I_{\text{Nala}\to\text{a}} = I_{\text{alaa}} M_{\text{Nala}}. \tag{58}$$

We have used Eq (7).

D.6.2 Outputs: alate (winged) adult aphids

There is assumed to be no aphid loss accompanying the foliage litter flux. Thinning does not affect aphid numbers per stem. There are outputs to mortality, pruning and emigration.

Mortality. The aphid flux to mortality, $O_{\text{alaa}\to\text{mort}}$ (aphid number stem^{-1} d^{-1}), is assumed to depend on temperature and nutrition. The specific rate constant, $k_{\text{aph,mort}}$ (d^{-1}) assumed to be the same for all aphid morphs, is calculated in Eq (11). The output from the adult alate pool to mortality and the accompanying C and N fluxes are [*cf.* Eqs (12) and (13)]

$$O_{\text{alaa}\to\text{mort}} = k_{\text{aph,mort}} a_{\text{alaa}}, \tag{59a}$$

$$O_{\text{Cala}\to\text{mort}} = O_{\text{alaa}\to\text{mort}} M_{\text{Cala}}, \quad O_{\text{Nala}\to\text{mort}} = O_{\text{alaa}\to\text{mort}} M_{\text{Nala}}. \tag{59b}$$

Aphid C and N contents are in Eq (7). Units are aphids stem^{-1} d^{-1} and kg C, N stem^{-1} d^{-1}. These fluxes are input to the soil surface litter pools [Eqs (108) and (118)].

Pruning. The pruning flux, $O_{\text{alaa}\to\text{prune}}$ (aphids stem^{-1} d^{-1}) and the associated fluxes of C and N, are, after Eq (14) and with Eqs (7) and (14):

$$O_{\text{alaa}\to\text{prune}} = k_{\text{le,prune}} a_{\text{alaa}}, \tag{60a}$$
$$O_{\text{Cala}\to\text{prune}} = O_{\text{alaa}\to\text{prune}} M_{\text{Cala}}, \;\; O_{\text{Nala}\to\text{prune}} = O_{\text{alaa}\to\text{prune}} M_{\text{Nala}}. \tag{60b}$$

Units of the last two equations are kg aphid C, N stem^{-1} d^{-1}.

Emigration. This flux, $O_{\text{alaa}\to\text{emi}}$ (aphid number stem^{-1} d^{-1}) [Eq (61)], is equal to a standard temperature (20 °C) rate constant, $k_{\text{alaa}\to\text{emi20}}$, multiplied by a temperature-dependent function $f(T_{\text{air}})$ for which the default parameter values are applied [Eq (17), Fig D.4]. Also, at low aphid densities [ρ_{aph}, Eq (5)] so that the incoming alates do not immediately depart, emigration is switched off by multiplying by $f_{\rho\text{alaa}\to\text{ala1}}$ [Eq (71), *cf.* Eq (26), Fig D.7B]. The combined rate parameter is (units: d^{-1})

$$k_{\text{alaa}\to\text{emi}} = k_{\text{alaa}\to\text{emi20}} f(T_{\text{air}}) f_{\rho\text{alaa}\to\text{ala1}}; \;\; k_{\text{alaa}\to\text{emi20}} = 1\,\text{d}^{-1}. \tag{61}$$

When the aphids are crowded, aphid density ρ_{aph} is large, $f_{\rho\text{alaa}\to\text{ala1}} \to 1$ [Eq (71)] and emigration of adult alates occurs almost immediately. With the initial $t = 0$ values of $a_{\text{ph}} = a_{\text{laa}} = 10$ aphids per stem [Eqs Eq (64) and Eq (4)] and $A_{\text{leaf,aph}} = 0.009$ [Eq (5)], $\rho_{\text{aph}} = 1111.1$ [Eq (5)] and $f_{\rho\text{alaa}\to\text{ala1}} = 0.552$. Also at $t = 0$, $T_{\text{air}} = 1.8$ °C (Fig 3A), so that $f(T_{\text{air}}) = 0.019$. Therefore [with Eq (7)]

$$O_{\text{alaa}\to\text{emi}} = k_{\text{alaa}\to\text{emi}} a_{\text{alaa}}, \tag{62a}$$
$$\text{at } t = 0,\; O_{\text{alaa}\to\text{emi}} = 0.103\,\text{aphids stem}^{-1}\,\text{d}^{-1} \tag{62b}$$
$$O_{\text{Calaa}\to\text{emi}} = O_{\text{alaa}\to\text{emi}} M_{\text{Cala}}, \;\; O_{\text{Nala}\to\text{emi}} M_{\text{Nala}}. \tag{62c}$$
$$O_{\text{alaa}\to\text{emi,ya}} = \int_{t-365}^{t} O_{\text{alaa}\to\text{emi}}\,\text{d}t. \tag{62d}$$

Alate adults (a_{laa}, Fig 2) are the only morph that is able to emigrate. Units are aphids stem^{-1} d^{-1} and kg C, N stem^{-1} d^{-1}. Equation (62d) gives the yearly accumulation (ya) of emigrants (aphids stem^{-1} d^{-1}). For the simulation shown in Fig 4, +0 °C which is chaotic, this also behaves chaotically. The total output of adult alate aphids (aphids stem^{-1} d^{-1}) is obtained by adding the contributions [Eqs (55), (60) and (62); *cf.* Eq (15)]:

$$O_{\text{alaa}} = O_{\text{alaa}\to\text{mort}} + O_{\text{alaa}\to\text{prune}} + O_{\text{alaa}\to\text{emi}}. \tag{63}$$

O_{alaa} is used in Eq (99) to calculate fractional outputs.

D.6.3 Differential equation: alate (winged) adult aphids

The differential equation (adult alates stem^{-1} d^{-1}) and initial value for the state variable, a_{laa}, are [with Eqs (57) and (63)]

$$\frac{\text{d}a_{\text{laa}}}{\text{d}t} = I_{\text{alaa}} - O_{\text{alaa}}, \;\; a_{\text{laa}}(t = 0) = 10\,\text{alate adults stem}^{-1}. \tag{64}$$

It is assumed that ten alate (winged) adult aphids fly in and colonize the stem at a specified time (usually $t = 0$ d).

D.7 Alate aphid fecundity

The effect of temperature on alate fecundity is assumed to be the same as in Eq (18) for apterous aphid fecundity. The effect of nutrition on fecundity, manifest here through the N concentration in the phloem, N_{phloem} [Eq (2)], can be made specific — i.e. different for alate and apterous aphids, although here we assume apterous and alate adult aphids are equally fecund. The relevant equations are summarized below [*cf.* Eqs (19) to (24) for details]; results are given in Fig D.5.

The maximum volume intake rate of an alate adult, $v_{\text{alaa,max}}$ (m^3 aphid^{-1} d^{-1}), places a limit on the maximum N intake from the phloem, giving a maximum phloem-volume-limited N intake [combining and modifying Eqs (19) and (20), but keeping the same parameter values]:

$$v_{\text{alaa,max}} = f_{\text{Taph,fec}} v_{\text{alaa,max20}}, \tag{65a}$$

$$v_{\text{alaa,max20}} = 0.2 \times 10^{-6}\ \text{m}^3\ \text{aphid}^{-1}\ \text{d}^{-1}, \text{ where} \tag{65b}$$

$$I_{\text{Nphloem,valaa,max}} = v_{\text{alaa,max}} N_{\text{phloem}}. \tag{65c}$$

See Eq (18) for $f_{\text{Taph,fec}}$. Units of the last equation are kg N aphid^{-1} d^{-1}.

The maximum amount of N which can be processed (p) is [*cf.* Eq(21)]

$$I_{\text{Npalaa,max}} = f_{\text{Taph,fec}} I_{\text{Npalaa,max20}}, \tag{66a}$$

$$I_{\text{Npalaa,max20}} = 2 \times 10^{-6}\ \text{kg N aphid}^{-1}\ \text{d}^{-1}. \tag{66b}$$

The N ingested per adult alate is the least of the volume-intake-limited [Eq (65)] or the processing-limited [Eq (66)] values (kg N aphid^{-1} d^{-1}):

$$I_{\text{Nphloem}\to\text{alaa,aphid}} = \min\{I_{\text{Nphloem,valaa,aphid}}, I_{\text{Npalaa,max}}\}. \tag{67}$$

Dividing by N_{phloem} [Eq (2)], the actual volume of phloem sap ingested per alate adult aphid is (m^3 phloem sap aphid^{-1}) [*cf.* Eq (23)]

$$v_{\text{phloem}\to\text{alaa}} = \frac{I_{\text{Nphloem}\to\text{alaa,aphid}}}{N_{\text{phloem}}}. \tag{68}$$

The C intake per aphid (kg C aphid^{-1} d^{-1}) and then the N and C intakes per stem (kg N, C stem^{-1} d^{-1}) are [*cf.* Eq (24)]:

$$I_{\text{Cphloem}\to\text{alaa,aphid}} = v_{\text{phloem}\to\text{alaa}} C_{\text{phloem}}, \tag{69a}$$

$$I_{\text{Nphloem}\to\text{alaa}} = a_{\text{laa}} I_{\text{Nphloem}\to\text{alaa,aphid}}, \tag{69b}$$

$$I_{\text{Cphloem}\to\text{alaa}} = a_{\text{laa}} I_{\text{Cphloem}\to\text{alaa,aphid}}. \tag{69c}$$

Here Eqs (68), (2), (64) and (67) are employed.

Fig D.9. Fractional output fluxes. Fractional output fluxes of alate adults to mortality, $f_{\text{Oalaa}\rightarrow\text{mort}}$ (solid line) and to emigration, $f_{\text{Oalaa}\rightarrow\text{emi}}$ [Eq (99)] (dash-dot line) over three years post-infection [Eq (64)] at time $t = 0$ for a plantation in a northern Britain environment (Section 4.1). The state variable for alate adults a_{alaa} (Fig 2) is shown by the dashed-dot-dot-dot line [Eq (64)] with reference to the right side ordinate; initial value is 10 and is therefore not shown.

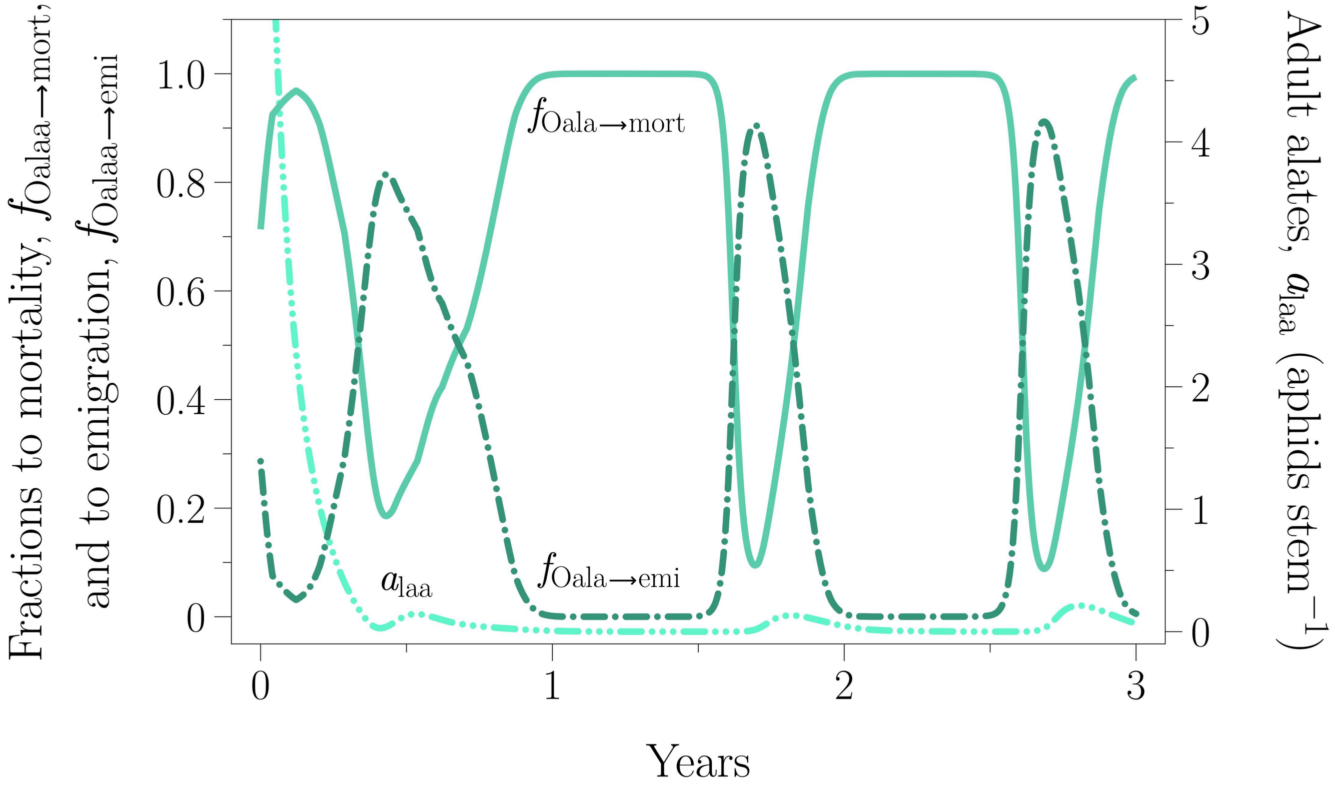

D.8 Alate:apterous ratio in offspring from alate adults

The treatment above for the alate:apterous ratio in the offspring of apterous adults is followed exactly (Section D.3), but with the possibility of different parameters for the alates. Eqs (25)–(28) become

$$f_{\text{Talaa}\rightarrow\text{ala1}} = \frac{(T_{\text{air}})^{q_{\text{Tala}}}}{(T_{\text{air}})^{q_{\text{Tala}}} + (K_{\text{Tala}})^{q_{\text{Tala}}}}, \quad q_{\text{Tala}} = 2, \quad K_{\text{Tala}} = 10\,^{\circ}\text{C}. \tag{70}$$

$$f_{\rho\text{alaa}\rightarrow\text{ala1}} = \frac{(\rho_{\text{aph}})^{q_{\rho\text{ala}}}}{(\rho_{\text{aph}})^{q_{\rho\text{ala}}} + (K_{\rho\text{aph,ala}})^{q_{\rho\text{ala}}}}, \tag{71a}$$

$$q_{\rho\text{ala}} = 2, \quad K_{\rho\text{aph,ala}} = 1000\,\text{aphids}\,(\text{m}^2\ \text{leaf area})^{-1}. \tag{71b}$$

$$f_{\text{Nala}\rightarrow\text{ala1}} = \frac{(K_{\text{Nala}})^{q_{\text{Nala}}}}{(K_{\text{Nala}})^{q_{\text{Nala}}} + (N_{\text{phloem}})^{q_{\text{Nala}}}}, \tag{72a}$$

$$q_{\text{Nala}} = 2, \quad K_{\text{Nala}} = 4\,\text{kg N m}^{-3}. \tag{72b}$$

$$f_{\text{alaa}\rightarrow\text{ala1}} = f_{\text{Tala}\rightarrow\text{ala1}} f_{\rho\text{alaa}\rightarrow\text{ala1}} f_{\text{Nala}\rightarrow\text{ala1}}. \tag{73a}$$

$$f_{\text{alaa}\rightarrow\text{ala1}} = 1 - f_{\text{alaa}\rightarrow\text{ala1}}. \tag{73b}$$

These quantities are drawn in Fig D.5 for the alate fractions of the progeny of apterous adults, where the parameterization is assumed the same as for alate adults.

D.8.1 Total fecundity and fractional fecundity of alate adults

Now that we have calculated the fractions of offspring from alate adults which are alate and which are apterous, $f_{\text{alaa}\rightarrow\text{ala1}}$ and $f_{\text{alaa}\rightarrow\text{apt1}}$ in Eq (73), we are able to calculate the total fecundity of alate adults, Oalaa→axx1 (1st instar aphids stem^{-1} d^{-1}) [*cf.* Section D.3.4, Eq (29) for apterous adults].

The N ingested by alate adults [$I_{\text{Nphloem}\rightarrow\text{alaa}}$, (kg N stem^{-1} d^{-1}), Eq (69)] is converted into alate and apterous first-instar nymphs, giving a total output of first-instar nymphs [*cf.* Eq (29)] and outputs and inputs to these pools with [*cf.* Eq (30)]

$$O_{\text{alaa}\rightarrow\text{axx1}} = O_{\text{alaa}\rightarrow\text{ala1}} + O_{\text{alaa}\rightarrow\text{apt1}} \tag{74a}$$

$$= \frac{I_{\text{Nphloem}\rightarrow\text{alaa}}}{f_{\text{alaa}\rightarrow\text{apt1}} M_{\text{Napt1}} + f_{\text{alaa}\rightarrow\text{ala1}} M_{\text{Nala1}}}, \tag{74b}$$

$$O_{\text{alaa}\rightarrow\text{apt1}} = f_{\text{alaa}\rightarrow\text{apt1}} O_{\text{alaa}\rightarrow\text{axx1}}, \quad O_{\text{alaa}\rightarrow\text{ala1}} = f_{\text{alaa}\rightarrow\text{ala1}} O_{\text{alaa}\rightarrow\text{axx1}}, \tag{74c}$$

$$I_{\text{alaa}\rightarrow\text{apt1}} = O_{\text{alaa}\rightarrow\text{apt1}}, \quad I_{\text{alaa}\rightarrow\text{ala1}} = O_{\text{alaa}\rightarrow\text{ala1}}. \tag{74d}$$

$$f_{\text{fecalaa}} = \frac{O_{\text{alaa}\rightarrow\text{axx1}}}{a_{\text{laa}}}. \tag{74e}$$

Here the total 1st instar progeny output from alate adults is $O_{\text{alaa}\rightarrow\text{axx1}}$ (aphids stem^{-1} d^{-1}) and the total fractional fecundity of alate adults is f_{fecalaa} (d^{-1}).

D.8.2 Associated N and C fluxes

The N fluxes associated with these fluxes of aphids are (kg N stem^{-1} d^{-1})

$$O_{\text{Nalaa}\rightarrow\text{apt}} = I_{\text{alaa}\rightarrow\text{apt1}} M_{\text{Napt1}}, \quad O_{\text{Nala}\rightarrow\text{ala1}} M_{\text{Nala1}}, \tag{75a}$$

$$\text{with a total of } O_{\text{Nalaa}\rightarrow\text{axx1}} = O_{\text{Nalaa}\rightarrow\text{apt1}} + O_{\text{Nalaa}\rightarrow\text{ala1}}. \tag{75b}$$

The carbon intake of the alate adults is $I_{\mathrm{Cphloem}\to\mathrm{alaa}}$ [Eq (69), (kg C stem^{-1} d^{-1})]. There are carbon fluxes (kg C stem^{-1} d^{-1}) to respiration, offspring and the residual C flux goes to honeydew [*cf.* Eqs (32) to (34)]:

$$R_{\mathrm{alaa}} = f_{\mathrm{Caph,resp}} I_{\mathrm{Cphloem}\to\mathrm{alaa}}. \tag{76}$$

$$O_{\mathrm{Calaa}\to\mathrm{ala1}} = I_{\mathrm{alaa}\to\mathrm{ala1}} M_{\mathrm{Cala1}}, O_{\mathrm{Calaa}\to\mathrm{apt1}} = I_{\mathrm{alaa}\to\mathrm{apt1}} M_{\mathrm{Capt1}}. \tag{77}$$

$$O_{\mathrm{Calaa}\to\mathrm{hon}} = I_{\mathrm{Cphloem}\to\mathrm{alaa}} - R_{\mathrm{alaa}} - O_{\mathrm{Calaa}\to\mathrm{ala1}} - O_{\mathrm{Calaa}\to\mathrm{apt1}}. \tag{78}$$

Here on the right side of Eqs (76) to (78) we have used Eqs (32), (69), (74) and (7). The honeydew output is input to the surface litter metabolic pool [see Eq (107) and above].

D.9 Development rates of alate aphids

These are treated as in Eqs (35) to (37) (Section D.4) for apterous aphids, although development rates are slightly different for alate instars. We replace Eq (38) by

$$k_{\mathrm{ala1}\to 2} = k_{\mathrm{ala1}\to 2,20} f_{\mathrm{TNaph,dev}}, \quad k_{\mathrm{ala2}\to 3} = k_{\mathrm{ala2}\to 3,20} f_{\mathrm{TNaph,dev}}, \tag{79a}$$

$$k_{\mathrm{ala3}\to 4} = k_{\mathrm{ala3}\to 4,20} f_{\mathrm{TNaph,dev}}, \quad k_{\mathrm{ala4}\to \mathrm{a}} = k_{\mathrm{ala4}\to \mathrm{a},20} f_{\mathrm{TNaph,dev}}. \tag{79b}$$

$$k_{\mathrm{ala1}\to 2,20} = 0.1888^{*}, \quad k_{\mathrm{ala2}\to 3,20} = 0.2125, \tag{79c}$$

$$k_{\mathrm{ala3}\to 4,20} = 0.24285699, \quad k_{\mathrm{ala4}\to \mathrm{a},20} = 0.283333^{*}\ \mathrm{d}^{-1}, \ ^{*} \text{ denotes recurring.} \tag{79d}$$

Here $f_{\mathrm{TNaph,dev}}$ is given by Eq (37). The lifetimes associated with these 20 °C rate constants are

$$\tau_{\mathrm{ala1}\to 2,20} = 1/k_{\mathrm{ala1}\to 2,20} = 5.294, \quad \tau_{\mathrm{ala2}\to 3,20} = 1/k_{\mathrm{ala2}\to 3,20} = 4.706, \tag{80a}$$

$$\tau_{\mathrm{ala3}\to 4,20} = 1/k_{\mathrm{ala3}\to 4,20} = 4.118, \quad \tau_{\mathrm{ala4}\to \mathrm{a},20} = 1/k_{\mathrm{ala4}\to \mathrm{a},20} = 3.529\ \mathrm{d}. \tag{80b}$$

$$\text{Summing, overall } \tau_{\mathrm{ala1}\to \mathrm{a},20} = 17.6\ \mathrm{d}. \tag{80c}$$

The overall value agrees reasonably with Dixon ([55], p. 109, their table 6.1). Alates develop 15% more slowly than apterous aphids [*loc. cit.*, Dixon refers to Fisher [58] for data on the green spruce aphid]. See also Section D.4.1 on survival.

D.10 Juvenile alate (winged) aphids

There are four instars, with state variables $a_{\mathrm{la1,\ldots,4}}$ (aphids per stem) (Fig 2). The treatment is the same as above for the apterous instars (Section D.5) where the pools are treated together, defining first the inputs, then the outputs and last the differential equations. The equations are summarized below.

D.10.1 Inputs: juvenile alate (winged) aphids

Inputs, $I_{\mathrm{ala}\jmath}$, $\jmath = 1, \ldots, 4$ (aphids stem^{-1} d^{-1}), to these pools are [Fig 2, *cf.* Eq (41)]

$$I_{\mathrm{ala1}} = I_{\mathrm{apta}\to\mathrm{ala1}} + I_{\mathrm{alaa}\to\mathrm{ala1}}, \quad I_{\mathrm{ala2}} = O_{\mathrm{ala1}\to 2} \tag{81a}$$

$$I_{\mathrm{ala3}} = O_{\mathrm{ala2}\to 3}, \quad I_{\mathrm{ala4}} = O_{\mathrm{ala3}\to 4}. \tag{81b}$$

The inputs to the first pool are from the reproduction of both apterous and alate adults [Fig 2, Eqs (30) and (74)]. The inputs to the other three pools are equal to the developmental outputs of the preceding pool [Eq (82)]. It is assumed that transfer takes place without loss.

D.10.2 Outputs: juvenile alate (winged) aphids

The outputs, $O_{\mathrm{ala}\jmath}$, $\jmath = 1, \ldots, 4$, arise from development, mortality and tree/foliage pruning. There is no loss of aphid numbers per stem to thinning when whole stems are removed.

Development. The developmental output fluxes from the four alate instars are [*cf.* Eq (42)] (aphids stem^{-1} d^{-1}):

$$O_{\mathrm{ala1\to2}} = k_{\mathrm{ala1\to2}} a_{\mathrm{la1}}, \quad O_{\mathrm{ala2\to3}} = k_{\mathrm{ala2\to3}} a_{\mathrm{la2}}, \tag{82a}$$

$$O_{\mathrm{ala3\to4}} = k_{\mathrm{ala3\to4}} a_{\mathrm{la3}}, \quad O_{\mathrm{ala4\to a}} = k_{\mathrm{ala4\to a}} a_{\mathrm{la4}}. \tag{82b}$$

Rate constants are in Eq (79). State variables are defined in Eq (87). These outputs are assumed to occur without loss, providing the inputs (aphids stem^{-1} d^{-1}) of $I_{\mathrm{ala2-4}}$ in Eq (81) and also a developmental input to alate adults [see Eq (57)] of

$$I_{\mathrm{ala4\to alaa}} = O_{\mathrm{ala4\to a}}. \tag{83}$$

Mortality. These are [with Eqs (11) and (87); *cf.* Eq (44)] (aphids stem^{-1} d^{-1}):

$$O_{\mathrm{ala1\to mort}} = k_{\mathrm{aph\to mort}} a_{\mathrm{la1}}, \quad O_{\mathrm{ala2\to mort}} = k_{\mathrm{aph\to mort}} a_{\mathrm{la2}}, \tag{84a}$$

$$O_{\mathrm{ala3\to mort}} = k_{\mathrm{aph\to mort}} a_{\mathrm{la3}}, \quad O_{\mathrm{ala4\to mort}} = k_{\mathrm{aph\to mort}} a_{\mathrm{la4}}. \tag{84b}$$

$$O_{\mathrm{alai\to mort}} = O_{\mathrm{ala1\to mort}} + O_{\mathrm{ala2\to mort}} + O_{\mathrm{ala3\to mort}} + O_{\mathrm{ala4\to mort}}, \tag{84c}$$

$$O_{\mathrm{ala\to mort}} = O_{\mathrm{alai\to mort}} + O_{\mathrm{alaa\to mort}}. \tag{84d}$$

It is assumed here that the same mortality rate constant applies to alate and apterous instars. The last two of Eq (84) give the total alate instar and the total alate output to mortality.

Pruning. These are [with Eqs (14) and (87); *cf.* Eq (45)] (aphids stem^{-1} d^{-1}):

$$O_{\mathrm{ala1\to prune}} = k_{\mathrm{le\to prune}} a_{\mathrm{la1}}, \quad O_{\mathrm{ala2\to prune}} = k_{\mathrm{le\to prune}} a_{\mathrm{la2}}, \tag{85a}$$

$$O_{\mathrm{ala3\to prune}} = k_{\mathrm{le\to prune}} a_{\mathrm{la3}}, \quad O_{\mathrm{ala4\to prune}} = k_{\mathrm{le\to prune}} a_{\mathrm{la4}}. \tag{85b}$$

$$O_{\mathrm{alai\to prune}} = O_{\mathrm{ala1\to prune}} + O_{\mathrm{ala2\to prune}} + O_{\mathrm{ala3\to prune}} + O_{\mathrm{ala4\to prune}}, \tag{85c}$$

$$O_{\mathrm{ala\to prune}} = O_{\mathrm{alai\to prune}} + O_{\mathrm{alaa\to prune}}, \tag{85d}$$

$$O_{\mathrm{aph\to prune}} = O_{\mathrm{ala\to prune}} + O_{\mathrm{apt\to prune}}. \tag{85e}$$

The last three of Eq (85) give first, the total alate instar output to pruning, $O_{\mathrm{alai\to prune}}$; next, with the first of Eq (60), total output of alate aphids to pruning, $O_{\mathrm{ala\to prune}}$; and last, the total aphid output to pruning, $O_{\mathrm{aph\to prune}}$, using the last of Eq (45). See Section D.5.2 'pruning,' for pruning of apterous aphids. The pruning rate constant $k_{\mathrm{le\to prune}} = 0$ d^{-1} in the aphid studies presented here [Eq (14)].

D.10.3 Differential equations: juvenile alate (winged) aphids

Total outputs for each of the four instar pools are [with Eqs (82), (84) and (85)] [*cf.* Eq (64)]

$$O_{\mathrm{ala1}} = O_{\mathrm{ala1\to2}} + O_{\mathrm{ala1\to mort}} + O_{\mathrm{ala1\to prune}}, \tag{86a}$$

$$O_{\mathrm{ala2}} = O_{\mathrm{ala2\to3}} + O_{\mathrm{ala2\to mort}} + O_{\mathrm{ala2\to prune}}, \tag{86b}$$

$$O_{\mathrm{ala3}} = O_{\mathrm{ala3\to4}} + O_{\mathrm{ala3\to mort}} + O_{\mathrm{ala3\to prune}}, \tag{86c}$$

$$O_{\mathrm{ala4}} = O_{\mathrm{ala4\to a}} + O_{\mathrm{ala4\to mort}} + O_{\mathrm{ala4\to prune}}. \tag{86d}$$

Combining Eqs (81) and (86), the differential equations and initial values for the four state variables are (aphids stem^{-1} d^{-1}) [*cf.* Eq (47)]:

$$\frac{\mathrm{d}a_{\mathrm{la}j}}{\mathrm{d}t} = I_{\mathrm{ala}j} - O_{\mathrm{ala}j}, \quad \text{for } j = 1, \ldots, 4. \tag{87}$$

At $t = 0$ d, $a_{\mathrm{la1}} = a_{\mathrm{la2}} = a_{\mathrm{la3}} = a_{\mathrm{la4}} = 0$ aphids stem^{-1}.

D.10.4 Nitrogen and carbon fluxes for the four alate instar pools

Assume the N required from phloem is the amount required to increase the N component of aphid mass entering the next pool. Therefore with Eq (82) [*cf.* Eq (48)]:

$$I_{\mathrm{Nphloem\to ala1}} = O_{\mathrm{ala1\to 2}}\left(M_{\mathrm{Nala2}} - M_{\mathrm{Nala1}}\right), \tag{88a}$$
$$I_{\mathrm{Nphloem\to ala2}} = O_{\mathrm{ala2\to 3}}\left(M_{\mathrm{Nala3}} - M_{\mathrm{Nala2}}\right), \tag{88b}$$
$$I_{\mathrm{Nphloem\to ala3}} = O_{\mathrm{ala3\to 4}}\left(M_{\mathrm{Nala4}} - M_{\mathrm{Nala3}}\right), \tag{88c}$$
$$I_{\mathrm{Nphloem\to ala4}} = O_{\mathrm{ala4\to a}}\left(M_{\mathrm{Nala4}} - M_{\mathrm{Nala3}}\right). \tag{88d}$$

Units are: kg N stem^{-1} d^{-1}. Aphid N contents are in Eq (7). Total N taken from the phloem by the four instars (alai) is

$$I_{\mathrm{Nphloem\to alai}} = \sum_{j=1}^{4} I_{\mathrm{Nphloem\to ala}j}. \tag{89}$$

The C flux from the phloem accompanying this N flux is [Eq (3), *cf.* Eq (50)]:

$$I_{\mathrm{Cphloem\to alai}} = r_{\mathrm{CNphloem}} I_{\mathrm{Nphloem\to alai}}. \tag{90}$$

Respiration flux is (kg C stem^{-1} d^{-1})

$$R_{\mathrm{alai}} = f_{\mathrm{Caph,resp}} I_{\mathrm{Cphloem\to alai}}. \tag{91}$$

$f_{\mathrm{Caph,resp}}$ is given in Eq (32).

The increase in instar mass from aphid instar development also requires C from the phloem. The four input (I) components of this and the total phloem C output to supply these four inputs are [with Eqs (82) and (7); *cf.* Eq (52)] (kg C stem^{-1} d^{-1}):

$$I_{\mathrm{Cphloem\to ala1}} = O_{\mathrm{ala1\to 2}}\left(M_{\mathrm{Cala2}} - M_{\mathrm{Cala1}}\right), \tag{92a}$$
$$I_{\mathrm{Cphloem\to ala2}} = O_{\mathrm{ala2\to 3}}\left(M_{\mathrm{Cala3}} - M_{\mathrm{Cala2}}\right), \tag{92b}$$
$$I_{\mathrm{Cphloem\to ala3}} = O_{\mathrm{ala3\to 4}}\left(M_{\mathrm{Cala4}} - M_{\mathrm{Cala3}}\right), \tag{92c}$$
$$I_{\mathrm{Cphloem\to ala4}} = O_{\mathrm{ala4\to a}}\left(M_{\mathrm{Calaa}} - M_{\mathrm{Cala4}}\right). \tag{92d}$$
$$I_{\mathrm{Cphloem\to alai}} = I_{\mathrm{Cphloem\to ala1}} + I_{\mathrm{Cphloem\to ala2}} + I_{\mathrm{Cphloem\to ala3}} + I_{\mathrm{Cphloem\to ala4}}. \tag{92e}$$

The excess C is excreted as honeydew (input to the surface litter metabolic pool, Eq (107), see Thornley [45], chapter 5, *cf.* Eq (53)]:

$$O_{\mathrm{Calai\to hon}} = I_{\mathrm{Cphloem\to alai}} - R_{\mathrm{alai}} - O_{\mathrm{Cphloem\to alai}}. \tag{93}$$

This makes use of Eqs (90)–(92).

Mortality gives rise to C and N outputs [Eqs(7) and (84), *cf.* Eq (54)] (kg C, N stem^{-1} d^{-1}):

$$O_{\mathrm{Calai\to mort}} = O_{\mathrm{ala1\to mort}} M_{\mathrm{Cala1}} + O_{\mathrm{ala2\to mort}} M_{\mathrm{Cala2}} + O_{\mathrm{ala3\to mort}} M_{\mathrm{Cala3}} + O_{\mathrm{ala4\to mort}} M_{\mathrm{Cala4}}, \tag{94a}$$
$$O_{\mathrm{Nalai\to mort}} = O_{\mathrm{ala1\to mort}} M_{\mathrm{Nala1}} + O_{\mathrm{ala2\to mort}} M_{\mathrm{Nala2}} + O_{\mathrm{ala3\to mort}} M_{\mathrm{Nala3}} + O_{\mathrm{ala4\to mort}} M_{\mathrm{Nala4}}. \tag{94b}$$

The C, N mortality fluxes above are captured in Eqs (108) and (118) and thence in Eqs (112) and (126) on the way to the surface litter cellulose and lignin pools [Eq (127)].

Pruning similarly gives rise to C and N outputs [with Eqs (85) and (7), *cf.* Eq (55)] (kg C, N stem^{-1} d^{-1}):

$$O_{\text{Calai}\to\text{prune}} = O_{\text{ala1}\to\text{prune}} M_{\text{Cala1}} + O_{\text{ala2}\to\text{prune}} M_{\text{Cala2}} + O_{\text{ala3}\to\text{prune}} M_{\text{Cala3}} + O_{\text{ala4}\to\text{prune}} M_{\text{Cala4}}, \tag{95a}$$

$$O_{\text{Nalai}\to\text{prune}} = O_{\text{ala1}\to\text{prune}} M_{\text{Nala1}} + O_{\text{ala2}\to\text{prune}} M_{\text{Nala2}} + O_{\text{ala3}\to\text{prune}} M_{\text{Nala3}} + O_{\text{ala4}\to\text{prune}} M_{\text{Nala4}}. \tag{95b}$$

These fluxes are captured in Eqs (110) and (120) and thence in Eqs (112) and (112).

D.11 Aphid mortality, emigration and fecundity

Here, we calculate total mortality, emigration and fecundity for the aphids.

D.11.1 Mortality

Aphid fluxes to mortality are summed to give a total of $O_{\text{aph}\to\text{mort}}$ (aphid number stem^{-1} d^{-1}) of

$$O_{\text{aph}\to\text{mort}} = O_{\text{apta}\to\text{mort}} + O_{\text{apti}\to\text{mort}} + O_{\text{alaa}\to\text{mort}} + O_{\text{alai}\to\text{mort}}. \tag{96}$$

Here we have used Eqs (12), (44), (59) and (84). Because these fluxes vary greatly both diurnally and seasonally, it can be helpful to take yearly accumulations (ya) with

$$O_{\text{aph}\to\text{mortya}} = \int_{t-365}^{t} O_{\text{aph}\to\text{mort}}\,\mathrm{d}t, \tag{97a}$$

$$a_{\text{phya}} = \int_{t-365}^{t} a_{\text{ph}} \frac{\mathrm{d}t}{365}. \tag{97b}$$

The additional subscript ya denotes yearly accumulation for rate variables such as $O_{\text{aph}\to\text{mort}}$ or a yearly average for non-rate variables such as a_{ph}. $O_{\text{aph}\to\text{mortya}}$ now has units of aphid number stem^{-1} y^{-1}. a_{phya} is a yearly average of a_{ph}, with units of aphid number stem^{-1}.

D.11.2 Emigration

The emigration flux is $O_{\text{alaa}\to\text{emi}}$ (aphids stem^{-1} d^{-1}) [Eq (62)]. Total output from the alate adult pool is O_{alaa} [Eq (63)]. The mortality flux from this pool is $O_{\text{alaa}\to\text{mort}}$ [Eq (59)]. In Fig D.9 the fractional output fluxes to mortality and to emigration are shown. In the absence of pruning [Eq (63)] the two fractional variables defined in Eq (99) add up to unity. Emigration peaks in late summer, when the adult alate population is maximum (Fig D.9) and air temperature is also favourable (Fig 3A).

Because, as far as the local population is concerned, emigration is equivalent to mortality, we also calculate the flux to 'mortality + emigration', which includes the emigration flux from alate adults [$O_{\text{alaa}\to\text{emi}}$, Eq (62)]. Using an obvious notation, therefore

$$O_{\text{alaa}\to\text{mortemi}} = O_{\text{alaa}\to\text{mort}} + O_{\text{alaa}\to\text{emi}}. \tag{98a}$$

$$O_{\text{alaa}\to\text{mortemiya}} = \int_{t-365}^{t} O_{\text{alaa}\to\text{mortemi}}\,\mathrm{d}t. \tag{98b}$$

The terms Eq (98a) have units of aphids stem^{-1} d^{-1}; in Eq (98b) we give the yearly accumulations (aphids stem^{-1} y^{-1}). In fact, the emigration flux is mostly small compared to the total aphid mortality flux (Fig D.9). In Eq (98) and in the simulations shown in Fig D.9, mortality and emigration are the only fluxes from the alate adult pool [Eq (63)] as the pruning fluxes are zero [Eq (14)]. The (dimension-free) fractional outputs are

$$f_{\mathrm{Oalaa\to emi}} = \frac{O_{\mathrm{alaa\to emi}}}{O_{\mathrm{alaa}}}, \quad f_{\mathrm{Oalaa\to mort}} = \frac{O_{\mathrm{alaa\to mort}}}{O_{\mathrm{alaa}}}. \tag{99}$$

The fractional fate of alate adults is drawn in Fig D.9. Aphids in the alate adult pool, a_{laa}, may either die or emigrate [Fig 2 and Eq (63). The fractions are calculated in Eq (99). After an initial transient the fraction emigrating, $f_{\mathrm{Oalaa\to emi}}$ drops to a few percent and then peaks as the temperature rises [Fig 3A, Eq (17), Fig D.6, default temperature response]. Mortality rate is close to zero at these moderate temperatures [Eqs (11) and (9), Fig D.3]. Alate adult numbers per stem, a_{laa}, increase each year as leaf area available to the aphids, $A_{\mathrm{leaf,aph}}$, increases [Eq (5)].

D.11.3 Fecundity

Total offspring production rate from apterous and alate adults (1st instar aphids stem^{-1} d^{-1}) is

$$O_{\mathrm{aph\to fec}} = O_{\mathrm{apta\to ala1}} + O_{\mathrm{apta\to apt1}} + O_{\mathrm{alaa\to ala1}} + O_{\mathrm{alaa\to apt1}}, \tag{100a}$$

$$O_{\mathrm{aph\to fecya}} = \int_{t-365}^{t} O_{\mathrm{aph\to fec}}\,\mathrm{d}t. \tag{100b}$$

Here we utilized Eqs (30) and (74). Eq (100a) gives the instantaneous rate and Eq (100b) the yearly accumulation (1st instar aphids stem^{-1} y^{-1}).

D.11.4 Total aphid number per stem, a_{ph}

The ten state variable differential-equation components of a_{ph} (Fig 2) of Eq 4, namely Eqs (64), (87), (16) and (47) are added to give $\mathrm{d}a_{\mathrm{ph}}/\mathrm{d}t$:

$$\frac{\mathrm{d}a_{\mathrm{ph}}}{\mathrm{d}t} = \frac{\mathrm{d}a_{\mathrm{alaa}}}{\mathrm{d}t} + \sum_{j=1}^{4}\frac{\mathrm{d}a_{\mathrm{ala}j}}{\mathrm{d}t} + \frac{\mathrm{d}a_{\mathrm{apta}}}{\mathrm{d}t} + \sum_{j=1}^{4}\frac{\mathrm{d}a_{\mathrm{apt}j}}{\mathrm{d}t}. \tag{101}$$

Putting immigration, $I_{\mathrm{imm\to alaa}} = 0$ [Eq (56)] and noting that alate adults (a_{laa}) are the only morph able to emigrate, therefore

$$\frac{\mathrm{d}a_{\mathrm{ph,chk}}}{\mathrm{d}t} = O_{\mathrm{aph\to fec}} - O_{\mathrm{aph\to mort}} - O_{\mathrm{alaa\to emi}} - O_{\mathrm{aph\to prune}}. \tag{102}$$

Units are aphids stem^{-1} d^{-1}. Here we make use of Eqs (100), (96), (62) and (85). The equality of the values of Eqs (101) and (102) gives a useful check on the accuracy of the programming.

D.12 Aphid sub-model – C and N balances

In this section the inputs and outputs of the whole aphid subsystem are summarized. This has two purposes. First, it is a useful check that the model has been consistently formulated mathematically and is programmed correctly. Second, in long-term ecosystem studies, quite small fluxes are important, as they can lead to degradation, or otherwise, of the ecosystem. These are now made explicit.

D.12.1 Carbon inputs: phloem, immigration

There are two C inputs to the aphid sub-model, from the phloem and from possible immigration of alates. The C input from the phloem is [Eqs (90), (50), (69) and (24)]

$$I_{\text{Cphloem}\to\text{aph}} = I_{\text{Cphloem}\to\text{alai}} + I_{\text{Cphloem}\to\text{apti}} + I_{\text{Cphloem}\to\text{alaa}} + I_{\text{Cphloem}\to\text{apta}}, \quad (103a)$$

$$I_{\text{Cphloem}\to\text{aph,m2}} = n_{\text{stems}} I_{\text{Cphloem}\to\text{aph}}. \quad (103b)$$

The first equation is in units of kg C stem^{-1} d^{-1}, the second, kg C (m^2 ground)$^{-1}$ d^{-1}. n_{stems} is the stem density (number of stems m^{-2}), a state variable of the efm [Eq (1)].

Immigration gives C fluxes of [Eqs (7) and (56)]

$$I_{\text{Cimm}\to\text{alaa}} = I_{\text{imm}\to\text{alaa}} M_{\text{Calaa}}, \quad I_{\text{Cimm}\to\text{alaa,m2}} = n_{\text{stems}} I_{\text{Cimm}\to\text{alaa}}. \quad (104)$$

The first equation is in units of kg C stem^{-1} d^{-1}, the second, kg C (m^2 ground)$^{-1}$ d^{-1}.

Total C inputs to the aphid sub-model are [Eqs (103) and (104)]

$$I_{\text{Caph}} = I_{\text{Cphloem}\to\text{aph}} + I_{\text{Cimm}\to\text{alaa}}, \quad I_{\text{Caph,m2}} = n_{\text{stems}} I_{\text{Caph}}, \quad (105)$$

with units of kg C stem^{-1} d^{-1} and kg C (m^2 ground)$^{-1}$ d^{-1}.

D.12.2 Carbon outputs: respiration, honeydew, mortality, emigration, pruning, thinning

There are outputs via respiration, honeydew, mortality, emigration, pruning and thinning.

Aphid respiration which produces CO_2 gives C fluxes of [Eqs (76), (91), (32), (51)]

$$O_{\text{Caph}\to\text{CO2}} = R_{\text{alaa}} + R_{\text{alai}} + R_{\text{apta}} + R_{\text{apti}}, \quad (106a)$$

$$O_{\text{Caph}\to\text{CO2,m2}} = n_{\text{stems}} O_{\text{Caph}\to\text{CO2}}. \quad (106b)$$

Units are kg C stem^{-1} d^{-1} and kg C (m^2 ground)$^{-1}$ d^{-1}, respectively.

Honeydew production [Eqs (78), (93), (34), (53)] gives an output, $O_{\text{Caph}\to\text{hon,m2}}$, which is input without loss to the surface litter metabolic pool [Eq (12)] ([45], figure 5.1, $C_{\text{surf-li,met}}$):

$$O_{\text{Caph}\to\text{hon}} = O_{\text{Calaa}\to\text{hon}} + O_{\text{Calai}\to\text{hon}} + O_{\text{Capta}\to\text{hon}} + O_{\text{Capti}\to\text{hon}}, \quad (107a)$$

$$O_{\text{Caph}\to\text{hon,m2}} = n_{\text{stems}} O_{\text{Caph}\to\text{hon}}. \quad (107b)$$

Units are kg C stem^{-1} d^{-1} (Eq (107a)) and kg C (m^2 ground)$^{-1}$ d^{-1} (Eq (107b)).

Mortality of aphids gives rise to C fluxes of [Eqs (59), (94), (12) and (54)]

$$O_{\text{Caph}\to\text{mort}} = O_{\text{Calaa}\to\text{mort}} + O_{\text{Calai}\to\text{mort}} + O_{\text{Capta}\to\text{mort}} + O_{\text{Capti}\to\text{mort}}, \quad (108a)$$

$$O_{\text{Caph}\to\text{mort,m2}} = n_{\text{stems}} O_{\text{Caph}\to\text{mort}}. \quad (108b)$$

Units are as in Eq (107). This flux is divided equally between the surface litter cellulose and lignin pools [Eq (126) and Eq (127); figure 5.1 in [45]].

Emigration of adult alates gives rise to a output C flux (kg C stem^{-1} d^{-1}) of $O_{\text{Calaa}\to\text{emi}}$ [Eq (62)] and a C flux per unit area ground of

$$O_{\text{Calaa}\to\text{emi,m2}} = n_{\text{stems}} O_{\text{Calaa}\to\text{emi}}. \quad (109)$$

Pruning the trees gives an output C flux of [Eqs (60), (95), (14), (55)]

$$O_{\text{Caph}\to\text{prune}} = O_{\text{Calaa}\to\text{prune}} + O_{\text{Calai}\to\text{prune}} + O_{\text{Capta}\to\text{prune}} + O_{\text{Capti}\to\text{prune}}, \quad (110a)$$

$$O_{\text{Caph}\to\text{prune,m2}} = n_{\text{stems}} O_{\text{Caph}\to\text{prune}}. \quad (110b)$$

Again, units are kg C stem^{-1} d^{-1} and kg C (m^2 ground)$^{-1}$ d^{-1}. As with the mortality fluxes of Eq (108), it is assumed that pruning produces a flux of dead aphids into the surface litter cellulose and lignin pools [Eqs (126) and (127); figure 5.1 in [45]].

Thinning, which is applied in forest plantation management, removes whole stems (Section 4.2). It does not affect aphid numbers per stem. However, aphids on removed stems are assumed to join the surface litter, together with the foliage on the removed stems (some of the branches and stems may be taken away as timber products). The rate of stem loss per unit ground area is specified by the management regime in the forest model (Section 4.2). It is $O_{\text{nstems}\to\text{th}}$ [Eq (1), stems m^{-2} d^{-1}]. This quantity is zero most of the time, but it is non-zero on the days when the plantation is thinned by an amount specified. Multiplying by C_{aph} [Eq (6), kg aphid C per stem], this leads to an aphid C output flux per unit ground area of (kg C m^{-2} d^{-1})

$$O_{\text{Caph}\to\text{th,m2}} = O_{\text{nstems}\to\text{th}} C_{\text{aph}}. \tag{111}$$

This output is input to the soil surface litter inputs in Eq (126).

Total aphid C outputs are [with Eqs (106)–(108), (110), (62) and (111)]

$$O_{\text{Caph}} = O_{\text{Caph}\to\text{CO2}} + O_{\text{Caph}\to\text{hon}} + O_{\text{Caph}\to\text{mort}} + O_{\text{Caph}\to\text{prune}} + O_{\text{Caph}\to\text{emi}}. \tag{112a}$$
$$O_{\text{Caph,m2}} = n_{\text{stems}} O_{\text{Caph}} + O_{\text{Caph}\to\text{th,m2}}. \tag{112b}$$

The first equation here has units of kg C stem^{-1} d^{-1}; the second units of kg C (m^2 ground)$^{-1}$ d^{-1}.

D.12.3 Carbon balance

A consistency check is to calculate the rate of change of aphid C two ways. First by summing the differential Eqs (64), (87)), (16) and (47), with Eq (7) for the first equation and Eqs (1) and (6) for the second equation:

$$\frac{dC_{\text{aph}}}{dt} = M_{\text{Calaa}} \frac{da_{\text{laa}}}{dt} + \sum_{j=1}^{4} M_{\text{Cal}aj} \frac{da_{\text{la}j}}{dt} + M_{\text{Capta}} \frac{da_{\text{pta}}}{dt} + \sum_{j=1}^{4} M_{\text{Capt}j} \frac{da_{\text{pt}j}}{dt}. \tag{113a}$$
$$\frac{dC_{\text{aph,m2}}}{dt} = n_{\text{stems}} \frac{dC_{\text{aph}}}{dt} + C_{\text{aph}} \frac{dN_{\text{stems}}}{dt}. \tag{113b}$$

These were compared (successfully) with the same (check = chk) quantities calculated with [Eqs (105) and (112)]

$$\frac{dC_{\text{aph,chk}}}{dt} = I_{\text{Caph}} - O_{\text{Caph}}, \tag{114a}$$
$$\frac{dC_{\text{aph,m2,chk}}}{dt} = I_{\text{Caph,m2}} - O_{\text{Caph,m2}}. \tag{114b}$$

D.12.4 Nitrogen inputs: phloem, immigration

The procedure from Eqs (103)–(114) for C is repeated for N. There are the same two N inputs [*cf.* Eqs (103)–(105)], from the phloem and via immigration.

From the phloem, therefore, with Eqs (89), (49), (69), (24) and (1) for n_{stems}:

$$I_{\text{Nphloem}\to\text{aph}} = I_{\text{Nphloem}\to\text{alai}} + I_{\text{Nphloem}\to\text{apti}} + I_{\text{Nphloem}\to\text{alaa}} + I_{\text{Nphloem}\to\text{apta}}. \tag{115a}$$
$$I_{\text{Nphloem}\to\text{aph,m2}} = n_{\text{stems}} I_{\text{Nphloem}\to\text{aph}}. \tag{115b}$$

Units are kg N stem^{-1} d^{-1} and kg N (m^2 ground)$^{-1}$ d^{-1}.

Immigration gives N fluxes of [Eqs (7) and (56)]:

$$I_{\text{Nimm}\to\text{alaa}} = I_{\text{imm}\to\text{alaa}} M_{\text{Nalaa}}; \quad I_{\text{Nimm}\to\text{alaa,m2}} = n_{\text{stems}} I_{\text{Naph}}. \tag{116}$$

Units as in Eq (115) .

Total N inputs to the aphid sub-model are [with Eqs (115), (116) and (1)]

$$I_{\text{Naph}} = I_{\text{Nphloem}\to\text{aph}} + I_{\text{Nimm}\to\text{alaa}}, \quad I_{\text{Naph,m2}} = n_{\text{stems}} I_{\text{Naph}}. \tag{117}$$

Units are kg N stem^{-1} d^{-1} and kg N (m^2 ground)$^{-1}$ d^{-1}.

D.12.5 Nitrogen outputs: mortality, emigration, pruning, thinning

There are outputs to mortality, emigration, pruning and thinning.

First, mortality of aphids gives rise to N fluxes of [Eqs (59), (94), (13), (54) and (1)]

$$O_{\text{Naph}\to\text{mort}} = O_{\text{Nala}\to\text{mort}} + O_{\text{Nalai}\to\text{mort}} + O_{\text{Napta}\to\text{mort}} + O_{\text{Napti}\to\text{mort}}, \tag{118a}$$

$$O_{\text{Naph}\to\text{mort,m2}} = n_{\text{stems}} O_{\text{Naph}\to\text{mort}}. \tag{118b}$$

Units are kg N stem^{-1} d^{-1} and kg N (m^2 ground)$^{-1}$ d^{-1}. This dead aphid N flux is divided between the surface litter metabolic N, cellulose and lignin pools (Eqs (126)–(128); figure 5.1 in [45]).

Next emigration of adult alates gives rise to an N flux per stem of $O_{\text{Nalaa}\to\text{emi}}$ [Eq (62)] and an N flux per unit area ground of [for n_{stems}, see Eq (1)]:

$$O_{\text{Nalaa}\to\text{emi,m2}} = n_{\text{stems}} O_{\text{Nalaa}\to\text{emi}}. \tag{119}$$

Pruning the trees gives an output N flux of [Eqs (60), (95), (14) and (55)]

$$O_{\text{Naph}\to\text{prune}} = O_{\text{Nala}\to\text{prune}} + O_{\text{Nalai}\to\text{prune}} + O_{\text{Napta}\to\text{prune}} + O_{\text{Napti}\to\text{prune}}, \tag{120a}$$

$$O_{\text{Naph}\to\text{prune,m2}} = n_{\text{stems}} O_{\text{Naph}\to\text{prune}}. \tag{120b}$$

Units are kg N stem^{-1} d^{-1} and kg N (m^2 ground)$^{-1}$ d^{-1}.

Thinning gives rise to an aphid N output flux per unit ground area of [Eq (6), see discussion before Eq (111)] (kg N m^{-2} d^{-1})

$$O_{\text{Naph}\to\text{th,m2}} = O_{\text{nstems}\to\text{th}} N_{\text{aph}}. \tag{121}$$

Total aphid N outputs are [with Eqs (118), (120), (62), (1) and (121)]

$$O_{\text{Naph}} = O_{\text{Naph}\to\text{mort}} + O_{\text{Naph}\to\text{prune}} + O_{\text{Naph}\to\text{emi}}, \tag{122a}$$

$$O_{\text{Naph,m2}} = n_{\text{stems}} O_{\text{Naph}} + O_{\text{Naph}\to\text{th,m2}}. \tag{122b}$$

Units are kg N stem^{-1} d^{-1} and kg N (m^2 ground)$^{-1}$ d^{-1}. These outputs are used in Eq (124) for consistency checking and in Eq (126) for calculating inputs to the surface litter pools.

D.12.6 N balance

The same consistency check is applied to N as for C [Eqs (113) and (114)].

$$\frac{dN_{\text{aph}}}{dt} = M_{\text{Nalaa}} \frac{da_{\text{laa}}}{dt} + \sum_{j=1}^{4} M_{\text{Nala}j} \frac{da_{\text{la}j}}{dt} + M_{\text{Napta}} \frac{da_{\text{pta}}}{dt} + \sum_{j=1}^{4} M_{\text{Napt}j} \frac{da_{\text{pt}j}}{dt}. \tag{123a}$$

$$\frac{dN_{\text{aph,m2}}}{dt} = n_{\text{stems}} \frac{dN_{\text{aph}}}{dt} + N_{\text{aph}} \frac{dN_{\text{stems}}}{dt}. \tag{123b}$$

Units of these two equations are kg N stem^{-1} d^{-1} and kg N (m^2 ground)$^{-1}$ d^{-1}. For the first of these sum the differential Eqs (64), (87), (16) and (47) and apply Eq (7); for the second use Eqs (1) and (6). These were compared (successfully) with those calculated with Eqs (117) and (122).

$$\frac{dN_{\text{aph,chk}}}{dt} = I_{\text{Naph}} - O_{\text{Naph}}, \tag{124a}$$

$$\frac{dN_{\text{aph,m2,chk}}}{dt} = I_{\text{Naph,m2}} - O_{\text{Naph,m2}}. \tag{124b}$$

D.13 Soil sub-model inputs from: mortality, pruning and thinning

The C input to the surface litter metabolic C pool ([45], figure 5.1) from honeydew is [Eq (107)] (kg C m^{-2} d^{-1}):

$$I_{\text{Caph}\rightarrow\text{surf-li,met}} = O_{\text{Caph}\rightarrow\text{hon,m2}}. \tag{125}$$

The C inputs to the cellulose and lignin pools are calculated by first totalling the C and N dead aphid fluxes from mortality [Eqs (108) and (118)], pruning [Eqs (110) and (120)] and thinning [Eqs (111) and (121)] (kg C, N m^{-2} d^{-1}):

$$I_{\text{Caph}\rightarrow\text{surf-li}} = O_{\text{Caph}\rightarrow\text{mort,m2}} + O_{\text{Caph}\rightarrow\text{prune,m2}} + O_{\text{Caph}\rightarrow\text{th,m2}}, \tag{126a}$$

$$I_{\text{Naph}\rightarrow\text{surf-li}} = O_{\text{Naph}\rightarrow\text{mort,m2}} + O_{\text{Naph}\rightarrow\text{prune,m2}} + O_{\text{Naph}\rightarrow\text{th,m2}}. \tag{126b}$$

The C flux is partitioned equally between the cellulose and lignin pools:

$$I_{\text{Caph}\rightarrow\text{surf-li,cel}} = f_{\text{Caph,cel}} I_{\text{Caph}\rightarrow\text{surf-li}}, \tag{127a}$$

$$I_{\text{Caph}\rightarrow\text{surf-li,lig}} = \left(1 - f_{\text{Caph,cel}}\right) I_{\text{Caph}\rightarrow\text{surf-li}}, \tag{127b}$$

$$f_{\text{Caph,cel}} = 0.5 \tag{127c}$$

$f_{\text{Caph,cel}}$ is the fraction of C in dead aphids assigned to the surface litter cellulose pool, the remainder entering the lignin pool.

The N flux is handled rather differently, because of the assumption that the cellulose and lignin litter pools have a constant C:N ratio ([45], p. 75; p. 85, equation 5.1c). The N flux required to satisfy this fixed C:N ratio is subtracted from the total dead aphid N flux and the remainder is put into the surface litter metabolic pool. Therefore, with Eqs (127) and (126):

$$I_{\text{Naph}\rightarrow\text{surf-li,cel}} = \frac{I_{\text{Caph}\rightarrow\text{surf-li,cel}}}{R_{\text{CN,cel}}}, \quad I_{\text{Naph}\rightarrow\text{surf-li,lig}} = \frac{I_{\text{Caph}\rightarrow\text{surf-li,lig}}}{r_{\text{CN,cel}}}, \tag{128a}$$

$$I_{\text{Naph}\rightarrow\text{surf-li,met}} = I_{\text{Naph}\rightarrow\text{surf-li}} - I_{\text{Naph}\rightarrow\text{surf-li,cel}} - I_{\text{Naph}\rightarrow\text{surf-li,lig}}. \tag{128b}$$

$$r_{\text{CN,cel}} = 150, \quad r_{\text{CN,cel}} = 100\,\text{kg C (kg N)}^{-1}. \tag{128c}$$

The cellulose and lignin C:N ratios are 150 and 100 kg C (kg N)$^{-1}$.

D.14 Aphid C and N at the end of a rotation

At the end of a rotation, the plantation is clear-felled. It is assumed that the aphid C and N are similar to foliage structural C and N and they are added to the surface litter structural C and N pools on this basis, before the next rotation is begun. See figure 5.1 and equations 5.1b,c of Thornley ([45], p. 76, p. 85).

When initializing for the next rotation, the initial C and N contents of the aphids (C_{aph}, N_{aph} at $t = 0$, expressed per unit ground area) are subtracted from the surface litter metabolic C and N pools, so that small amounts of C and N are not added at the beginning of every rotation when infestation via alate immigration occurs. However this is not important in the present studies as the initial values for the soil and litter sub-model are obtained by multiple rotations in the absence of aphids.

Appendix E Summary of symbols used in the model

Tab E.1. Symbols and definitions. Notation and abbreviations: equation numbers of significant occurrence are given. DM denotes dry matter, C denotes carbon, and N denotes nitrogen.

**Aphid sub-model state variables (Fig 2)**		
**Symbol**	**Definition**	**Values and Units**
$a_{\text{la}\jmath}$, $\jmath = 1,\dots 4$	Alate (winged) instars [Eqs (4), (87)]	0 aphids stem^{-1}
a_{laa}	Alate adults [Eqs (4), (64)]	10 aphids stem^{-1}
$a_{\text{pt}\jmath}$, $\jmath = 1,\dots 4$	Apterous (wingless) instars [Eqs (4), (47)]	0 aphids stem^{-1}
a_{pta}	Apterous adults [Eqs (4), (16)]	0 aphids stem^{-1}
**Parameters**		
**Symbol**	**Definition**	**Values and Units**
$c_{\text{aph,mort,skew}}$	Skewness parameter for temperature dependence of mortality [Eq (9)]	0.2 (dimensionless)
$f_{\text{Caph,cel}}$	Fraction of C in dead aphids assigned to surface litter cellulose [Eq (127)]	0.5
$f_{\text{Caph,resp}}$	Fraction of aphid C from phloem input respired [Eq (32)]	0.5
$f_{\text{Naph,dev,min}}$	Fractional development rate due to low N phloem [Eq (36)]	0.9
$I_{\text{Npalaa,max20}}$, $I_{\text{Npapta,max20}}$	Maximum processing (p) N intake rate at 20 °C per alate, apterous adult [Eqs (21), (66)]	2×10^{-6} kg N aphid^{-1} d^{-1}
$K_{\text{Naph,dev}}$, $K_{\text{Naph,mort}}$	Michaelis-Menten constants for nutrition-dependent development [Eq (36)], mortality [Eq (10)]	1 kg N m^{-3}
K_{Nala}, K_{Napt}, K_{Tala}, K_{Tapt}, $K_{\rho\text{aph,ala}}$, $K_{\rho\text{aph,apt}}$	Michaelis-Menten constants for effects of nutrition, temperature and aphid density on the alate/apterous fractions in offspring [Eqs (72), (27), (70), (25), (71), (26)]	4 kg N m^{-3}, 10 °C, 1000 aphids (m^2 leaf area)$^{-1}$
$k_{\text{ala1}\to\text{2,20}}$, $k_{\text{ala2}\to\text{3,20}}$, $k_{\text{ala3}\to\text{4,20}}$, $k_{\text{ala4}\to\text{a,20}}$; $k_{\text{apt1}\to\text{2,20}}$, $k_{\text{apt2}\to\text{3,20}}$, $k_{\text{apt3}\to\text{4,20}}$, $k_{\text{apt4}\to\text{a,20}}$	Development rate constants of alate and apterous instars at 20 °C [Eqs (79), (38)]	0.18, 0.2125, 0.242857; 0.283; 0.22, 0.25, 0.285714; 0.33* d^{-1}, * denotes recurring

continued on next page

Symbol	Definition	Values and Units
$k_{\text{alaa}\to\text{emi20}}$	Rate constant at 20 °C for emigration of adult alate [Eq (61)]	1 d^{-1}
$k_{\text{aph,Nmort,max}}$	Maximum rate constant for nutrition-dependent aphid mortality [Eq (10)]	0.1 d^{-1}
$k_{\text{aph,Tmort,0}}$, $k_{\text{aph,Tmort,opt}}$	Rate constants for temperature-dependent mortality [Eq (9)]	0.04, 0 d^{-1}
$k_{\text{le}\to\text{prune}}$	Foliage pruning constant [Eq (14)]	0 d^{-1}
$M_{\text{Cala}\jmath}$, $\jmath = 1\ldots4$; M_{Calaa}; $M_{\text{Capt}\jmath}$, $\jmath = 1\ldots4$; M_{Capta}	Carbon mass in alate, apterous instar and adult aphids [Eq (7)]	1, 2, 3, 4; 5; 1, 2, 3, 4; 5, $\times10^{-6}$ kg C aphid^{-1}
$M_{\text{Nala}\jmath}$, $\jmath = 1\ldots4$; M_{Nalaa}; $M_{\text{Napt}\jmath}$, $\jmath = 1\ldots4$; M_{Napta}	Nitrogen mass in alate, apterous instar and adult aphids [Eq (7)]	1, 2, 3, 4; 5; 1, 2, 3, 4, 5, $\times10^{-6}$ kg C aphid^{-1}
m_{CS}, m_{NS}	Multipliers converting mass concentrations into volume concentrations [Eq (2)]	5000, 1000 kg structural DM m^{-3}
q_1, q_2	Exponents in general temperature function [Eq (17)]	2, 1
$q_{\text{aph,Nmort}}$	Sigmoidicity constant for nutrition-dependent (N) aphid mortality [Eq (10)]	2
q_{Tala}, $q_{\rho\text{ala}}$, q_{Nala}; q_{Tapt}, $q_{\rho\text{apt}}$, q_{Napt}	Sigmoidicity constants for effects of temperature, aphid density and nutrition on the alate fraction in alate, apterous offspring [Eqs (70)–(72), (25)–(27)]	2, 2, 2, 2, 2, 2
$r_{\text{CN,cell}}$, $r_{\text{CN,lig}}$	Cellulose, lignin, C:N ratios [Eq (128)]	150, 100 kg C (kg $\text{N})^{-1}$
$T_{\text{aph,mort,0}}$, $T_{\text{aph,mort,opt}}$	Temperatures for aphid mortality rate constants (9)	0, 15 °C
T_0, T_{ref}, T_0'	Temperatures defining the standard temperature function [Eq (17)]	0, 20, 45 °C
$T_{\text{0aph,dev}}$, $T_{\text{0aph,dev}}'$; $T_{\text{0aph,fec}}$, $T_{\text{0aph,fec}}'$	Temperatures modifying the standard temperature function [Eq (17)], for development [Eq (35)]; fecundity [Eq (18)]	0 (unchanged), 30; 3, 30 °C
$v_{\text{alaa,max20}}$, $v_{\text{apta,max20}}$	Maximum volume intake rate of phloem sap by alate, apterous adults at 20 °C [Eqs (65), (19)]	0.2×10^{-6} m^3 aphid^{-1} d^{-1}
**Other aphid sub-model variables**		
**Symbol**	**Definition**	**Values and Units**
a_{lai}, a_{pti}, a_{la}, a_{pt}, a_{ph}	Alate, apterous instar totals, alate, apterous totals, total aphids [Eq (4)]	Aphid number stem^{-1}
$A_{\text{leaf,aph}}$	Leaf area colonized by aphids [Eq (5)]	m^2 leaf stem^{-1}
C_{ala}, C_{aph}, C_{apt}, N_{ala}, N_{aph}, N_{apt}	Aphid C and N per stem for alate, total and apterous aphids [Eq (6)]	kg aphid C, N stem^{-1}
$\mathrm{d}a_{\text{ph}}/\mathrm{d}t$	Rate of change of total aphid number [Eq (101), *cf.* Eq (102)]	Aphids stem^{-1} d^{-1}
$\mathrm{d}C_{\text{aph}}/\mathrm{d}t$, $\mathrm{d}C_{\text{aph,m2}}/\mathrm{d}t$, $\mathrm{d}N_{\text{aph}}/\mathrm{d}t$, $\mathrm{d}N_{aph,m2}/\mathrm{d}t$	Rate of change of total aphid C, N [Eqs (113), (123); *cf.* Eqs (114), (124)]	kg aphid C, N stem^{-1}, kg aphid C, N m^{-2}

continued on next page

Symbol	Definition	Values and Units
$f(T)$	Standard biological temperature function [Eq (17)]	
$f_{\text{alaa→ala1}}$, $f_{\text{alaa→apt1}}$, $f_{\text{apta→ala1}}$, $f_{\text{apta→apt1}}$	Fractions of alate, apterous offspring which are alate or apterous [Eqs (73), (28)	
f_{fecalaa}, f_{fecapta}	Fractional fecundity of alate, apterous adults, Eqs (74), (29)]	d^{-1}
$f_{\text{Nalaa→ala1}}$, $f_{\text{Talaa→ala1}}$, $f_{\rho\text{alaa→ala1}}$; $f_{\text{Napta→ala1}}$, $f_{\text{Tapta→ala1}}$, $f_{\rho\text{apta→ala1}}$	Fractions of alate, apterous offspring which are alate, as affected by nutrition (N), temperature (T) and aphid density (ρ) [Eqs (70)–(72); (25)–(27)]	
$f_{\text{Oalaa→emi}}$, $f_{\text{Oalaa→mort}}$	Fractional output fluxes of alate adults to emigration and mortality [Eq (99)]	
$f_{\text{TNaph,dev}}$	Combined effects of temperature and N on development [Eq (37)]	
$f(T)$, $f(T = T_{\text{air}})$	Temperature function (17), used to modify emigration [Eq (61)]	
$I_{\text{ala}\jmath}$, $I_{\text{apt}\jmath}$, $\jmath = 1 \ldots 4$	Inputs to alate, apterous instar pools [Eqs (81), (41)]	aphids stem^{-1} d^{-1}
I_{alaa}, I_{apta}	Total inputs to alate, apterous adult pools [Eqs (57), (8)]	aphids stem^{-1} d^{-1}
$I_{\text{ala4→alaa}}$, $I_{\text{imm→alaa}}$	Inputs to alate adult pools from development [Eq (83)], immigration [Eq (56) but see Eq (64)]	aphids stem^{-1} d^{-1}
$I_{\text{alaa→apt1}}$, $I_{\text{alaa→ala1}}$; $I_{\text{apta→apt1}}$, $I_{\text{apta→ala1}}$	Inputs to 1th instar apterous and alate pools from alate, apterous progeny [Eqs (74), (30)]	aphids stem^{-1} d^{-1}
$I_{\text{Cala4→a}}$, $I_{\text{Nala4→a}}$; $I_{\text{Capt4→a}}$, $I_{\text{Napt4→a}}$	Inputs of C, N from 4th instar into alate and apterous adult aphids [Eqs (58), (8)]	aphids stem^{-1} d^{-1}
I_{Caph}, $I_{\text{Caph,m2}}$, I_{Naph}, $I_{\text{Naph,m2}}$	Total C, N inputs to aphids [Eqs (105), (117)]	kg aphid C, N stem^{-1} d^{-1}, kg aphid C, N m^{-2} d^{-1}
$I_{\text{Caph→surf-li}}$, $I_{\text{Naph→surf-li}}$	Aphid C, N inputs to the surface litter cellulose + lignin pools [Eq (126)]	kg C, N m^{-2} d^{-1}
$I_{\text{Caph→surf-li,cel}}$, $I_{\text{Caph→surf-li,lig}}$, $I_{\text{Caph→surf-li,met}}$	C inputs to surface litter cellulose, lignin, metabolic pools [Eqs (127), (125)]	kg C m^{-2} d^{-1}
$I_{\text{Cimm→alaa}}$, $I_{\text{Cimm→alaa,m2}}$, $I_{\text{Nimm→alaa}}$, $I_{\text{Nimm→alaa,m2}}$	Input of C, N from immigrating alate adults [Eqs (104), (116)]	kg C, N stem^{-1} d^{-1}, kg C, N m^{-2} d^{-1}
$I_{\text{Cphloem→alaa,aphid}}$, $I_{\text{Cphloem→apta,aphid}}$, $I_{\text{Nphloem→alaa,aphid}}$, $I_{\text{Nphloem→apta,aphid}}$	C, N intake rates per alate, apterous adult aphid [Eqs (69), (24), (67), (22)]	kg C, N aphid^{-1} d^{-1}
$I_{\text{Cphloem→ala}\jmath}$, $I_{\text{Cphloem→apt}\jmath}$, $I_{\text{Nphloem→ala}\jmath}$, $I_{\text{Nphloem→apt}\jmath}$, $\jmath = 1, \ldots, 4$	C inputs required for development by 4 alate instars ($\jmath = 1, \ldots, 4$), all 4 alate instars summed (alai) [Eqs (92), (90)], 4 apterous instars ($\jmath = 1, \ldots, 4$) all 4 apterous instars summed (apti) [Eqs (52), (50)]	kg C stem^{-1} d^{-1}
$I_{\text{Cphloem→alai}}$, $I_{\text{Cphloem→apti}}$, $I_{\text{Nphloem→alai}}$, $I_{\text{Nphloem→apti}}$	Total C, N inputs from phloem to all 4 alate, apterous instars [Eqs (90), (50), (89), (49)]	kg C, N stem^{-1} d^{-1}
$I_{\text{Cphloem→ala}\jmath}$; $I_{\text{Cphloem→alai}}$; $I_{\text{Cphloem→apt}\jmath}$, $I_{\text{Cphloem→apti}}$, $\jmath = 1, \ldots, 4$	C inputs required for development by alate, apterous instars, all 4 instars [Eqs (92), (90), (52), (50)]	kg C stem^{-1} d^{-1}

continued on next page

Symbol	Definition	Values and Units
$I_{\text{Cphloem}\to\text{aph}}$, $I_{\text{Cphloem}\to\text{aph},m2}$, $I_{\text{Nphloem}\to\text{aph}}$, $I_{\text{Nphloem}\to\text{aph},m2}$	Total C, N input to aphids from phloem [Eqs (103), (115)]	kg C, N stem^{-1} d^{-1}, kg C, N m^{-2} d^{-1}
$I_{\text{Npalaa,max}}$, $I_{\text{Npapta,max}}$	Maximum processing N intake rate per alate, apterous adult aphid [Eqs (66), (21)]	kg N aphid^{-1} d^{-1}
$I_{\text{Nphloem,valaa,max}}$, $I_{\text{Nphloem,vapta,max}}$	Maximum phloem volume-limited N intake rate per alate, apterous adult aphid [Eqs (65), (20)]	kg N aphid^{-1} d^{-1}
$I_{\text{Cphloem}\to\text{alaa}}$, $I_{\text{Cphloem}\to\text{apta}}$, $I_{\text{Nphloem}\to\text{alaa}}$, $I_{\text{Nphloem}\to\text{apta}}$	C, N inputs from phloem to alate, apterous adults [Eqs (69), (24)]	kg C, N aphid^{-1} d^{-1}
$I_{\text{Nphloem}\to\text{ala}\jmath}$, $I_{\text{Nphloem}\to\text{apt}\jmath}$, $\jmath = 1,\dots,4$	N inputs from phloem to alate, apterous instars [Eqs (88), (48)]	kg N aphid^{-1} d^{-1}
$k_{\text{ala}\jmath\to\jmath+1}$, $k_{\text{apt}\jmath\to\jmath+1}$, $\jmath = 1,\dots,3$, $k_{\text{ala4}\to\text{a}}$, $k_{\text{apt4}\to\text{a}}$	Developmental rate parameters [Eqs (79), (38)]	d^{-1}
$k_{\text{alaa}\to\text{emi}}$	Rate parameter for emigration of adult alates [Eq (61)]	d^{-1}
$k_{\text{aph,mort}}$	Mortality rate [Eq (11)]	d^{-1}
$k_{\text{aph,Nmort}}$	Nutrition-dependent mortality rate [Eq (10)]	d^{-1}
$k_{\text{aph,Tmort}}$	Temperature-dependent mortality rate	d^{-1}
$O_{\text{ala}\jmath\to\jmath+1}$, $O_{\text{apt}\jmath\to\jmath+1}$, $\jmath = 1,\dots,3$, $O_{\text{ala4}\to\text{a}}$, $O_{\text{apt4}\to\text{a}}$	Developmental outputs from alate, apterous instar pools [Eqs (82), (42)]	aphids stem^{-1} d^{-1}
$O_{\text{ala}\jmath\to\text{mort}}$, $O_{\text{apt}\jmath\to\text{mort}}$, $O_{\text{ala}\jmath\to\text{prune}}$, $O_{\text{apt}\jmath\to\text{prune}}$, $\jmath = 1,\dots,4$	Outputs from alate, apterous instar pools to mortality [Eqs (84), (44)], pruning [Eqs (85), (45)]	aphids stem^{-1} d^{-1}
$O_{\text{ala}\to\text{mort}}$, $O_{ala\to\text{prune}}$, $O_{\text{apt}\to\text{mort}}$, $O_{\text{apt}\to\text{prune}}$	Total outputs from alate, apterous aphids to mortality [Eqs (84), (44)] and pruning [Eqs (85), (45)]	aphids stem^{-1} d^{-1}
$O_{\text{ala}\jmath}$, $O_{\text{apt}\jmath}$, $\jmath = 1,\dots,4$	Total outputs from alate; apterous instar pools [Eqs (86), (46)]	aphids stem^{-1} d^{-1}
O_{alaa}, O_{apta}	Total output of alate, apterous adults [Eqs (63), (15)]	aphids stem^{-1} d^{-1}
$O_{\text{alaa}\to\text{ala1}}$, $O_{\text{alaa}\to\text{apt1}}$, $O_{\text{apta}\to\text{ala1}}$, $O_{\text{apta}\to\text{apt1}}$	Output of alate and apterous 1st instar nymphs from alate, apterous adult progeny [Eqs (74), (29), (30)]	aphids stem^{-1} d^{-1}
$O_{\text{alaa}\to\text{emi}}$	Output of alate adults to emigration [Eq (62)]	aphids stem^{-1} d^{-1}
$O_{\text{alaa}\to\text{mort}}$, $O_{\text{alaa}\to\text{prune}}$, $O_{\text{apta}\to\text{mort}}$, $O_{\text{apta}\to\text{prune}}$	Outputs of alate, apterous adults to mortality [Eqs (59), (12)], pruning [Eqs (60), (14)]	aphids stem^{-1} d^{-1}
$O_{\text{aph}\to\text{fec}}$, $O_{\text{aph}\to\text{mort}}$, $O_{\text{aph}\to\text{prune}}$	Total aphid outputs from fecundity, to mortality and pruning [Eqs (100), (96), (85)]	aphids stem^{-1} d^{-1}
$O_{\text{Calaa}\to\text{ala1}}$, $O_{\text{Calaa}\to\text{apt1}}$, $O_{\text{Nalaa}\to\text{ala1}}$, $O_{\text{Nalaa}\to\text{apt1}}$, $O_{\text{Nalaa}\to\text{axx1}}$, $O_{\text{Calaa}\to\text{hon}}$	C, N fluxes from alate adults to offspring [Eqs (77), (75)] and C flux to honeydew [Eq (78)]	kg C, N stem^{-1} d^{-1}
$O_{\text{Calaa}\to\text{emi}}$, $O_{\text{Nalaa}\to\text{emi}}$, $O_{\text{Calaa}\to\text{emi,m2}}$, $O_{\text{Nalaa}\to\text{emi,m2}}$	C, N outputs with emigrating alates [per stem Eq (62)] [per unit ground area Eqs (109), (119)]	kg C, N stem^{-1} d^{-1}, kg C, N m^{-2} d^{-1}
$O_{\text{Calaa}\to\text{mort}}$, $O_{\text{Nalaa}\to\text{mort}}$, $O_{\text{Capta}\to\text{mort}}$, $O_{\text{Napta}\to\text{mort}}$	C, N outputs of alate, apterous adults to mortality [Eqs (59), (13)]	kg aphid C, N stem^{-1} d^{-1}

continued on next page

Symbol	Definition	Values and Units
$O_{\text{Calaa}\to\text{prune}}$, $O_{\text{Nalaa}\to\text{prune}}$, $O_{\text{Capta}\to\text{prune}}$, $O_{\text{Napta}\to\text{prune}}$	C, N fluxes of alate, apterous adults to pruning [Eqs (60), (14)]	kg aphid C, N stem^{-1} d^{-1}
$O_{\text{Calai}\to\text{hon}}$, $O_{\text{Capti}\to\text{hon}}$	C outputs from alate, apterous instars to honeydew [Eqs (93), (53)]	kg C stem^{-1} d^{-1}
O_{Caph}, $O_{\text{Caph,m2}}$, O_{Naph}, $O_{\text{Naph,m2}}$	Total aphid C, N outputs [Eqs (112), (122)]	kg C, N stem^{-1} d^{-1}, kg C, N m^{-2} d^{-1}
$O_{\text{Caph}\to\text{CO2}}$, $O_{\text{Caph}\to\text{CO2,m2}}$	Total aphid respiration, [Eq (106)]	kg C stem^{-1} d^{-1}, kg C m^{-2} d^{-1}
$O_{\text{Caph}\to\text{hon}}$, $O_{\text{Caph}\to\text{hon,m2}}$	Total C outputs to honeydew [Eq (107)]	kg C stem^{-1} d^{-1}, kg C m^{-2} d^{-1}
$O_{\text{Caph}\to\text{mort}}$, $O_{\text{Caph}\to\text{mort,m2}}$, $O_{\text{Naph}\to\text{mort}}$, $O_{\text{Naph}\to\text{mort,m2}}$	Total C, N outputs to mortality [Eqs (108), (118)]	kg C, N stem^{-1} d^{-1}, kg C, N m^{-2} d^{-1}
$O_{\text{Caph}\to\text{prune}}$, $O_{\text{Caph}\to\text{prune,m2}}$, $O_{\text{Naph}\to\text{prune}}$, $O_{\text{Naph}\to\text{prune,m2}}$	Total C, N outputs to pruning [Eqs (110), (120)]	kg C, N stem^{-1} d^{-1}, kg C, N m^{-2} d^{-1}
$O_{\text{Caph}\to\text{th,m2}}$, $O_{\text{Naph}\to\text{th,m2}}$	Total C, N outputs from thinning [Eqs (111), (121)]	kg C, N m^{-2} d^{-1}
$O_{\text{Capta}\to\text{ala1}}$, $O_{\text{Capta}\to\text{apt1}}$, $O_{\text{Capta}\to\text{axx1}}$, $O_{\text{Capta}\to\text{hon}}$; $O_{\text{Napta}\to\text{ala1}}$, $O_{\text{Napta}\to\text{apt1}}$, $O_{\text{Napta}\to\text{axx1}}$	C, N fluxes from apterous adults to offspring [Eqs (33), (31)] and (C) to honeydew [Eq (34)]	kg C, N stem^{-1} d^{-1}
$O_{\text{Cphloem}\to\text{alai}}$, $O_{\text{Cphloem}\to\text{apti}}$	C outputs from phloem to support instar development, [Eqs (92), (52)]	kg C stem^{-1} d^{-1}
$O_{\text{Calai}\to\text{mort}}$, $O_{\text{Nalai}\to\text{mort}}$, $O_{\text{Calai}\to\text{prune}}$, $O_{\text{Nalai}\to\text{prune}}$; $O_{\text{Capti}\to\text{mort}}$, $O_{\text{Napti}\to\text{mort}}$, $O_{\text{Capti}\to\text{prune}}$, $O_{\text{Napti}\to\text{prune}}$	C, N outputs from alates, apterous instars to mortality, pruning [Eqs (94), (95), (54), (55)]	kg C, N stem^{-1} d^{-1}
R_{alaa}, R_{alai}, R_{apta}, R_{apti}	Respiration from alate, apterous adults, all instars [Eqs (76), (91), (32), (51)]	kg C stem^{-1} d^{-1}
s_{urvival}	Probability of survival of a 1st instar apterous aphid [Eq (40)]	
$v_{\text{phloem}\to\text{alaa}}$, $v_{\text{phloem}\to\text{apta}}$	Phloem volume sap intake rate by alate, apterous adults [Eqs (68), (23)]	m^3 aphid^{-1} d^{-1}
$v_{\text{alaa,max}}$, $v_{\text{apta,max}}$	Maximum volume intake rate of phloem sap by alate, apterous adults [Eqs (65), (19)]	m^3 aphid^{-1} d^{-1}
ρ_{ala}, ρ_{aph}, ρ_{apt}	Aphid densities of alates, all aphids, apterous [Eq (5)]	Aphids (m^2 leaf)$^{-1}$

	**Forest sub-model variables and parameters**	
**Symbol**	**Definition**	**Values and Units**
A_{leaf}	Leaf area per stem [Eq (1)]	m^2 stem^{-1}
$A_{\text{leaf,aph}}$	Leaf area colonized by aphids [Eq (5)]	m^2 stem^{-1}
C_{le}, N_{le}	Substrate C, N concentrations in foliage (*leaf*) (*le*) [Eq (2)]	kg substrate C, N (kg structural DM)$^{-1}$
C_{phloem}, N_{phloem}	C, N substrate concentrations in phloem sap [Eq (2)]	kg substrate C, N (m^3 phloem sap)$^{-1}$

continued on next page

Symbol	Definition	Values and Units
C_{sys}	Total C sequestered by system [Fig 7]	kg C (m^2 ground)$^{-1}$
$k_{\mathrm{le \to prune}}$	Foliage (*le*) pruning rate [Eq (14)]	0 d^{-1}
L_{AI}	Leaf area index [Eq (1)]	(m^2 leaf) (m^2 ground)$^{-1}$
n_{stems}	Number of stems per unit ground area [efm state variable, Eq (1)]	Number of stems (m^2 ground)$^{-1}$
$O_{\mathrm{nstems,th}}$	Output of stems due to management thinning [Eq (1)]. Section 4.2	Number of stems m^{-2} d^{-1}
r_{CNphloem}	Phloem C:N ratio [Eq (3)]	kg C (kg N)$^{-1}$
Y_{C}	Yield class [Fig 7]	m^3 ha^{-1} y^{-1}
**Soil sub-model inputs**		
**Symbol**	**Definition**	**Values and Units**
$I_{\mathrm{Caph \to surf\text{-}li}}$, $I_{\mathrm{Naph \to surf\text{-}li}}$	C, N inputs to surface litter pools [Eq (126)]	kg C, N m^{-2} d^{-1}
$I_{\mathrm{Caph \to surf\text{-}li,cel}}$, $I_{\mathrm{Naph \to surf\text{-}li,cel}}$	C, N inputs to surface litter cellulose C, N pools [Eqs (127), (128)]	kg C, N m^{-2} d^{-1}
$I_{\mathrm{Caph \to surf\text{-}li,lig}}$, $I_{\mathrm{Naph \to surf\text{-}li,lig}}$	C, N inputs to surface litter lignin C, N pools [Eqs (127), (128)]	kg C, N m^{-2} d^{-1}
$I_{\mathrm{Caph \to surf\text{-}li,met}}$, $I_{\mathrm{Naph \to surf\text{-}li,met}}$	C, N inputs to surface litter metabolic C, N pools [Eqs (125), (128)]	kg C, N m^{-2} d^{-1}
**Environmental variables which impact directly on the aphid sub-model**		
**Symbol**	**Definition**	**Values and Units**
T_{air}	Air temperature varies diurnally and seasonally, assumed to be aphid temperature. It affects emigration [Eq (61)], mortality [Eq (9)], fecundity [Eq (18)], alate:apterous ratio in offspring [Eq (25)]	˚C

References

1. Wiktelius S, Pettersson J. Simulations of bird cherry—oat aphid population dynamics: A tool for developing strategies for breeding aphid-resistant plants. Agriculture, Ecosystems & Environment. 1985;14(3-4):159–170.

2. Ekbom B, Wiktelius S, Chiverton P. Can polyphagous predators control the bird cherry-oat aphid (*Rhopalosiphum padi*) in spring cereals?: A simulation study. Entomologia Experimentalis et Applicata. 1992;65(3):215–223.

3. Morgan D. Population dynamics of the bird cherry-oat aphid, *Rhopalosiphum padi* (L.), during the autumn and winter: a modelling approach. Agricultural and Forest Entomology. 2000;2(4):297–304.

4. Newman JA, Gibson DJ, Parsons AJ, Thornley JHM. How predictable are aphid population responses to elevated CO_2? Journal of Animal Ecology. 2003;72(4):556–566.

5. Newman JA. Climate change and cereal aphids: the relative effects of increasing CO_2 and temperature on aphid population dynamics. Global Change Biology. 2004;10(1):5–15.

6. Newman JA. Climate change and the fate of cereal aphids in Southern Britain. Global Change Biology. 2005;11(6):940–944.

7. Newman JA. Using the output from global circulation models to predict changes in the distribution and abundance of cereal aphids in Canada: a mechanistic modeling approach. Global Change Biology. 2006;12(9):1634–1642.

8. Hoover JK, Newman JA. Tritrophic interactions in the context of climate change: a model of grasses, cereal aphids and their parasitoids. Global Change Biology. 2004;10(7):1197–1208.

9. Parry HR, Evans AJ, Morgan D. Aphid population response to agricultural landscape change: a spatially explicit, individual-based model. Ecological Modelling. 2006;199(4):451–463.

10. Thackray DJ, Diggle AJ, Jones RAC. BYDV PREDICTOR: a simulation model to predict aphid arrival, epidemics of barley yellow dwarf virus and yield losses in wheat crops in a Mediterranean-type environment. Plant Pathology. 2009;58(1):186–202.

11. Ma ZS, Bechinski EJ. A survival-analysis-based simulation model for Russian wheat aphid population dynamics. Ecological Modelling. 2008;216(3-4):323–332.

12. Carter N, Rabbinge R. Simulation models of the population development of *Sitobion avenae*. IOBC/WPRS Bulletin. 1980;3:93–98.

13. Carter N, Dixon AFG, Rabbinge R. Cereal Aphid Populations: Biology, Simulation and Prediction. Pudoc; 1982.

14. Rabbinge R, Ankersmit G, Pak G. Epidemiology and simulation of population development of *Sitobion avenae* in winter wheat. Netherlands Journal of Plant Pathology. 1979;85(5):197–220.

15. van Roermund H, Groot J, Rossing W, Rabbinge R. Calculation of aphid damage in winter wheat, using a simulation model. Mededelingen-Universiteit Gent, Faculteit Landbouwkundige en Toegepaste Biologische Wetenschappen. 1986;51(3a):1125–1130.

16. Skirvin D, Perry J, Harrington R. The effect of climate change on an aphid–coccinellid interaction. Global Change Biology. 1997;3(1):1–11.

17. Rossing W. Simulation of damage in winter wheat caused by the grain aphid *Sitobion avenae*. 2. Construction and evaluation of a simulation model. Netherlands Journal of Plant Pathology. 1991;97(1):25–54.

18. Freier B, Triltsch H, Rossberg D. GTLAUS-A model of wheat-cereal aphid-predator interaction and its use in complex agroecological studies. Journal of Plant Diseases and Protection. 1996; p. 543–554.

19. Gosselke U, Triltsch H, Rossberg D, Freier B. GETLAUS01—the latest version of a model for simulating aphid population dynamics in dependence on antagonists in wheat. Ecological Modelling. 2001;145(2-3):143–157.

20. Duffy C, Fealy R, Fealy RM. An improved simulation model to describe the temperature-dependent population dynamics of the grain aphid, *Sitobion avenae*. Ecological Modelling. 2017;354:140–171.

21. van Roermund H, Groot J, Rossing W, Rabbinge R. Stimulation of aphid damage in winter wheat. NJAS Wageningen Journal of Life Sciences. 1986;34(4):488–493.

22. Hughes R, Gilbert N. A model of an aphid population–a general statement. The Journal of Animal Ecology. 1968; p. 553–563.

23. Gilbert N, Hughes R. A model of an aphid population–three adventures. The Journal of Animal Ecology. 1971; p. 525–534.

24. Raworth D. Population dynamics of the cabbage aphid, *Brevicoryne Brassicae* (Homoptera: Aphididae) at Vancouver, British Columbia: V. A simulation model. The Canadian Entomologist. 1984;116(6):895–911.

25. Gutierrez A, Baumgärtner J, Summers C. Multitrophic models of predator–prey energetics: I. Age-specific energetics models–pea aphid *Acyrthosiphon pisum* (Homoptera: Aphididae) as an example. The Canadian Entomologist. 1984;116(7):923–932.

26. Snyder WE, Ives AR. Interactions between specialist and generalist natural enemies: parasitoids, predators, and pea aphid biocontrol. Ecology. 2003;84(1):91–107.

27. Kindlmann P. A model of aphid population with age structure. In: Capasso V, Grosso E, Paveri-Fontana SL, editors. Mathematics in Biology and Medicine. vol. 57 of Lecture Notes in Biomathematics. Berlin, Heidelberg: Springer; 2007. p. 72–77.

28. Xia JY, Wang J, Cui JJ, Leffelaar PA, Rabbinge R, van der Werf W. Development of a stage-structured process-based predator–prey model to analyse biological control of cotton aphid, *Aphis gossypii*, by the sevenspot ladybeetle, *Coccinella septempunctata*, in cotton. Ecological Complexity. 2018;33:11–30.

29. Gutierrez A, Havenstein D, Nix H, Moore P. The ecology of *Aphis craccivora* Koch and subterranean clover stunt virus in south-east Australia. II. A model of cowpea aphid populations in temperate pastures. Journal of Applied Ecology. 1974; p. 1–20.

30. Gutierrez A, Nix H, Havenstein D, Moore P. The ecology of *Aphis craccivora* Koch and subterranean clover stunt virus in south-east Australia. III. A regional perspective of the phenology and migration of the cowpea aphid. Journal of Applied Ecology. 1974; p. 21–35.

31. Whalon ME, Smilowitz Z. Temperature-dependent model for predicting field populations of green peach aphid, *Myzus persicae* (Homoptera: Aphididae). The Canadian Entomologist. 1979;111(9):1025–1032.

32. Tamaki G, Long GE. Predator complex of the green peach aphid on sugarbeets: expansion of the predator power and efficacy model. Environmental Entomology. 1978;7(6):835–842.

33. Ro TH, Long GE. GPA-Phenodynamics, a simulation model for the population dynamics and phenology of green peach aphid in potato: formulation, validation, and analysis. Ecological Modelling. 1999;119(2-3):197–209.

34. Bertschinger L, Keller ER, Gessler C. Development of EPIVIT, a simulation model for contact-and aphid-transmitted potato viruses. Phytopathology. 1995;85(7):801–814.

35. Miksanek JR, Heimpel GE. A matrix model describing host–parasitoid population dynamics: The case of *Aphelinus certus* and soybean aphid. PLoS One. 2019;14(6):e0218217.

36. Day KR, Ayres MP, Harrington R, Kidd NA. Interannual dynamics of aerial and arboreal green spruce aphid populations. Population Ecology. 2010;52(2):317–327.

37. Barlow N. Modelling aphid populations. New Zealand Journal of Ecology. 1981; p. 52–55.

38. Dixon A, Barlow N. Population regulation in the lime aphid. Zoological Journal of the Linnean Society. 1979;67(3):225–237.

39. Barlow ND, Dixon AFG. Simulation of lime aphid population dynamics. Centre for Agricultural Publishing and Documentation; 1980.

40. Gilbert N, Gutierrez A. A plant-aphid-parasite relationship. The Journal of Animal Ecology. 1973; p. 323–340.

41. Augustin DC, Fineberg MS, Johnson BB, Linebarger RN, Sansom FJ, Strauss JC. The SCI Continuous System Simulation Language (CSSL). Simulation. 1967;9:281–303.

42. Thornley JHM, France J. Mathematical Models in Agriculture: Quantitative Methods for the Plant, Animal and Ecological Sciences. CABI; 2007.

43. Thornley JHM. Modelling forest ecosystems: the Edinburgh Forest Model. In: Reynolds K, Thomson A, K ohl M, Shannon M, Ray D, Rennolls K, editors. Sustainable Forestry: From Monitoring and Modelling to Knowledge Management and Policy Science. Wallingford Oxon OX10 8DE UK: CAB International; 2007. p. 342–373.

44. Thornley JHM. A transport-resistance model of forest growth and partitioning. Annals of Botany. 1991;68(3):211–226.

45. Thornley JHM. Grassland Dynamics: An Ecosystem Simulation Model. CAB International; 1998.

46. Thornley JHM. Modelling water in crops and plant ecosystems. Annals of Botany. 1996;77(3):261–275.

47. Thornley JHM, Cannell MGR. Temperate forest responses to carbon dioxide, temperature and nitrogen: a model analysis. Plant, Cell & Environment. 1996;19(12):1331–1348.

48. Cannell MGR, Thornley JHM, Mobbs DC, Friend AD. UK conifer forests may be growing faster in response to increased N deposition, atmospheric CO_2 and temperature. Forestry: An International Journal of Forest Research. 1998;71(4):277–296.

49. Thornley JHM. Dynamic model of leaf photosynthesis with acclimation to light and nitrogen. Annals of Botany. 1998;81(3):421–430.

50. Cannell MGR, Thornley JHM. Modelling the components of plant respiration: some guiding principles. Annals of Botany. 2000;85(1):45–54.

51. Thornley JHM, Cannell MGR. Managing forests for wood yield and carbon storage: a theoretical study. Tree Physiology. 2000;20(7):477–484.

52. Douglas AE. Phloem-sap feeding by animals: problems and solutions. Journal of Experimental Botany. 2006;57(4):747–754.

53. Thompson GA, Schulz A. Macromolecular trafficking in the phloem. Trends in Plant Science. 1999;4(9):354–360.

54. Parry W. The effect of needle age on the acceptability of Sitka spruce needles to the aphid, *Elatobium abietinum* (Walker). Oecologia. 1976;23(4):297–313.

55. Dixon AFG. Aphid Ecology. 2nd ed. Chapman & Hall; 1998.

56. Dean GJ. Effect of temperature on the cereal aphids *Metopolophium dirhodum* (Wlk.), *Rhopalosiphum padi* (L.) and Macrosiphum avenue (F.)(Hem., Aphididae). Bulletin of Entomological Research. 1974;63(3):401–409.

57. De Barro PJ. The role of temperature, photoperiod, crowding and plant quality on the production of alate viviparous females of the bird cherry-oat aphid, *Rhopalosiphum padi*. Entomologia Experimentalis et Applicata. 1992;65(3):205–214.

58. Fisher M. Morph determination in *Elatobium abietinum* (Walk.) the green spruce aphid. Norwich, UK: Dissertation, University of East Anglia; 1982.
